# Genetic diversity analysis of tropical and sub-tropical maize germplasm for *Striga* resistance and agronomic traits with SNP markers

**DOI:** 10.1371/journal.pone.0306263

**Published:** 2024-08-06

**Authors:** Emeline N. Dossa, Hussein Shimelis, Admire I. T. Shayanowako

**Affiliations:** School of Agricultural, Earth and Environmental Sciences, University of KwaZulu-Natal, Scottsville, Pietermaritzburg, South Africa; KGUT: Graduate University of Advanced Technology, ISLAMIC REPUBLIC OF IRAN

## Abstract

*Striga hermonthica (Sh)* and *S*. *asiatica (Sa)* are major parasitic weeds limiting cereal crop production and productivity in sub-Saharan Africa (SSA). Under severe infestation, *Striga* causes yield losses of up to 100%. Breeding for *Striga*-resistant maize varieties is the most effective and economical approach to controlling the parasite. Well-characterized and genetically differentiated maize germplasm is vital to developing inbred lines, hybrids, and synthetic varieties with *Striga* resistance and desirable product profiles. The objective of this study was to determine the genetic diversity of 130 tropical and sub-tropical maize inbred lines, hybrids, and open-pollinated varieties germplasm using phenotypic traits and single nucleotide polymorphism (SNP) markers to select *Striga*-resistant and complementary genotypes for breeding. The test genotypes were phenotyped with *Sh* and *Sa* infestations using a 13x10 alpha lattice design with two replications. Agro-morphological traits and *Striga*-resistance damage parameters were recorded under a controlled environment. Further, high-density Diversity Array Technology Sequencing-derived SNP markers were used to profile the test genotypes. Significant phenotypic differences (P<0.001) were detected among the assessed genotypes for the assessed traits. The SNP markers revealed mean gene diversity and polymorphic information content of 0.34 and 0.44, respectively, supporting the phenotypic variation of the test genotypes. Higher significant variation was recorded within populations (85%) than between populations using the analysis of molecular variance. The Structure analysis allocated the test genotypes into eight major clusters (K  =  8) in concordance with the principal coordinate analysis (PCoA). The following genetically distant inbred lines were selected, displaying good agronomic performance and *Sa* and *Sh* resistance: CML540, TZISTR25, TZISTR1248, CLHP0303, TZISTR1174, TZSTRI113, TZDEEI50, TZSTRI115, CML539, TZISTR1015, CZL99017, CML451, CML566, CLHP0343 and CML440. Genetically diverse and complementary lines were selected among the tropical and sub-tropical maize populations that will facilitate the breeding of maize varieties with *Striga* resistance and market-preferred traits.

## Background

Maize (*Zea maize* L., 2n = 2x = 20) is the key food security crop in sub-Saharan Africa (SSA). However, the mean maize yield in the region is low (<3 t/ha) compared with the global average of 5 to 10 t/ha (FAO, 2022). Low yields are attributable to a plethora of challenges, including biotic (e.g. field and storage pests, plant diseases, and *Striga* infestation) and abiotic (e.g. poor soil health, drought, and heat). *Striga hermonthica (Sh)* and *S*. *asiatica (Sa)* are parasitic weeds that significantly impede cereal crop production in SSA, with yield losses of up to 100% under severe infestation [1].

*Striga hermonthica* is prevalent in most SSA regions, notably in Western, Central, and Eastern Africa, while *Sa* is predominant in Southern Africa [2–4]. Maize is relatively more susceptible to both species than sorghum and pearl millet due to the co-evolution of the latter with *Striga* [5]. *Striga* extracts the host’s metabolites in exchange for phytotoxic compounds, reducing photosynthesis that causes yield loss varying from 10% to 100% [6, 7]. More than 40 million households are affected by the scourge of *Striga* every year across Africa [7, 8]. Several *Striga* control methods have been reported globally. However, the use of *Striga*-resistant cultivars is the most economical, sustainable, and environmentally friendly approach that can be deployed and adopted by small-holder maize producers [9]. The major components of *Striga* resistance/tolerance in maize are high grain yield, reduced *Striga* emergence, and low *Striga* damage symptoms [10].

The genetic base of maize has been enhanced by breeders at the Institute of Tropical Agriculture (IITA), the International Maize and Wheat Improvement (CIMMYT), and national breeding programs for *Striga* resistance and major economic traits [10]. Genetically diverse maize germplasm has been developed and dispatched by IITA and CIMMYT globally for more than three decades [11–13]. The germplasms can be phenotyped in the target production environments for selection and as parents in *Striga* resistance breeding programs by the public and private sectors. Genetic resources of maize selected by the breeders at IITA possess mainly *S*. *hermonthica* resistance. Conversely, CIMMYT-bred lines in East and Southern Africa display drought and heat stress tolerance. *Striga asiatica* is increasingly a major parasitic weed in South and East Africa due to poor soil fertility and drought stress conditions, which are conducive to the proliferation of the parasite and host susceptibility. Reportedly, both species occur in tandem in the major cereal crops [14, 15]. Breeding for *Striga*-resistant maize cultivars is vital for sustainable *Striga* management [3].

*Striga*-resistant maize varieties are bred with major genes conditioning *Sh* resistance. Gene introgression using the tropical genetic resources into locally adapted sub-tropical varieties will enable the suppression of both *Sh* and *Sa* in SSA. Well-characterized and genetically differentiated maize germplasm is vital to developing inbred lines, hybrids, and synthetic varieties with durable *Striga* resistance. Enhanced hybrid vigour is achieved from crosses of inbred lines from complementary heterotic groups [16, 17]. Hence, detailed information on genetic diversity, genetic interrelationships, and heterotic groups is crucial for developing maize cultivars with desirable product profiles.

Various molecular markers have been developed and applied to determine genetic diversity, population structure, quantitative trait loci (QTL), and linkage maps in maize. These include Restriction Fragment Length Polymorphism (RFLP), Random Amplified Polymorphic DNA (RAPDs), Amplified Fragment Length polymorphic (AFLPs), Single Sequence Repeats (SSR), and Single Nucleotide Polymorphisms (SNPs). SNPs have emerged as the markers of choice for genetic diversity analysis and marker-assisted breeding. This is attributed to their low cost per data point, high genomic abundance, locus specificity, co-dominance, the potential for high throughput analysis, and lower genotyping error rates [18]. SNPs can be identified using various protocols, including Genotyping by sequencing (GBS), restriction-associated DNA (RAD), complexity reduction of polymorphic sequences (CRoPS), and diversity arrays technology (DArT). DArT is a sequence-independent, high throughput, reproducible, cost-effective, and whole genome genotyping technology. DArTseq SNP markers have been routinely used in genetic diversity analysis in maize and other crops.

Results using DArTseq SNP markers enabled the selection of parents for breeding [19]. Successful genetic diversity and grouping of pigeonpea [20], cowpea [21], sorghum [22, 23] maize [24, 25] have been reported using DArTseq SNPs. Genetic diversity analysis of *Striga*-resistant maize populations was reported using DArTseq SNP markers. For instance, Badu-Apraku, et al. [19], Yacoubou, *et al*. [26], and Gasura, et al. [6] discerned the genetic diversity and population structure of maize germplasm. Zebire, *et al*. [27] identified suitable testers for *Striga-*resistant lines using DArTseq SNP markers and agronomic traits. Quantitative trait loci conditioning resistance/tolerance to *S*. *hermonthica* have been identified using this marker system [9, 28–31].

In an attempt to select novel inbred lines with *Striga* resistance and morpho-agronomic traits, genetically diverse tropical and sub-tropical maize genotypes were assembled by the University of KwaZulu-Natal’s African Center for Crop Improvement (ACCI) from IITA/Ibadan, CIMMYT/ Zimbabwe, and the National Plant Genetic Resources Centre (NGRC) in South Africa. The genetic diversity and the population structure of the accessions should be characterized to delineate heterotic groups for developing inbred lines, hybrids, and synthetic varieties with *Striga* resistance and desirable product profiles. Therefore, this study aimed to determine the genetic diversity of 130 tropical and sub-tropical maize germplasm using phenotypic traits and single nucleotide polymorphism (SNP) markers to select *Striga*-resistant and complementary genotypes for breeding.

## Materials and methods

### Plant material

A panel of 130 maize germplasm was used for this study. The test genotypes comprised 74 accessions acquired from IITA/Nigeria, 45 from CIMMYT/Zimbabwe, and 10 from the National Plant Genetic Resources Centre (NPGRC)/South Africa (Supplemental Table 1 in S1 File). The population included released tropical inbred lines, hybrids and open-pollinated varieties with *Striga* resistance and sub-tropical varieties bred for their agronomic performance and drought tolerance in South Africa and East Africa. Seeds of *Sa* were collected from Zimbabwe in 2016, while *Sh* seeds were collected from maize-infested fields in Kenya in 2021. The seeds were stored in airtight plastic jars at room temperature in dry conditions.

### Phenotyping

The 130 accessions were phenotyped at the University of Kwazulu-Natal Controlled Environment Facilities (UKZN-CEF) in two seasons (December 2021–April 2022, and August 2022–December 2022). The UKZN CEF is situated at the UKZN College of Agriculture, Engineering, and Science (29.62° S, 30.40° E). Treatments were laid out using a 13 x 10 alpha lattice design with two replications in each *Striga*-infested environment. Two weeks before planting, each pot was infested with a scoop of sand mixed with 0.03 g of 2-year-old *Sa* or *Sh* seed containing approximately 3000 *Striga* seeds [32]. The experimental unit consisted of 4 plastic pots of 5-L capacity, filled with a composted pine bark potting mix for each *Striga* infested environment. Maize and *Striga* parameters were used for phenotyping. Days to 50% silking (DS) was recorded as the number of days taken by 50% of the plants to silk in each plot; days to anthesis (DA), was recorded as the number of days from planting until 50% of the plants have shed pollen; anthesis-silking interval (ASI), was measured as the difference between days to 50% silking and 50% anthesis; plant height (PLHT) and ear height (EHT) were measured as the distance from the base of the plant to the height of the first tassel branch and the node bearing the upper ear, respectively; root lodging (RL) tolerance was recorded as a percentage of plants leaning more than 30° from the vertical; stalk lodging (SLG) tolerance (percentage broken at or below the highest ear node); and ear rot (EROT) was assessed as the number of rotten ears per plant. The number of ears per plant (EPP) was obtained by dividing the total number of ears per plot by the number of plants harvested. Husk cover (HUSK) was rated on a scale of 1 to 5, where 1 = husks tightly arranged and extended beyond the ear tip and 5 = ear tips exposed. Ear aspect (EASP) was recorded on a scale of 1 to 9, where 1 = clean, uniform, large, well-filled ears and 9 = ears with undesirable features. The grain yield per plant (GY/plant) adjusted to a constant moisture of 12.5% was determined as the grain weight (g) from the ears of an individual plant after shelling. This was determined by dividing the grain yield per plot by the number of plants harvested.

The *Striga* parameters were recorded, including the number of emerged *Sa* and *Sh* plants 8 and 10 weeks after planting, denoted as SEC8 and SEC10. Host plant damage was rated 8 and 10 weeks after planting, designated as SDR8 and SDR10 using a visual rating score of 1 to 9 where 1 = no damage, indicating normal plant growth and a high level of tolerance, and 9 = complete collapse or death of the maize plant, i.e., highly susceptible [33].

### Phenotypic data analysis

Before data analysis, the ASI values were standardized and expressed in positive figures using the corrective value (cv) following [34], where cv = 1 –the smallest ASI value. Phenotypic data collected in both *Sh* and *Sa*-infested environments were subjected to Bartlet’s homogeneity of variance test prior to combined analysis of variance (ANOVA) using a lattice procedure in RStudio version 2023. 06.1 (R Core Team, 2023). Genotypes mean comparisons were made at the 5% significance level using Fisher’s least significance difference (LSD). Phenotypic clusters based on the dissimilarity matrix were generated using the Gower method implemented in the “cluster” and “graphics” procedures in R statistical package version 4.2.1 (R Core Team, 2018). Broad sense heritability (H^2^) was computed using DeltaGen [35] with the following formula:

$$(H^{2})=\frac{\sigma^{2}g}{\sigma^{2}g+\frac{\sigma^{2}s}{ns}+\frac{\sigma^{2}r}{nr}+\frac{\sigma^{2}b}{nb}+\frac{\sigma_{Ɛ}^{2}}{ns+nr+nb}}$$

where $\sigma^{2}g,\sigma^{2}s,\;{\sigma^{2}r,\sigma }^{2}b$, and $\sigma_{Ɛ}^{2}$ are the variance components for genotypes, season, replication, block, and the pooled error, respectively, and ns, nr, and nb are the number of seasons, replications, and blocks, respectively. A hierarchical cluster was constructed using the ward D2 method in “cluster” in R package version 4.2.1 (R Core Team, 2018). Cluster analyses were conducted to classify the germplasm and study their genetic relationships.

### DNA extraction and genotyping

The seeds of the 130 accessions were planted in plastic pots filled with a growing medium in a greenhouse at the University of Kwazulu-Natal. Two weeks after planting, the fresh leaves of the three leaves stage were harvested for genomic DNA extraction. Genomic DNA was extracted using the DArTseq protocol as described by Kilian, *et al*. [36]. DNA quality was checked for nucleic acid concentration and purity using a NanoDrop 2000 spectrophotometer (ND-2000 V3.5, NanoDrop Technologies Inc) as described by Desjardins and Conklin [37]. An estimated 20 μl of DNA sample of each genotype with concentrations between 50 and 100 ng ul-1, and absorbances ranging from 1.75 to 2.05 were submitted to Sequential art (SEQAT) (https://www.seqart.net/) in Kenya for high throughput genotyping. The Diversity Array Technology Sequencing (DArTseq) protocol was used for genotyping the samples as previously described by Elshire, *et al*. [38]. SNPs obtained were used for data analysis in this study.

### Genotypic data analysis

#### SNPs filtering

The numerical genotyping output was used for genotypic data analysis. The initial 70197 SNPs were imputed by removing SNPs with >20% missing data and < 5% minor allele frequency (MAF) on the KDCompute server (https://kdcompute.igssafrica.org/kdcompute/). A total of 16000 informative SNP markers and 130 genotypes were used for further analysis after data imputation.

#### Analysis of genetic diversity parameters and genetic relationship among germplasms

The polymorphic information content (PIC), minor allele frequency (MAF), heterozygosity (Ho), and gene diversity (GD) were calculated using RStudio version 4.3.0 (R Core Team, 2023). Analysis of molecular variance (AMOVA), inbreeding coefficient (Fis), and the genetic distance between the individuals were calculated using GenAlex version 6.5 [39].

#### Population structure analysis

The clustering of the 130 genotypes was assessed using the admixture model-based clustering method in Structure software version 2.3.4 [40]. The burn-in period length and the Markov Chain Monte Carlo (MCMC) replications were set at 10,000. The Structure analysis was done for K ranging from 1 to 10 with 5 iterations at each K to determine the optimum number of clusters. The best K value was predicted following the simulation method of Evanno, *et al*. [41] using Structure harvester version 0.6.94 [42], and the bar plot for the optimum K was confirmed through the clustering markov packager across k (CLUMPAK) beta version [43]. Maize genotypes with inferred ancestries ≥ 70% were assigned to a different population, and those ≤ 70% were treated as admixtures. The dendrograms were generated using the genetic dissimilarity matrix using the “phylogenetics” and “evolution” procedures in RStudio version 4.3.0 (R Core Team, 2023).

#### Joint analysis using phenotypic and SNP data

Genetic groups were defined using a combination of the phenotypic and genotypic dissimilarity matrices. The joint matrix was generated by the summation of the genotypic and phenotypic dissimilarity matrices. The phenotypic dissimilarity matrix was generated using Gower’s distance matrix, while the genotypic dissimilarity matrix was based on Jaccard’s coefficients. The groups generated from the phenotypic and genotypic sets were compared using the “viridis” procedure in R version 4.3.0 (R Core Team, 2023), and the similarity of the two dendrograms was assessed using the tanglegram function developed by the “dendextend” R package (R Core Team, 2020).

## 3. Results

### 3.1 Phenotyping

Genotypic variation was significant for all the assessed traits in both *Sa* and *Sh* environments (Table 1). Under *Sa*-infested conditions, testing seasons had a significant effect (P<0.001) on all the traits except for EPP, PLHT, HUSK, and SEC10. Also, significant effects were noted for all traits except for EPP, PLHT, EHT, and HUSK under *Sh*-conditions. Block nested in replication-by-season interaction significantly affected all the assessed traits under both *Sa* and *Sh*-infested environments, except for EPP.

**Table 1:** Analyse of variance and significant tests for yield components and *Striga* parameters of 126 maize genotypes evaluated under *Striga asiatica* and *S*. *hermonthica* infestations.

*S*. *asiatica*															
**Source of variation**	Df	DA	DS	ASI	EPP	PLHT	EHT	HUSK	CL	EASP	GY	SEC8	SEC10	SDR8	SDR10
**Genotypes (G)**	125	9.34***	7.27***	4.06***	6.49***	2.10***	5.82***	3.65***	9.95***	2.47***	4.05***	2.79***	2.19***	2.41***	2.91***
**Seasons (S)**	1.00	273.78***	100.64***	33.01***	0.00	0.27	20.82***	0.00	29.74***	43.79***	54.71***	1518.79***	3.48	56.80***	37.88***
**G x S**	125	0.0666	0.00	0.084	0.00	0.00	0.00	0.06	0.00	0.036	0.00	0.54	0.00	0.00	0.00
**Replications in seasons**	1.00	0.35	0.00	0.44	0.00	0.00	0.00	0.00	0.00	0.39	0.00	51.62***	0.00	0.00	0.00
**Block/(replication x season)**	13	0.46**	9.29**	4.36**	0.00	0.59**	5.73**	0.07**	1.51**	0.08**	0.06**	2.06**	2.69**	2.00**	0.90**
**Error**	238.00	15.40	20.03	12.28	0.03	106.99	0.05	0.28	2.08	3.47	1439.00	1.51	144.80	2.22	2.13
*S*. *hermonthica*															
**Source of variation**	df	DA	DS	ASI	EPP	PLHT	EHT	HUSK	CL	EASP	GY	SEC8	SEC10	SDR8	SDR10
**Genotypes (G)**	125	2.43***	2.29***	1.76***	3.01***	2.17***	1.84***	1.48**	1.47**	2.76***	2.45***	4.97***	2.13***	2.08***	2.24***
**Seasons (S)**	1.00	125.84***	158.08***	32.01***	0.00	3.14	23.37	1.03	17.77***	18.89***	72.91***	3255.33***	78.31***	222.49***	71.81***
**G x S**	125	0.00	0.00	0.00	0.00	0.00	0.00	0.25	0.00	0.00	0.00	0.00	0.00	0.00	0.00
**Replications in seasons**	1.00	0.00	0.00	0.00	0.00	0.00	0.00	0.26	0.00	0.00	0.00	0.00	0.00	0.00	0.00
**Block/(replication x season)**	13	10.73**	7.20**	0.58**	0.00	0.52**	4.70*	3.83**	10.61**	2.82*	5.07**	4.05**	2.00**	2.54**	1.98**
**Error**	233.00	36.00	51.00	15.75	0.03	3.61	0.08	0.49	7.09	5.40	1473.00	1.20	6.44	2.27	1.76

*, **, and *** denote significance at P < 0.05, P < 0.01 and P < 0.001, respectively, Df = degrees of freedom, DA = days to 50% anthesis, DS = days to 50% silking, ASI = anthesis-silking interval, EPP = ear per plant, PLHT = plant height, EHT = ear height, HUSK = husk cover, CL = cob length, EASP = ear aspect, GY = grain yield, SEC8 = *Striga* emergence counts eight weeks after sowing, SEC10 = *Striga* emergence counts ten weeks after sowing, SDR8 = *Striga* damage rating eight weeks after sowing, and SDR10 = *Striga* damage rating 10 weeks after sowing.

Tables 2 and 3 summarize the mean performances of the top 10 inbred lines and check genotypes with high GY under *Sa* and *Sh-*infested conditions, respectively. In a *Sa*-infested environment, the highest variation was exhibited by PLHT, followed by ASI, with a coefficient of variation values of 426.82% and 268.88%, respectively (Table 2). Inbred lines had a mean ASI of 2.77, while the OPV and hybrid checks had mean ASI values of 1.86 and 1.77, respectively (Table 2). The mean yield of the inbred lines ranged from 0.00 g/plant (TZISTR1262) to 277.50 g/plant (CML540). Further, the mean grain yield of the hybrid checks ranged from 00.00 g/plant (Hickory/1421) to 214.00 g/plant (N.Choice/1421). Whereas the OPV checks had mean grain yields varying from 35.00 g/plant ((IWD C3 SYN*2/(White DT STR Syn)) -DT C1) to 169.50 g/plant (NC.QPM/Z.DPLO).

**Table 2:** Mean values for 14 traits of 126 maize genotypes evaluated under *Striga asiatica* infestation, showing the top 10 inbred lines, the top 4 hybrids, and 6 OPVs based on grain yield.

Top 10 inbred lines														
Genotype	DA	DS	ASI	EPP	PLHT (m)	EHT (m)	HUSK (1 to 5)	CL (cm)	EASP (1 to 9)	GY (g/plant)	SEC8	SEC10	SDR8 (1 to 9)	SDR10 (1 to 9)
**CML540**	77.00	81.50	4.50	1.00	2.03	0.77	1.00	11.00	3.50	277.50	4.00	1.50	3.50	3.00
**CML566**	82.50	79.00	-3.50	1.00	2.22	1.15	1.00	12.00	1.50	155.50	4.00	5.50	1.50	1.50
**TZISTR1001**	82.00	82.00	0.00	1.00	2.10	1.28	1.00	11.00	1.50	140.00	4.50	4.50	3.00	2.50
**TZISTR1205**	81.50	75.50	-6.00	1.00	1.85	0.91	1.00	11.00	1.00	114.25	3.50	13.00	3.00	2.50
**TZSTRI115**	77.50	77.00	-0.50	1.00	2.10	1.20	1.00	11.50	1.50	112.50	5.00	2.00	3.50	2.50
**CLHP0350**	75.00	76.00	1.00	1.00	2.35	0.81	3.00	14.00	3.50	102.75	5.00	3.50	2.00	3.50
**CLHP0049**	80.50	78.00	-2.50	1.00	1.25	0.70	1.00	10.00	3.00	101.25	7.00	4.00	1.00	2.50
**CLHP0302**	81.00	80.50	-0.50	1.00	1.76	1.00	3.00	13.25	3.00	98.00	4.50	7.00	2.00	3.50
**CML440**	82.50	76.00	-6.50	1.00	2.36	1.08	1.00	11.00	1.50	96.25	4.50	13.50	3.00	1.50
**CLHP0303**	84.50	83.50	-1.00	1.50	1.87	1.15	1.00	7.25	3.00	92.50	4.50	8.50	3.00	3.00
**Top 4 hybrids and 6 OPVs**														
**N.Choice/1421**	82.00	76.50	-5.50	1.00	1.90	1.03	1.00	13.25	1.50	214.00	5.00	4.00	3.50	3.50
**Shesha/1421**	75.50	72.50	-3.00	1.00	2.03	1.75	1.00	18.75	1.50	165.75	7.50	18.50	2.00	2.00
**B.King/1421**	80.50	78.50	-2.00	1.00	2.05	1.15	2.00	23.50	1.50	157.25	5.00	4.50	1.00	2.00
**ZM1421/DT-STR**	77.00	76.50	-0.50	1.00	2.38	1.10	1.50	10.75	3.00	93.50	2.50	19.00	2.50	2.00
**NC.QPM/Z.DPLO**	71.50	71.50	0.00	1.00	2.25	1.03	0.00	12.50	4.50	154.25	3.00	5.00	3.50	2.00
**Z.diplo-BC4-C3-W/DOGONA-1/Z.diplo-BC4-C3-W**	81.00	83.00	2.00	1.00	2.36	1.05	1.00	11.50	1.50	112.00	6.00	8.50	3.00	3.50
**DTSTR-W SYN13**	85.50	85.50	0.00	1.00	1.25	0.85	1.50	13.00	3.50	107.50	4.50	3.50	1.50	1.00
**TZBSTR**	83.00	83.50	0.50	1.00	2.65	1.30	1.50	14.50	1.00	103.00	6.50	3.00	3.00	2.50
**ZM1423**	69.00	69.50	0.50	1.00	0.85	1.39	1.00	10.50	1.50	99.25	4.50	16.50	5.00	3.00
(2*TZECOMP3DT/WhiteDTSTRSYN) C2	69.00	78.00	9.00	1.00	1.75	0.85	1.50	12.25	2.50	89.00	6.00	0.50	2.00	1.50
**Trial statistics**														
**LSD (5%)**	3.94	4.44	3.69	0.15	10.57	0.23	0.52	1.55	1.93	37.73	1.87	12.44	1.64	1.61
**SEM**	7.64	8.75	7.27	0.21	20.82	0.46	1.02	3.05	3.80	74.31	3.69	24.51	3.23	3.17
**%CV**	4.89	5.60	268.88	14.65	426.82	25.36	41.21	13.63	58.57	56.12	40.91	129.74	50.29	49.50
**Heritability**	0.90	0.94	0.94	0.11	0.11	0.96	0.96	0.97	0.96	0.88	0.34	0.01	0.11	0.16

DA = days to 50% anthesis, DS = days to 50% silking, ASI = anthesis-silking interval, EPP = ear per plant, PLHT = plant height, EHT = ear height, HUSK = husk cover, CL = cob length, EASP = ear aspect, GY = grain yield, SEC8 = *Striga* emergence counts eight weeks after sowing, SEC10 = *Striga* emergence counts ten weeks after sowing, SDR8 = *Striga* damage rating eight weeks after sowing, and SDR10 = *Striga* damage rating 10 weeks after sowing. LSD = least significant difference, SEM = standard error of the mean, %CV = coefficient of variation, m = meter, cm = centimetre, g = gram.

**Table 3:** Mean responses for 14 traits of 126 maize genotypes evaluated under *Striga hermonthica* infestation, showing the top 10 inbred lines, the top 4 hybrids, and 6 OPVs.

Top 10 lines														
Genotypes	DA	DS	ASI	EPP	PLHT (m)	EHT (m)	HUSK (1 to 5)	CL (cm)	EASP (1 to 9)	GY (g/plant)	SEC8	SEC10	SDR8 (1 to 9)	SDR10 (1 to 9)
**CML304**	79.25	79.63	0.38	1.00	1.44	0.75	1.00	12.00	4.75	151.00	3.18	2.00	4.75	2.75
**TZSTRI101**	90.00	87.50	-2.50	1.00	1.45	0.75	1.00	12.46	3.25	144.00	3.63	6.50	3.75	3.00
**CLHP0404**	74.75	74.25	-0.50	1.00	2.07	0.75	1.00	10.00	6.25	137.35	3.18	5.00	6.00	2.00
**TZISTR1119**	78.75	77.00	-1.75	1.00	1.81	0.95	1.00	10.50	3.75	135.75	3.68	4.50	5.50	3.50
**TZISTR25**	75.75	75.50	-0.25	1.00	2.25	1.05	1.00	12.00	1.25	131.00	3.20	2.00	3.75	2.00
**TZISTR1205**	81.00	83.00	2.00	1.00	2.21	1.00	1.00	9.50	1.75	129.00	4.18	1.00	3.75	2.50
**CML566**	78.00	76.00	-2.00	1.00	2.07	1.20	1.00	12.00	1.25	127.00	2.25	4.50	1.75	2.75
**TZISTR1001**	79.63	78.25	-1.38	1.00	2.10	1.03	1.00	11.50	1.75	120.00	2.70	1.50	3.50	2.50
**TZISTR1174**	79.25	79.63	0.38	1.00	1.44	0.75	1.00	12.00	4.75	151.00	3.18	2.00	4.75	2.75
**TZSTRI113**	74.50	73.00	-1.50	1.00	1.41	0.90	1.00	9.00	1.75	111.75	2.68	3.50	3.75	2.75
**Top 4 hybrids and 6 OPVs**														
**N.Choice/1421**	81.25	75.25	-6.00	1.00	1.62	0.85	1.50	10.96	1.75	133.25	3.13	3.50	4.00	3.00
**Shesha/1421**	71.50	70.75	-0.75	1.00	1.82	0.88	1.50	10.71	1.75	112.25	4.63	2.50	4.00	2.75
**B.King/1421**	81.00	77.75	-3.25	1.00	2.35	1.05	1.50	11.71	3.25	91.75	2.63	4.50	2.25	2.25
**ZM1421**	82.38	80.75	-1.63	1.00	2.10	0.95	1.50	10.71	2.25	88.00	2.63	2.00	1.75	3.25
**ZM1423**	70.25	71.88	1.63	1.00	2.17	0.94	1.00	13.71	1.25	144.25	4.63	2.50	1.75	2.50
**STR-SYN-Y2**	85.25	85.25	0.00	1.00	1.60	0.80	1.00	11.25	3.25	126.85	8.18	3.50	3.25	2.50
**DTSTR-W SYN13**	89.25	88.50	-0.75	1.00	0.98	0.75	1.50	10.00	3.75	115.35	4.68	5.50	3.50	2.50
**ZM1423/Z.DLO**	81.25	83.25	2.00	1.00	12.41	1.03	1.00	10.75	4.75	96.75	2.68	5.00	3.75	2.75
**DTSTR-Y SYN14**	80.13	79.75	-0.38	1.00	1.36	0.75	1.50	11.50	1.75	93.25	3.68	1.00	3.75	3.75
**DTSTR-Y SYN15**	83.75	84.25	0.50	1.00	1.78	0.65	1.00	9.00	6.25	87.35	3.18	4.50	4.00	2.75
**Trial statistics**														
**LSD (5%)**	5.71	7.10	2.58	0.09	1.58	1.15	0.62	2.18	1.39	34.87	34.87	2.37	1.62	1.17
**SEM**	8.19	15.88	5.08	0.18	2.58	2.26	1.39	4.87	4.20	77.95	77.95	5.31	3.62	2.61
**%CV**	7.15	8.95	597.49	12.24	87.15	28.59	49.30	21.39	43.80	52.81	52.81	64.59	47.58	41.79
**Heritability**	0.42	0.89	0.89	0.88	0.92	0.92	0.88	0.92	0.88	0.002	0.87	0.92	0.82	0.91

DA = days to 50% anthesis, DS = days to 50% silking, ASI = anthesis-silking interval, EPP = ear per plant, PLHT = plant height, EHT = ear height, HUSK = husk cover, CL = cob length, EASP = ear aspect, GY = grain yield, SEC8 = *Striga* emergence counts eight weeks after sowing, SEC10 = *Striga* emergence counts ten weeks after sowing, SDR8 = *Striga* damage rating eight weeks after sowing, and SDR10 = *Striga* damage rating 10 weeks after sowing. LSD = least significant difference, SEM = standard error of the mean, %CV = coefficient of variation, m = meter, cm = centimetre, g = gram.

In a *Sh*-infested environment, PLHT exhibited the highest coefficient of variation of 597.49% (Table 3). The grain yield of the inbred lines varied from 0.05 g/plant (HA04A-2107-36) to 151 g/plant (CML304), with a mean of 63.89 g/plant. A mean grain yield of 79.79 g/plant was recorded for the hybrids varying from 34.75 g/plant (Kep/1421) to 133.25 g/plant (N.Choice/1421), while the OPVs recorded an overall mean yield of 70.81 g/plant varying from 33.60 g/plant (TZBSTR) to 144.25 g/plant (ZM1423) (Table 3). Low broad-sense heritability values were computed for SEC10, SDR8, and SDR10 in *Sa-*infested conditions. In contrast, high heritability values were recorded for all the traits except for GY (H^2^ = 0.02) under *Sh*-infested conditions.

**Fig 1:**
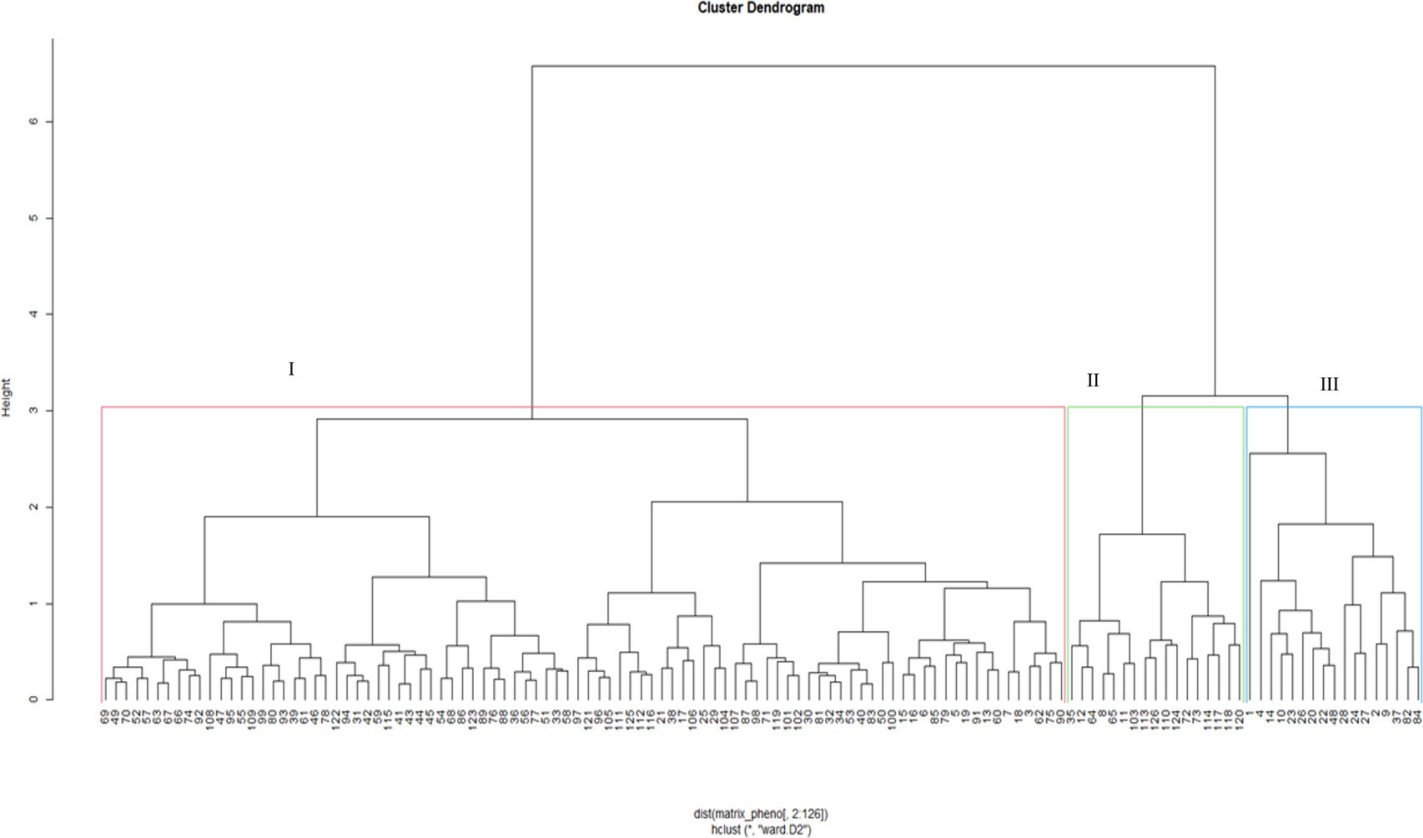
Dendrogram showing genetic relatedness among the 126 maize genotypes (G1 to G126) based on phenotypic traits under *Striga asiatica*-infested conditions. See Supplemental Table 2 in S1 File for the code of genotypes.

**Fig 2:**
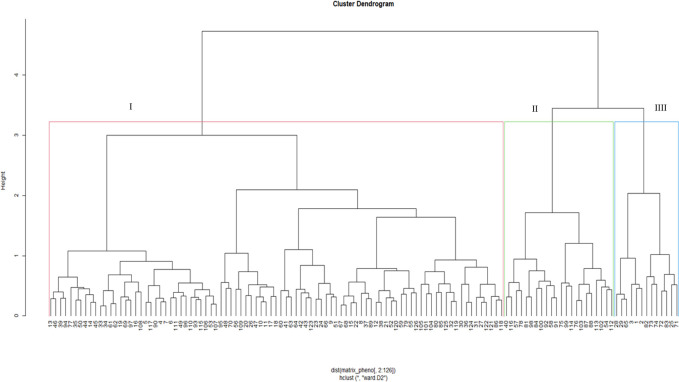
Dendrogram showing genetic relatedness among the 126 maize genotypes (G1 to G126) based on phenotypic traits under *Striga hermonthica*-infested conditions. See Supplemental Table 3 in S1 File for the code of genotypes.

Dendrograms based on phenotypic traits resolved the test genotypes into three clusters under *Sa* (Fig 1) and *Sh* (Fig 2) conditions. In a *Sa*-infested environment, Cluster I recorded the highest number of genotypes (91), followed by Cluster II (18), and Cluster III (17). Cluster I comprised tropical and sub-tropical genotypes from all sources. This Cluster had two sub-groups. The first sub-group is characterized by genotypes with low yield and moderate *Striga* resistance, whereas the second consists of genotypes with high yield and relatively high *Striga* resistance. Cluster II comprised 18 inbred lines mainly from IITA, while the genotypes in Cluster III were a mixture of *Striga*-resistant lines, drought-tolerant lines, and synthetic hybrids from IITA/Nigeria and CIMMYT/Zimbabwe. Under *Sh*-infested conditions, Cluster I was the largest (with 90 genotypes), followed by Cluster II (19) and Cluster III (17). Clusters I and II were composed of inbred lines from IITA and CIMMYT. Genotypes from Cluster III were from all sources; however, most were OPVs and hybrids sourced from IITA and NPGRC.

### 3.2 Genetic analysis using SNP markers

#### Genetic diversity and population structure

Table 4 summarizes the genetic diversity parameters of the biological types. The tested SNP markers were moderately polymorphic with a mean PIC value of 0.34 for the whole population, 0.33 for the inbred lines, 0.34 for the hybrids, and 0.35 for the OPVs. The whole population had a mean GD of 0.44. The OPVs exhibited the highest mean GD of 0.45 followed by the hybrids (0.44), and the inbred lines (0.42). The highest MAF was 0.37 observed among OPVs while the whole population exhibited an MAF of 0.36. The mean values of heterozygosity ranged from 0.22 to 0.28 with the highest Ho of 0.28 exhibited by the inbred lines. Overall, the level of fixation index ranged from 0.33 to 0.52. The OPVs exhibited the highest F of 0.52 followed by the hybrids (0.50).

**Table 4:** Genetic diversity parameters of 126 maize genotypes assessed based on 16000 SNP markers.

Whole population						Inbred lines					Hybrids					OPVs				
Diversity	GD	PIC	MAF	Ho	F	GD	PIC	MAF	Ho	F	GD	PIC	MAF	Ho	F	GD	PIC	MAF	Ho	F
Lower	0.19	0.17	0.10	0.08	0.13	0.11	0.11	0.06	0.09	0.08	0.00	0.00	0.00	0.08	0.27	0.08	0.08	0.04	0.08	0.17
Upper	0.50	0.38	0.50	0.38	0.81	0.50	0.38	0.50	0.38	0.78	0.50	0.38	0.50	0.33	0.81	0.50	0.38	0.50	0.37	0.81
Mean	0.44	0.34	0.36	0.26	0.41	0.42	0.33	0.33	0.28	0.33	0.44	0.34	0.36	0.22	0.50	0.45	0.35	0.37	0.22	0.52

GD = gene diversity, PIC = polymorphism information content, MAF = minor allele frequency, Ho = observed heterozygosity, F = fixation index.

**Fig 3:**
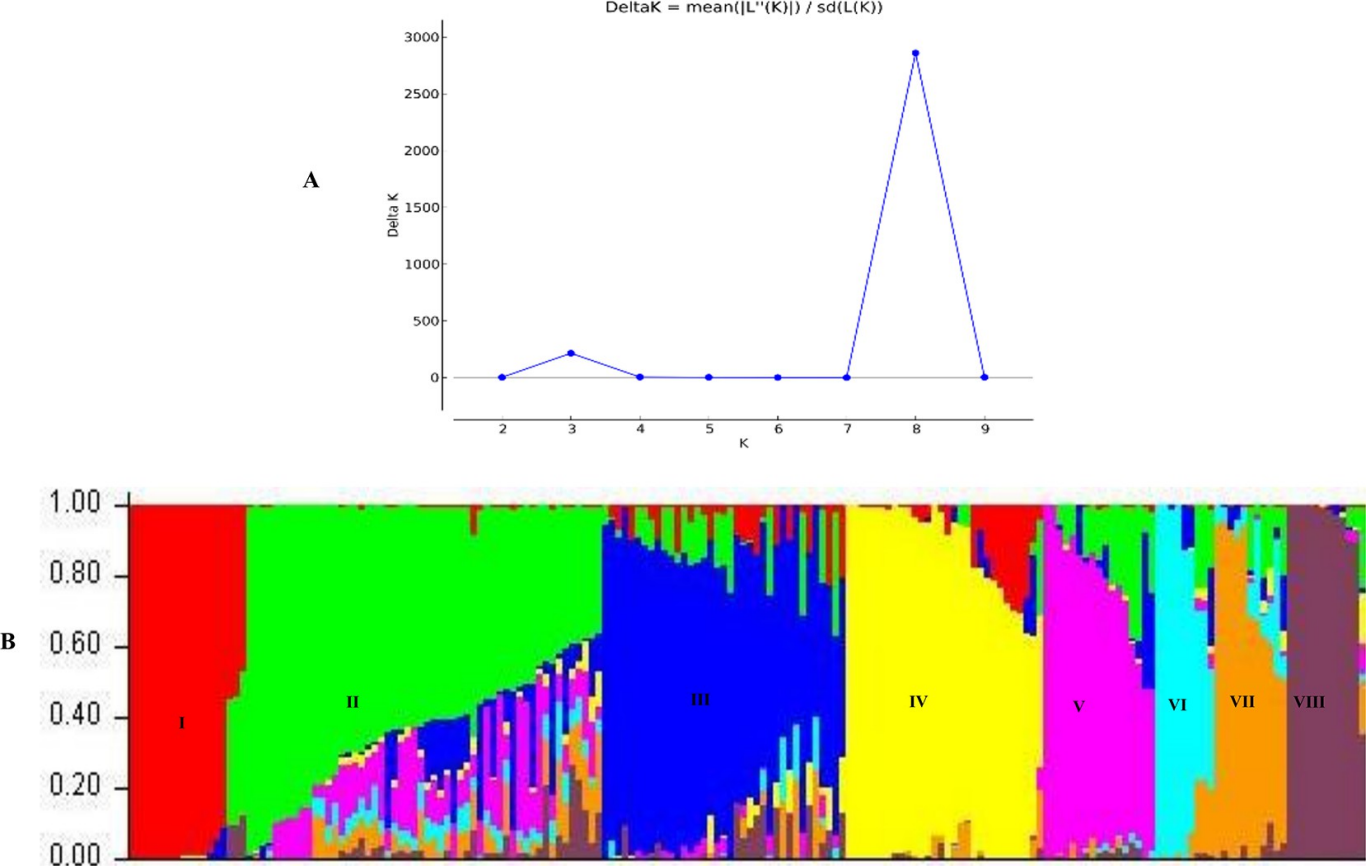
Sub-population inference among the 126 maize genotypes based on 16000 SNPs: (A) likelihood and delta K values for different numbers of assumed clusters and (B) population structure among the 126 maize genotypes based on 16000 SNPs at K = 8.

**Fig 4:**
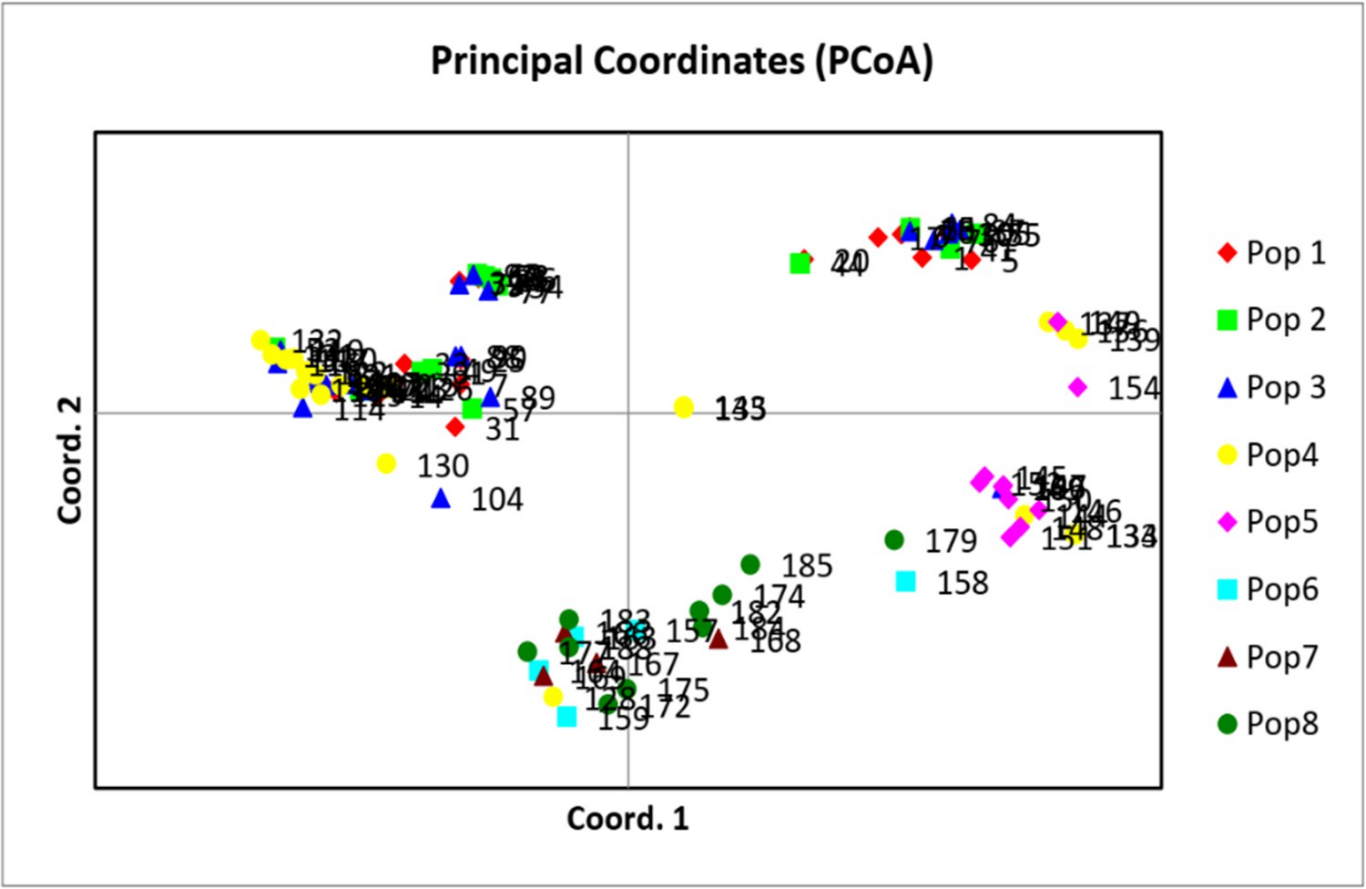
Principal coordinate analysis clustering of the test genotypes. See Supplemental Table 3 in S1 File for the code of genotypes.

The structure analysis based on the Evanno method indicated that the highest value of ΔK was eight (Fig 3A), revealing eight main genetic clusters (Fig 3B). About 55.31% of the tested genotypes exhibited membership coefficient values ≥ 0.70. The rest, accounting for 44.69%, were considered admixtures. Sub-population II was the largest group, with 22 accessions (21.15%) representing OPVs and synthetic hybrids from IITA/Nigeria, CIMMYT/Zimbabwe, and NPGRC/South Africa. Sub-population III comprised 21 accessions (20.19%), comprising IITA/Nigeria inbred lines and hybrids. Sub-population IV composed of 19 accessions (18.26%) that were IITA hybrids and some IITA inbred lines. About 14 accessions (13.46%) were allocated to the sub-population I comprising CIMMYT/Zimbabwe inbred lines. Sub-population V constituted 10 accessions (9.61%) that were CIMMYT/Zimbabwe inbred lines. Sub-populationsVI, VII, and VIII comprised ten, five, and four accessions, respectively. Members of these populations were inbred lines from IITA/Nigeria. Principal coordinate analysis assigned the accessions to four admixture groups (Fig 4). In particular, sub-populations I and II were clustered in PC1, while sub-population V was dominant in PC2. Sub-populations VI, VII, and VIII were clustered in PC3, whereas sub-populations III and IV were dominants in PC4.

#### Genetic distance

The inbreeding coefficient ranged from -0.06 to 0.59, with a mean of 0.34 representing the population pairs VI and VIII, and V and VII (Table 5, bottom diagonal). The pairwise genetic distance among the eight populations ranged from 0.16 to 0.48, with a mean of 0.32 (Table 5, upper diagonal). Sub-populations III and VIII, and IV and III were the most distantly related, while sub-population VII had relatively the shortest distances from sub-populations II and VI. It was noticed that the genetic distances between the sub-populations III, IV, V, VI, and VII are beyong the average. The same extent was noticed with sub-populations I, V, VI, VII, and VIII. The sub-population III consists of the genotype NC.QPM/Z.DPLO, Shesha/1421, and NC.QPM/Z.DPLO and was associated with high GY under *Sa*-infested conditions. Sub-population VIII and IV consisted of IITA inbred lines including TZISTR1175, TZISTR1225, TZISTR1190, TZISTR1174, and TZISTR1166 that were associated with high SDR8 and SDR10 reduction under *Sa* infested-conditions, and TZISTR1205 and TZSTRI108 associated with high GY under *Sh*-infested conditions. Nei’s genetic distance between the individuals based on the 16000 SNP markers ranged from 0.01 to 0.34 within the inbred lines with a mean of 0.18 (Supplemental Table 4 in S1 File and Table 5). TZISTR1008 and CLHP0221 had the lowest genetic distance of 0.01, while CLHP0343 and TZISTR1223 exhibited the highest genetic distance of 0.34. CLHP0343 was associated with good GY under *Sa* infestation and exhibited a relatively high genetic distance from all the other inbred lines. The accessions CML540, TZISTR25, TZISTR1248, CLHP0303, TZISTR1174, TZSTRI113, TZDEEI50, TZSTRI115, CML539, TZISTR1015, CZL99017, CML451, CML566, CLHP0343 and CML440 which showed high GY and reduce *Striga* damage under both *Sa* and *Sh* infested conditions, exhibited high and average genetic distances from each other.

**Table 5:** Genetic distance (upper diagonal), and pairwise inbreeding coefficients (lower diagonal), among eight populations resulting from 130 maize genotypes based on 16000 SNP profiling.

Populations	Fst (genetic distance)							
	C1	C2	C3	C4	C5	C6	C7	C8
**C1**	-	0.30	0.29	0.25	0.42	0.39	0.34	0.42
**C2**	-0.04	-	0.29	0.26	0.20	0.19	0.16	0.29
**C3**	0.00	0.00	-	0.44	0.42	0.38	0.34	0.48
**C4**	0.13	0.14	0.04	-	0.35	0.31	0.26	0.34
**C5**	0.46	0.50	0.45	0.33	-	0.31	0.28	0.36
**C6**	0.54	0.55	0.49	0.39	0.54	-	0.18	0.35
**C7**	0.57	0.58	0.51	0.39	0.59	-0.02	-	0.30
**C8**	0.52	0.52	0.48	0.39	0.50	-0.06	0.04	-
	**Fis (inbreeding coefficient)**							

C1 to C8 represent the clusters generated by the Structure analysis.

The analysis of molecular variance (AMOVA) showed a significant variation within populations (Table 6). The within-population variation accounted for 85% of the total variation. The variation detected among the population was low (15%).

**Table 6:** Analysis of molecular variance involving 130 maize accessions based on 16000 SNP markers.

Source	df	SS	MS	Estimated Variance	Proportion of variance
Among Populations	7	88048.86	12578.41	696.07	0.15
Within Populations	96	369493.72	3848.89	3848.89	0.85
Total	103	457542.59		4544.96	1.00

Df, degrees of freedom; SS, sum of squares; MS, mean squares

**Fig 5:**
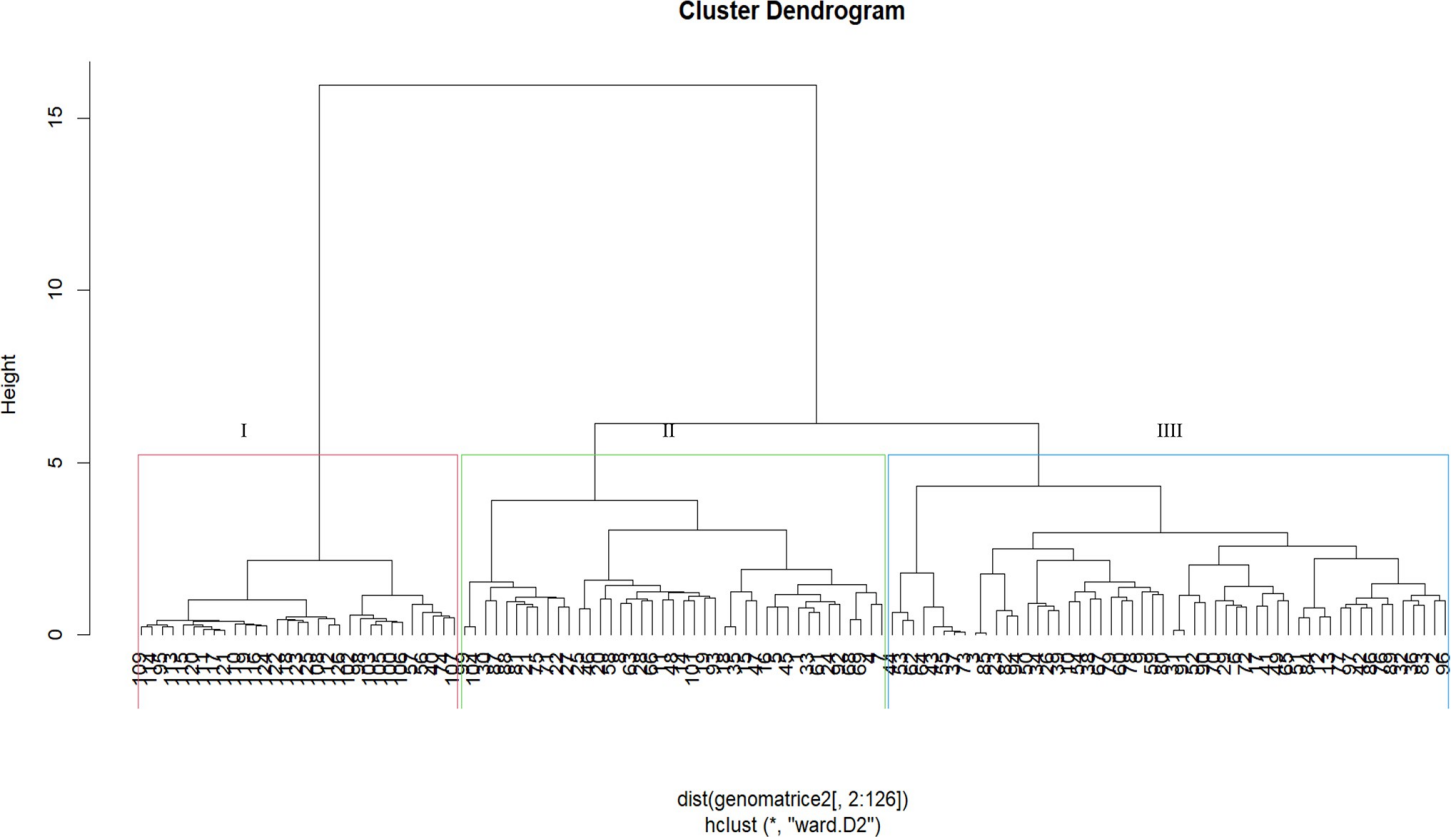
Hierarchical cluster dendrogram showing the genetic relationships among 126 maize accessions using 16000 SNP markers. See Supplemental Table 3 in S1 File for the code of genotypes.

The dendrogram based on the 16000 SNP markers clustered the accessions into three major clusters (Fig 5). The largest is Cluster III, containing mainly CIMMYT and IITA inbred lines, followed by Cluster II, consisting of admixtures of IITA and CIMMYT lines and synthetic hybrids. Cluster I form genotypes from all sources, mainly OPVs from IITA.

#### Comparison of test genotypes using phenotypic and genotypic analyses

**Fig 6:**
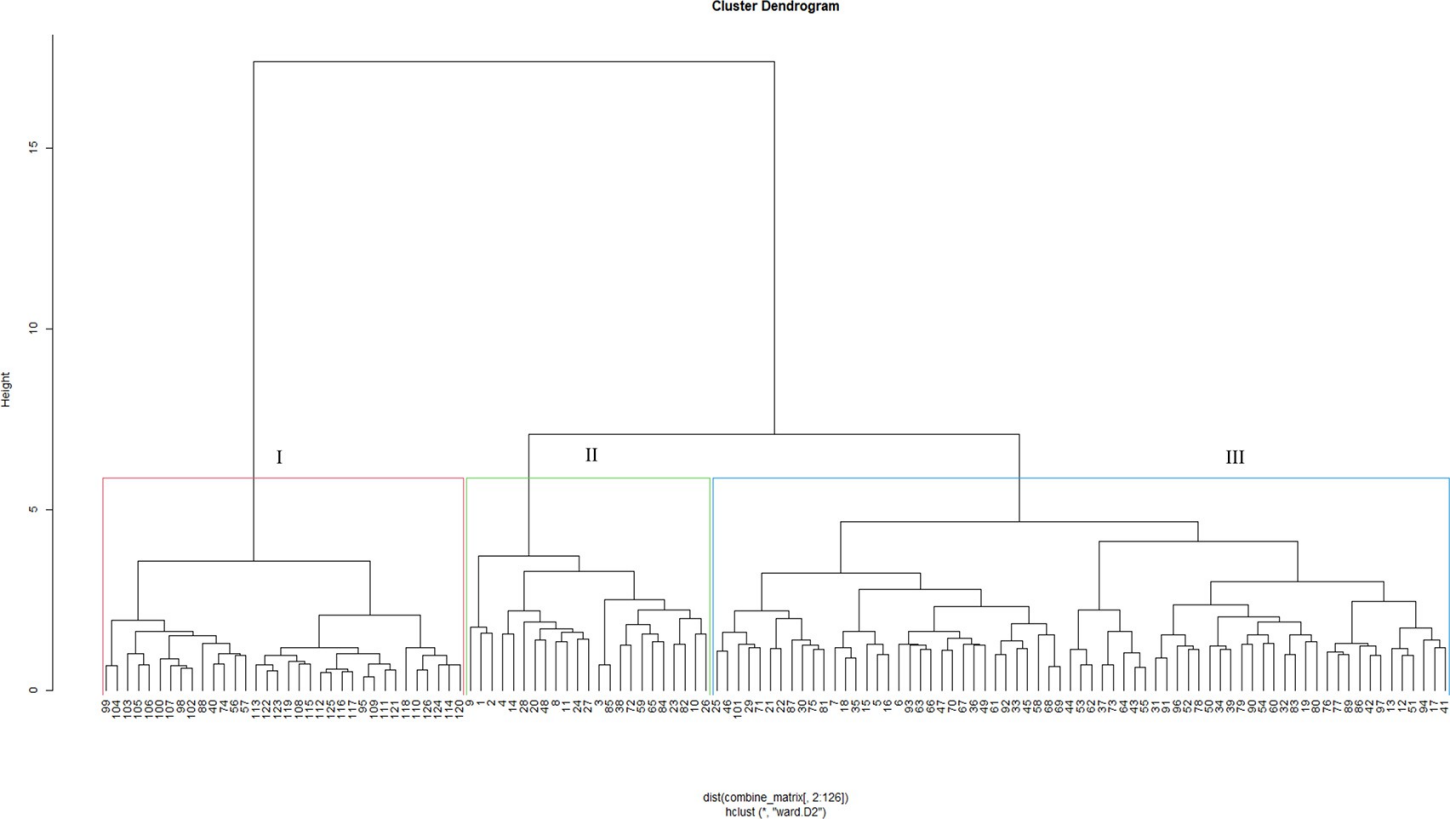
Dendrogram showing relatedness among the 126 maize genotypes under *Striga asiatica*-infested conditions using genotypic and phenotypic data. See Supplemental Table 4 in S1 File for the code of genotypes.

**Fig 7:**
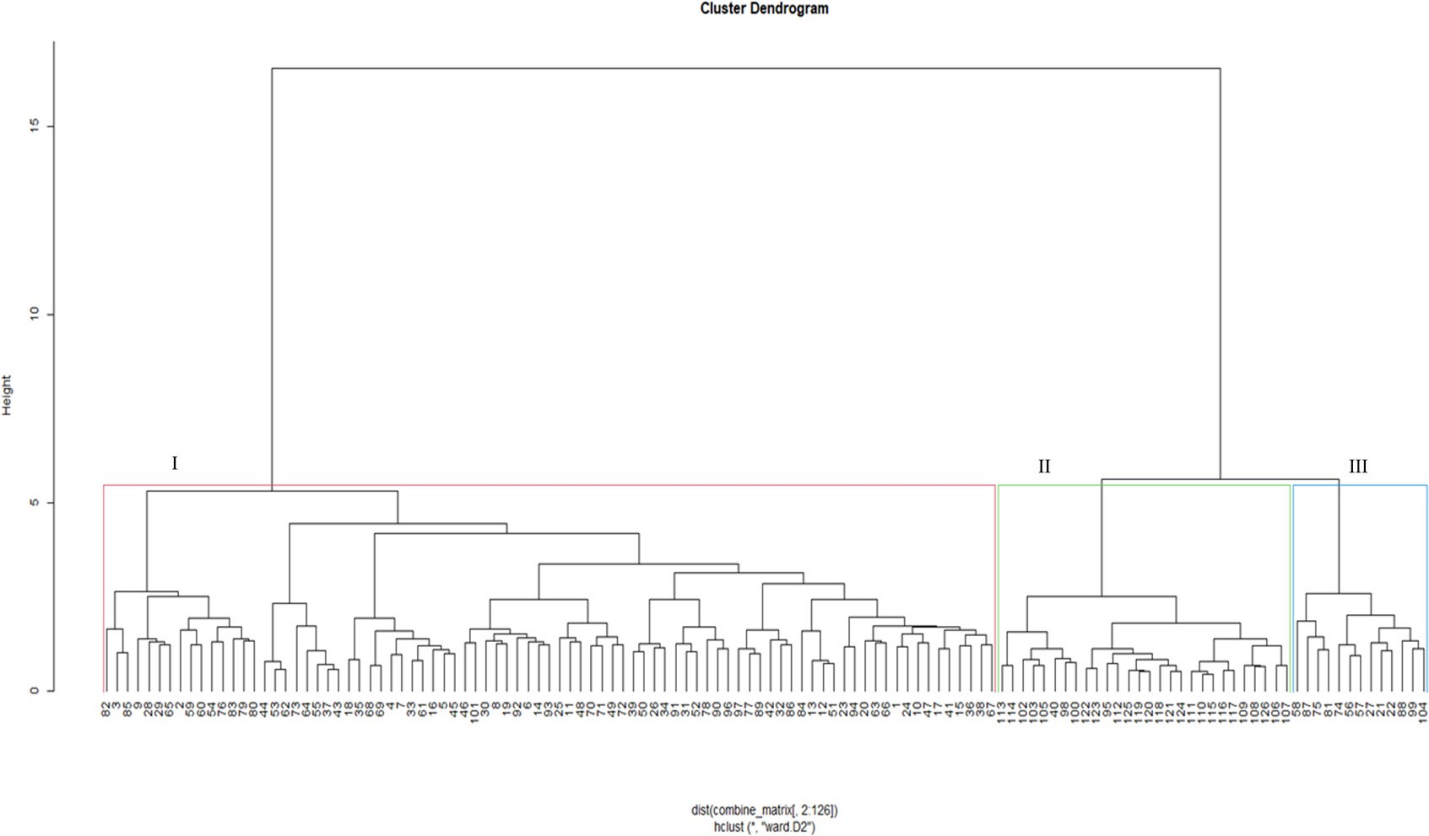
Dendrogram showing relatedness among the 126 maize genotypes under *Striga hermonthica* conditions using genotypic and phenotypic data. See Supplemental Table 4 in S1 File for the code of genotypes.

Figs 6 and 7 present the joint analysis that revealed three clusters for both tested conditions using the phenotypic and molecular data. Under *Sa* conditions, Cluster III was the largest, with 68 genotypes, followed by Cluster I (35), and Cluster II (23). Under *Sh* conditions, Cluster I was the largest, followed by Clusters II and III with 84, 28, and 14 genotypes, respectively.

**Fig 8:**
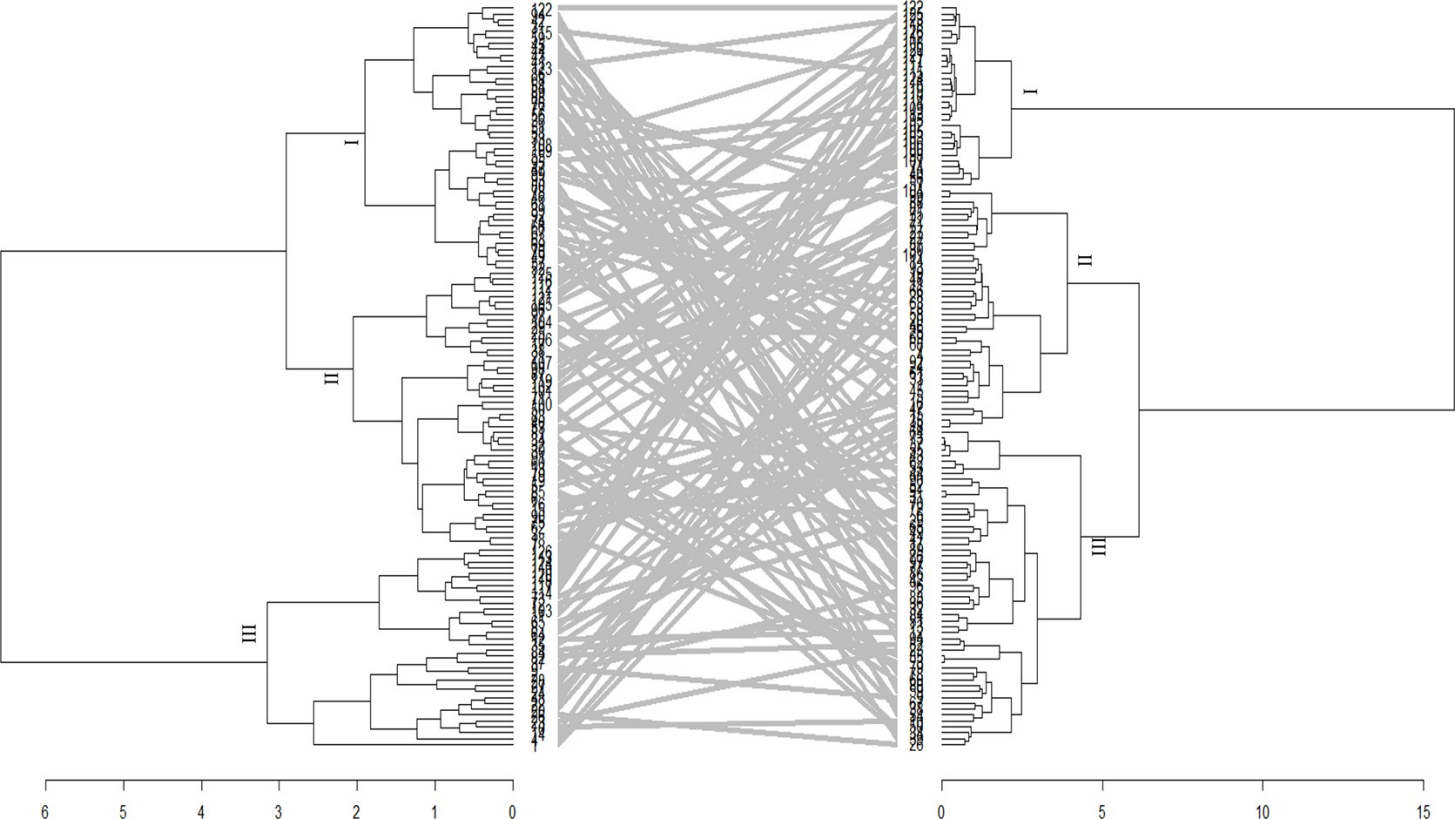
Tanglegram comparing dendrograms based on evaluation of 126 maize genotypes evaluated using phenotypic (left) and genotypic data (right) under *Striga asiatica* conditions. See Supplemental Table 4 in S1 File for the code of genotypes.

**Fig 9:**
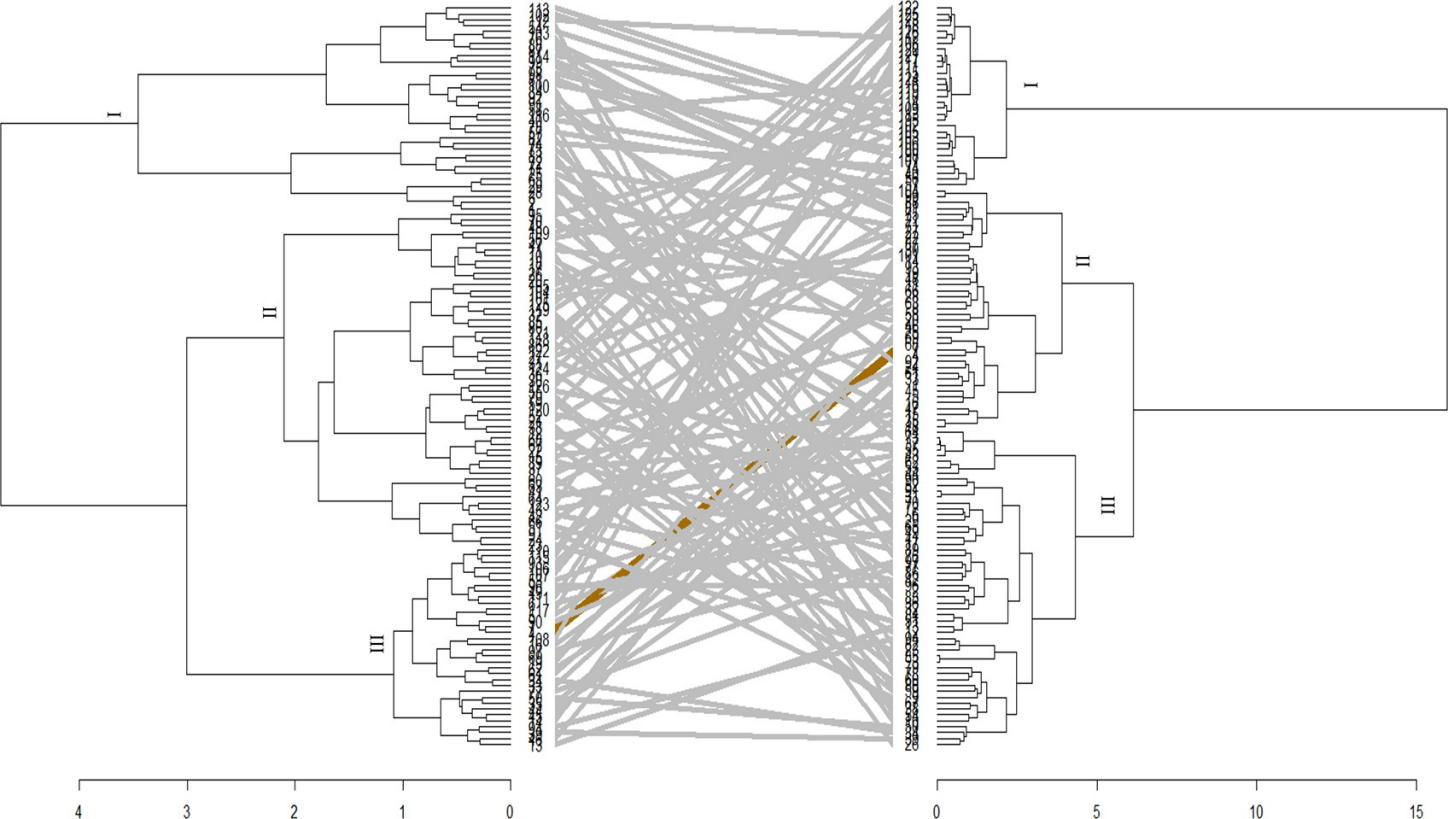
Tanglegram comparing dendrograms based on evaluation of 126 maize genotypes evaluated using phenotypic (left) and genotypic data (right) under *Striga hermonthica* conditions. See Supplemental Table 4 in S1 File for the code of genotypes.

The phylogenetic tree generated from the phenotypic data was compared to the genotype grouping based on the SNPs data (Figs 8 and 9). Only a few genotypes (21.42%) maintained their positions across the hierarchical clusters. Furthermore, the correlation between the phenotypic and genotypic dissimilarity matrices was low according to the Mantel test in *Sh* (r^2^ = 0.0009, P = 0.01) and *Sa* (r^2^ = 0.0006, P = 0.02) infested environments.

## 4. Discussion

Genetic variation is fundamental for new or pipeline crop breeding programs. The development of open-pollinated, hybrid and synthetic maize varieties with high hybrid vigour relies on genetically contrasting parents and heterotic groups emanating from well-characterized genetic resources. The present study assessed the genetic diversity of 126 maize genotypes (Supplemental Table 1 in S1 File) sourced from IITA/Nigeria, CIMMYT/Zimbabwe, and NPGRC/South Africa using agro-morphological traits and high-density SNP markers. Morphological traits are useful in preliminary genetic diversity assessments [44] and ideotype breeding [45, 46].

In the current study, a wide variability was recorded among accessions of different sources using phenotypic traits (Table 1). Each source of genotype group presented a unique selection with specific and unique traits (Tables 2 and 3). For instance, genotypes CML540 and CML566 were higher yielders in *Sa*-infested environment (Table 2), while genotypes CML304 and TZSTRI101 were higher yielders in *Sh*-infested environment (Table 3). These genotypes are ideal candidates for *Striga* resistance breeding. Some of the tropical genotypes bred for *Sh* resistance were susceptible to *Sa*. This is consistent with the previous finding of Gasura, *et al*. [47], who reported the susceptibility to *Sa* of some tropical inbred lines bred for *Sh* resistance. Low broad sense heritability values were computed for SEC10, SDR8, and SDR10 in *Sa-*infested environment (Table 2), indicating that *Sa*-resistance has low heritability, and therefore, the phenotype was a poor measure of the genetic merit of the evaluated genotypes, which reduces the effectiveness of selection under *Sa* infestation. These findings differ from those of Olakojo and Olaoye [48], who reported a high heritability of *Striga* syndrome rating and *Striga* emergence count under *Sa*-infested conditions. Meanwhile, high heritability values were recorded for the same traits under *Sh*-infested conditions (Table 3). This suggests that, unlike *Sa* resistance, *Sh* resistance is highly heritable. This shows that the results would be repeatable, which is ideal for *Sh* resistance breeding. Kaewchumnong and Price [49] and Stanley, et al. [30] reported high heritability estimates for *Striga* resistance traits in a *Sh*-infested environment. This finding, however differs from those of Badu-Apraku, *et al*. [50], who recorded low heritability estimates for emerged *Striga* plants and *Striga* damage ratings. All these results suggest that the gene actions controlling *Sa* and *Sh* are not the same.

Based on phenotypic traits, the dendrogram delineated the genotypes into three major clusters subdivided into six sub-clusters under *Sa*-infested conditions (Fig 1), and four under *Sh*-conditions (Fig 2). The clusters were formed based on reaction to *Sa* and *Sh* infestations and yield components performances. This suggests the presence of considerable genetic variation among the assessed genotypes that could be used in developing *Striga-*resistance germplasm. Reports on the clustering of genotypes based on phenotypic traits are common in genetic studies in maize [51, 52].

Compared with morphological traits, molecular markers are independent of environmental effects and can provide additional and accurate information for assessing genetic diversity [53, 54]. This study used SNP markers to assess the genetic diversity of tropical and sub-tropical maize germplasm. The test germplasm exhibited a high heterozygosity of 0.26 (Table 4), suggesting that alternative alleles were represented in the population. The inbred lines exhibited the highest heterozygosity estimates. The observed heterozygosity in the inbred lines (28%) exceeded the expectations (6.25%) for inbred lines derived after four generations of selfing needing continuous selfing, given that the inbred lines are relatively in the early generation of inbreeding [55]. The PIC and GD values were useful to assess the population’s genetic diversity to identify divergent parental lines for breeding programs. The mean PIC and GD values were 0.34 and 0.44, respectively, for the whole population, and the same trend was observed for the inbred lines, the hybrid checks, and the OPV checks (Table 4). This shows that the 16000 SNP markers in this study were polymorphic to distinguish the test population, inbred lines, and checks. The PIC value corresponds to the ability of the test markers to detect the polymorphism among individuals of the population [56]. The PIC values in this study are higher compared to PIC values reported in some of the past related studies. Adu, et al. [17] reported PIC values within the range of 0.01 to 0.38 using 15,047 SNP markers on 94 maize inbred lines. Badu-Apraku, et al. [19] reported PIC values ranging from 0.029 to 0.37 with a mean of 0.21 using 9642 SNP markers. The mean PIC values observed in this study are comparable to Yang, *et al*. [57]. The mean GD of the population in this study (0.44) was similar to the one reported by Eschholz, *et al*. [58] when using SSR markers. Yacoubou, et al. [26] reported a gene diversity value of 0.44 in early-generation maize lines. According to the formula of Anderson, *et al*. [59], the theoretical maximum gene diversity for bi-allelic markers is 0.50. This signifies that the gene diversity obtained in this study was high, suggesting a significant genetic segregation in the test population in this study. Genetic diversity reflects the population’s genetic constitution and its adaptability in various environments [60].

The genetic differentiation recorded in this study ranged from 0.16 to 0.48 (Table 5). According to Wright [61] an Fst of 0–0.005 indicates low, 0.05–0.15 moderate, 0.15–0.25 high, and above 0.25 very significant genetic differentiations. The Fst value in the present study is indicative of high genetic differentiation among the heterotic groups, which was expected. This result is confirmed by the high rate of inbreeding coefficient, reflecting a low level of genetic identity for the populations in this study. Genetic differentiation occurs when there is restricted gene flow between populations [62]. The high genetic differentiation observed in this study agrees with previous studies in maize [63, 64].

The analysis of molecular variance is a suitable criterion for assessing the overall diversity distribution within and among populations. The AMOVA results in this study showed a higher level of genetic variation within populations than among populations of the test genotypes (Table 6), which supports the high genetic differentiation. Related findings were reported by Leng, *et al*. [65] and Mathiang, *et al*. [66]. Based on phenotyping, the test genotypes were resolved into six clusters under *Sa*-infested (Fig 1) conditions and four clusters under *Sh*-infested conditions (Fig 2). The model-based population structure analysis (Fig 3), principal coordinate analysis (Fig 4), and neighbour-joining cluster analysis (Fig 5) revealed the presence of eight groups, which is fairly consistent with pedigree information and with putative heterotic groups. This is supported by the very low and significant correlation exhibited by the phenotypic and genotypic distance matrices, revealing the discordance between the two matrices. The discordance between phenotypic and genotypic matrices is partially attributed to the environment effect on the phenotypic trait’s expression [21]. Other studies reported inconsistency between phenotypic and genotypic matrices [54, 67].

## Conclusion

The results of the present study revealed significant phenotypic and molecular diversity of the tropical and sub-tropical maize populations. Significant differences were recorded for all the assessed quantitative traits. The SNPs used in this study revealed the genetic variation among the test population. The mean gene diversity and polymorphic information content were 0.34 and 0.44, respectively, reflecting a moderate level of genetic variation among the test genotypes when assessed using SNP markers. The overall mean genetic distance among the inbred lines was 0.18, ranging from 0.01 to 0.34. Divergent parents were selected for hybridization and the development of new *Striga*-resistant varieties in SSA. The following genetically distant genotypes were selected, displaying good agronomic performance and *Sa* and *Sh* resistance: CML540, TZISTR25, TZISTR1248, CLHP0303, TZISTR1174, TZSTRI113, TZDEEI50, TZSTRI115, CML539, TZISTR1015, CZL99017, CML451, CML566, CLHP0343 and CML440. Genetically diverse and complementary lines were selected among the tropical and sub-tropical maize populations that will facilitate the breeding of maize varieties with *Striga* resistance and market-preferred traits. Both molecular and morphological features are useful and will facilitate the selection and breeding process for *Striga* resistance in maize.

## Supporting information

S1 Table Raw phenotypic collected under *Striga asiatica*. (XLSX)

S2 Table Raw phenotypic data collected under *Striga hermonthica*. (XLSX)

S3 Table SNP data. (TXT)

S4 Table *S*. *asiatica* data normalized. (DOCX)

S5 Table *S*. *hermonthica* data normalised. (DOCX)

S1 File (ZIP)
