# Supplementary material for: Genetic diversity analysis of tropical and sub-tropical maize germplasm for Striga resistance and agronomic traits with SNP markers
Source: PLoS One. 2024 Aug 6;19(8):e0306263. doi: 10.1371/journal.pone.0306263 (PMC11302897; doi:10.1371/journal.pone.0306263)
Supplement: S4 Table — (DOCX) [file pone.0306263.s004.docx]

**Supporting information:** Mean responses for 14 maize and *Striga* parameters assessed from 126 maize genotypes evaluated under *Striga asiatica* infestation with anthesis silking interval normalized.

| **N°** | **Accessions** | **DA** | **DS** | **ASI** | **EPP** | **PLHT** | **EHT** | **HUSK** | **CL** | **EASP** | **GY** | **SEC8** | **SEC10** | **SDR8** | **SDR10** |
| --- | --- | --- | --- | --- | --- | --- | --- | --- | --- | --- | --- | --- | --- | --- | --- |
|  |  |  |  |  |  | **(m)** | **(m)** | **(1 to 5)** | **(cm)** | **1 to 9)** | **g/plant)** |  |  | **(1 to 9)** | **(1 to 9)** |
| 1 | TZISTR1154 | 85.50 | 83.00 | 14.00 | 2.00 | 1.52 | 1.53 | 1.00 | 10.75 | 6.50 | 47.50 | 2.00 | 5.00 | 1.00 | 1.50 |
| 2 | TZISTR1261 | 87.50 | 82.00 | 11.00 | 2.00 | 1.73 | 0.85 | 1.00 | 13.75 | 4.50 | 51.25 | 2.50 | 2.00 | 2.00 | 2.00 |
| **3** | **TZISTR1248** | **78.00** | **73.50** | 12.00 | **1.00** | **1.30** | **0.59** | **1.00** | **13.25** | **3.00** | **98.25** | **3.50** | 17.50 | **4.50** | **2.00** |
| 4 | TZISTR1263 | 87.50 | 82.00 | 11.00 | 1.00 | 1.40 | 0.53 | 3.00 | 10.50 | 3.50 | 52.50 | 2.00 | 5.50 | 5.00 | 1.00 |
| 5 | TZISTR1275 | 89.50 | 76.00 | 3.00 | 1.00 | 1.60 | 0.75 | 2.00 | 14.75 | 6.00 | 54.50 | 5.00 | 5.00 | 3.00 | 2.50 |
| 6 | TZISTR1157 | 82.00 | 77.50 | 12.00 | 1.00 | 1.59 | 0.62 | 2.00 | 10.75 | 3.00 | 44.50 | 4.50 | 11.50 | 2.50 | 2.50 |
| 7 | TZISTR1160 | 84.50 | 83.50 | 15.50 | 1.00 | 1.00 | 0.60 | 1.50 | 12.50 | 7.00 | 35.00 | 5.00 | 14.50 | 3.50 | 2.00 |
| 8 | TZISTR1162 | 80.50 | 78.00 | 14.00 | 1.00 | 1.54 | 1.13 | 1.00 | 13.50 | 2.00 | 57.50 | 4.50 | 2.50 | 1.50 | 2.00 |
| 9 | TZISTR1165 | 79.50 | 75.50 | 12.50 | 2.00 | 1.85 | 1.15 | 1.00 | 11.50 | 5.00 | 32.50 | 5.00 | 16.50 | 1.50 | 1.50 |
| 10 | TZISTR1175 | 76.00 | 72.50 | 13.00 | 1.00 | 1.58 | 0.65 | 1.00 | 14.50 | 6.50 | 55.00 | 5.50 | 28.00 | 1.50 | 1.50 |
| 11 | TZISTR1178 | 76.00 | 74.50 | 15.00 | 1.00 | 2.00 | 1.55 | 1.00 | 10.15 | 1.00 | 64.55 | 5.00 | 3.50 | 3.00 | 1.50 |
| 12 | TZISTR1163 | 82.00 | 78.00 | 12.50 | 1.00 | 1.70 | 1.01 | 1.00 | 9.25 | 1.00 | 57.25 | 5.00 | 5.00 | 3.50 | 2.00 |
| 13 | TZISTR1166 | 99.00 | 101.00 | 18.50 | 1.00 | 1.00 | 0.78 | 1.00 | 10.50 | 4.00 | 62.50 | 5.50 | 7.50 | 3.00 | 2.00 |
| 14 | TZISTR1190 | 86.00 | 84.00 | 14.50 | 1.00 | 1.75 | 0.88 | 1.00 | 8.25 | 7.00 | 31.00 | 4.50 | 12.00 | 3.50 | 1.75 |
| 15 | TZISTR1199 | 81.00 | 78.50 | 14.00 | 1.00 | 1.85 | 0.90 | 1.00 | 7.75 | 4.50 | 36.00 | 5.50 | 5.00 | 3.00 | 3.00 |
| 16 | TZISTR1231 | 75.00 | 73.00 | 14.50 | 1.00 | 1.55 | 0.90 | 1.00 | 10.05 | 4.50 | 62.50 | 5.00 | 8.00 | 3.00 | 2.50 |
| 17 | TZISTR1232 | 85.50 | 86.00 | 17.00 | 1.00 | 1.75 | 1.04 | 1.00 | 11.50 | 5.00 | 35.00 | 5.50 | 15.50 | 2.50 | 2.00 |
| 18 | TZISTR1259 | 77.00 | 74.00 | 13.50 | 1.00 | 1.40 | 0.76 | 1.00 | 15.50 | 5.50 | 43.25 | 5.00 | 10.00 | 4.50 | 1.50 |
| 19 | TZISTR1262 | 83.50 | 74.50 | 7.50 | 1.00 | 1.65 | 0.82 | 2.00 | 11.50 | 6.50 | 0.00 | 4.50 | 16.50 | 3.50 | 2.00 |
| 20 | TZISTR1159 | 86.50 | 71.00 | 1.00 | 1.00 | 1.90 | 0.97 | 1.00 | 9.75 | 4.50 | 29.25 | 5.50 | 8.00 | 1.50 | 1.50 |
| 21 | TZISTR1223 | 87.00 | 83.50 | 13.00 | 1.00 | 1.95 | 1.30 | 1.00 | 9.50 | 3.50 | 60.00 | 4.50 | 8.00 | 2.00 | 1.50 |
| 22 | TZISTR1225 | 85.50 | 84.50 | 15.50 | 1.00 | 1.90 | 1.45 | 1.00 | 10.50 | 3.00 | 62.50 | 4.50 | 4.50 | 1.00 | 2.00 |
| 23 | CML550 | 85.50 | 85.00 | 16.00 | 1.00 | 2.03 | 1.15 | 1.00 | 12.50 | 6.50 | 28.75 | 4.50 | 11.50 | 3.50 | 3.50 |
| 24 | TZISTR1244 | 76.00 | 76.50 | 17.00 | 1.00 | 1.00 | 0.45 | 2.00 | 9.00 | 3.50 | 47.50 | 5.00 | 7.50 | 3.00 | 3.50 |
| 25 | TZSTRI101 | 99.00 | 98.50 | 16.00 | 1.00 | 1.00 | 0.55 | 1.00 | 9.00 | 3.50 | 52.50 | 5.00 | 14.00 | 3.00 | 3.50 |
| 26 | TZSTRI102 | 87.50 | 78.00 | 7.00 | 1.00 | 1.85 | 0.85 | 1.00 | 10.50 | 1.50 | 52.25 | 5.00 | 15.50 | 4.00 | 2.50 |
| 27 | TZSTRI104 | 100.00 | 99.00 | 15.50 | 1.00 | 1.00 | 0.45 | 1.50 | 10.50 | 3.50 | 57.50 | 5.00 | 23.00 | 3.00 | 4.00 |
| 28 | TZSTRI107 | 77.50 | 73.50 | 12.50 | 1.00 | 1.40 | 0.84 | 1.00 | 8.50 | 2.00 | 57.00 | 5.00 | 1.50 | 1.50 | 2.50 |
| 29 | TZSTRI108 | 81.00 | 80.00 | 15.50 | 1.00 | 1.40 | 0.77 | 2.00 | 9.00 | 2.00 | 28.00 | 5.00 | 4.50 | 5.50 | 3.00 |
| 30 | TZSTRI109 | 80.00 | 73.00 | 9.50 | 2.00 | 2.03 | 0.97 | 1.00 | 11.00 | 5.00 | 57.25 | 4.50 | 2.50 | 2.50 | 2.00 |
| 31 | TZSTRI110 | 82.00 | 74.50 | 9.00 | 1.00 | 2.03 | 1.45 | 1.00 | 7.75 | 5.00 | 37.00 | 4.50 | 29.50 | 2.00 | 2.50 |
| 32 | TZSTRI112 | 81.00 | 76.50 | 12.00 | 1.00 | 1.08 | 0.80 | 1.00 | 12.50 | 5.00 | 68.75 | 4.50 | 4.50 | 3.00 | 3.50 |
| 33 | TZSTRI114 | 93.00 | 93.50 | 17.00 | 1.00 | 1.05 | 0.55 | 1.00 | 10.50 | 4.50 | 65.00 | 5.00 | 19.00 | 3.00 | 3.50 |
| 34 | **TZSTRI115** | **77.50** | **77.00** | 16.00 | **1.00** | **2.10** | **1.20** | **1.00** | **11.50** | **1.50** | **112.50** | **5.00** | 2.00 | **3.50** | **2.50** |
| 35 | **TZISTR25** | **76.50** | **72.50** | 12.50 | **1.00** | **2.25** | **1.13** | **1.00** | **13.50** | **2.00** | **97.25** | **5.00** | 4.50 | **3.00** | **3.50** |
| 36 | **TZISTR1001** | **82.00** | **82.00** | 16.50 | **1.00** | **2.10** | **1.28** | **1.00** | **11.00** | **1.50** | **140.00** | **4.50** | 4.50 | **3.00** | **2.50** |
| 37 | TZISTR1003 | 84.50 | 80.00 | 12.00 | 1.00 | 2.05 | 1.42 | 1.00 | 8.75 | 2.00 | 44.50 | 4.50 | 32.00 | 3.00 | 3.50 |
| 38 | TZISTR1004 | 70.50 | 72.50 | 18.50 | 1.00 | 1.58 | 1.13 | 1.00 | 9.25 | 1.50 | 54.50 | 4.50 | 4.50 | 2.00 | 4.00 |
| 39 | TZISTR1008 | 85.50 | 83.50 | 14.50 | 1.00 | 2.00 | 1.13 | 1.00 | 11.00 | 5.50 | 38.75 | 5.00 | 9.50 | 4.50 | 3.00 |
| 40 | TZISTR1011 | 91.00 | 91.50 | 17.00 | 1.00 | 1.80 | 1.15 | 1.00 | 10.75 | 2.50 | 58.00 | 4.50 | 12.50 | 4.50 | 3.50 |
| 41 | TZISTR1018 | 75.50 | 74.50 | 15.50 | 1.00 | 1.70 | 1.15 | 1.00 | 12.00 | 3.50 | 47.75 | 5.00 | 8.00 | 1.00 | 1.00 |
| 42 | TZEEI21 | 81.50 | 82.00 | 17.00 | 1.00 | 1.18 | 0.80 | 1.00 | 12.50 | 3.00 | 67.50 | 4.50 | 2.50 | 4.50 | 3.50 |
| 43 | TZEEI13 | 77.00 | 76.00 | 15.50 | 1.00 | 1.30 | 0.72 | 2.00 | 8.50 | 3.00 | 58.25 | 5.50 | 5.50 | 3.50 | 5.50 |
| 44 | TZEEI14 | 82.00 | 82.00 | 16.50 | 1.00 | 2.10 | 1.13 | 2.00 | 10.25 | 1.50 | 63.00 | 4.50 | 9.50 | 4.50 | 3.00 |
| 45 | TZEEI49 | 72.50 | 74.50 | 18.50 | 1.00 | 1.97 | 0.77 | 2.00 | 11.25 | 3.00 | 36.00 | 4.50 | 7.00 | 5.00 | 5.00 |
| 46 | TZDEEI55 | 76.50 | 75.50 | 15.50 | 1.00 | 1.59 | 0.61 | 2.00 | 8.50 | 4.00 | 36.25 | 5.00 | 2.00 | 3.50 | 5.00 |
| 47 | TZDEEI50 | 70.00 | 72.50 | 19.00 | 1.00 | 1.42 | 0.75 | 2.00 | 10.50 | 1.50 | 69.25 | 4.00 | 2.00 | 5.50 | 4.50 |
| 48 | TZDEEI64 | 69.50 | 72.50 | 19.50 | 1.00 | 1.74 | 1.25 | 2.00 | 11.00 | 3.50 | 57.50 | 4.50 | 3.50 | 3.50 | 5.00 |
| 49 | **CML312** | **80.50** | **85.00** | 21.00 | **1.00** | **2.05** | **0.91** | **2.00** | **8.75** | **2.00** | **86.25** | **4.50** | 5.00 | **4.50** | **3.00** |
| 50 | CML444 | 78.50 | 81.00 | 19.00 | 1.00 | 1.50 | 0.67 | 2.00 | 12.50 | 3.00 | 31.75 | 4.50 | 9.50 | 5.00 | 4.00 |
| 51 | CML442 | 85.50 | 86.00 | 17.00 | 1.00 | 1.69 | 0.80 | 2.00 | 11.50 | 1.50 | 55.00 | 4.50 | 3.00 | 4.50 | 3.50 |
| 52 | TZDEEI54 | 90.50 | 91.00 | 17.00 | 1.00 | 1.00 | 0.75 | 2.00 | 10.50 | 3.50 | 47.50 | 5.50 | 6.50 | 5.00 | 2.00 |
| 53 | TZEEI10 | 86.50 | 84.50 | 14.50 | 1.00 | 1.65 | 0.92 | 2.00 | 12.75 | 6.50 | 26.00 | 4.50 | 3.00 | 3.50 | 3.50 |
| 54 | CML547 | 83.50 | 90.50 | 23.50 | 1.00 | 1.45 | 0.60 | 1.50 | 9.50 | 3.50 | 62.50 | 4.00 | 3.50 | 6.00 | 2.50 |
| 55 | **CML539** | **80.50** | **74.50** | 10.50 | **1.00** | **1.85** | **0.60** | **1.00** | **10.50** | **3.00** | **74.75** | **5.00** | 5.00 | **4.50** | **3.00** |
| 56 | **CML440** | **82.50** | **76.00** | 10.00 | **1.00** | **2.36** | **1.08** | **1.00** | **11.00** | **1.50** | **96.25** | **4.50** | 13.50 | **3.00** | **1.50** |
| 57 | **CML566** | **82.50** | **79.00** | 13.00 | **1.00** | **2.22** | **1.15** | **1.00** | **12.00** | **1.50** | **155.50** | **4.00** | 5.50 | **1.50** | **1.50** |
| 58 | **CML540** | **77.00** | **81.50** | 21.00 | **1.00** | **2.03** | **0.77** | **1.00** | **11.00** | **3.50** | **277.50** | **4.00** | 1.50 | **3.50** | **3.00** |
| 59 | CML545 | 75.50 | 74.00 | 15.00 | 1.00 | 1.73 | 0.67 | 1.00 | 10.25 | 3.00 | 53.00 | 4.50 | 6.00 | 5.00 | 3.00 |
| 60 | CML571 | 76.00 | 75.50 | 16.00 | 1.00 | 2.05 | 0.68 | 1.00 | 9.80 | 2.00 | 45.50 | 4.00 | 26.50 | 5.00 | 4.00 |
| 61 | CML390 | 80.00 | 81.00 | 17.50 | 1.00 | 2.10 | 0.74 | 1.00 | 11.30 | 2.50 | 56.00 | 4.50 | 7.00 | 5.00 | 3.50 |
| 62 | CLHP0352 | 76.50 | 76.50 | 16.50 | 1.00 | 2.30 | 0.85 | 1.00 | 11.75 | 3.00 | 51.25 | 4.50 | 11.50 | 5.00 | 3.00 |
| 63 | HA04A-2107-36 | 97.50 | 86.00 | 5.00 | 1.00 | 2.14 | 1.35 | 1.00 | 8.50 | 5.50 | 27.50 | 4.50 | 8.00 | 4.50 | 4.50 |
| 64 | **CLHP0303** | **84.50** | **83.50** | 15.50 | **1.50** | **1.87** | **1.15** | **1.00** | **7.25** | **3.00** | **92.50** | **4.50** | 8.50 | **3.00** | **3.00** |
| 65 | **CLHP0221** | **79.50** | **77.50** | 14.50 | **1.50** | **1.65** | **1.10** | **1.00** | **10.25** | **5.50** | **72.50** | **5.00** | 9.50 | **3.50** | **3.00** |
| 66 | **CLHP0020** | **71.00** | **77.00** | 22.50 | **1.00** | **1.80** | **0.57** | **1.00** | **12.00** | **2.50** | **119.00** | **4.50** | 2.00 | **3.50** | **3.50** |
| 67 | CLHP0058 | 78.50 | 76.50 | 14.50 | 1.00 | 1.40 | 0.46 | 1.00 | 8.50 | 4.50 | 26.00 | 5.00 | 3.00 | 5.50 | 2.50 |
| **68** | **CKDHL0378** | **72.00** | **75.00** | 19.50 | **1.00** | **1.90** | **0.75** | **1.00** | **11.75** | **2.00** | **85.00** | **4.50** | 17.50 | **3.00** | **2.50** |
| 69 | CLHP0312 | 80.50 | 79.50 | 15.50 | 1.00 | 1.75 | 0.76 | 1.00 | 9.50 | 2.00 | 45.75 | 4.50 | 18.50 | 4.50 | 3.50 |
| 70 | CLHP0310 | 76.00 | 76.50 | 17.00 | 1.00 | 1.91 | 1.03 | 1.00 | 11.75 | 4.00 | 35.75 | 5.00 | 13.50 | 5.00 | 3.50 |
| 71 | CLHP0003 | 82.00 | 83.00 | 17.50 | 1.00 | 1.40 | 0.82 | 1.00 | 9.00 | 5.50 | 30.00 | 6.00 | 4.50 | 2.50 | 3.00 |
| 72 | CKDHL0467 | 79.50 | 75.50 | 12.50 | 1.00 | 1.65 | 0.86 | 1.00 | 11.25 | 4.00 | 42.75 | 4.50 | 14.50 | 2.50 | 3.50 |
| 73 | CLHP00378 | 77.00 | 79.00 | 18.50 | 1.00 | 1.03 | 0.60 | 2.50 | 9.50 | 4.00 | 57.50 | 6.00 | 3.00 | 3.00 | 3.00 |
| 74 | **CLHP0156** | **80.50** | **78.50** | 14.50 | **1.50** | **2.40** | **0.98** | **1.00** | **12.00** | **1.50** | **74.50** | **5.00** | 8.00 | **1.50** | **2.00** |
| 75 | CLHP0113 | 77.50 | 74.50 | 13.50 | 1.00 | 1.65 | 0.50 | 2.50 | 10.00 | 5.00 | 30.50 | 5.00 | 4.00 | 3.50 | 3.50 |
| 76 | **CLHP03302** | **75.50** | **70.50** | 11.50 | **1.50** | **1.80** | **0.95** | **1.00** | **12.00** | **2.00** | **75.25** | **5.50** | 4.00 | **2.00** | **1.00** |
| 77 | CLHP0404 | 77.00 | 75.50 | 15.00 | 1.00 | 1.68 | 0.70 | 1.00 | 11.00 | 3.50 | 62.50 | 5.50 | 8.00 | 3.00 | 3.00 |
| 78 | **CLHP0343** | **77.50** | **77.00** | 16.00 | **1.00** | **1.60** | **0.77** | **1.00** | **11.50** | **1.00** | **82.00** | **4.50** | 2.00 | **4.00** | **4.50** |
| 79 | CZL1380 | 78.50 | 78.50 | 16.50 | 1.00 | 2.38 | 0.87 | 1.00 | 10.50 | 5.50 | 62.50 | 5.00 | 13.50 | 5.00 | 5.00 |
| 80 | **CLHP0326** | **75.00** | **77.00** | 18.50 | **1.00** | **1.40** | **0.66** | **1.00** | **10.50** | **2.00** | **82.25** | **4.50** | 2.00 | **3.00** | **2.50** |
| 81 | CZL99017 | 79.50 | 79.00 | 16.00 | 1.00 | 2.03 | 0.77 | 1.00 | 8.50 | 2.50 | 62.50 | 5.00 | 8.50 | 3.00 | 2.50 |
| 82 | TZEEI34 | 74.50 | 74.50 | 16.50 | 1.00 | 1.30 | 0.53 | 1.00 | 10.50 | 4.50 | 36.50 | 4.50 | 3.50 | 4.00 | 1.50 |
| 83 | **CLHP0049** | **80.50** | **78.00** | 14.00 | **1.00** | **1.25** | **0.70** | **1.00** | **10.00** | **3.00** | **101.25** | **7.00** | 4.00 | **4.00** | **2.50** |
| 84 | CLHP00478 | 79.50 | 74.50 | 11.50 | 1.00 | 1.73 | 0.70 | 1.00 | 11.50 | 3.00 | 56.00 | 5.00 | 20.50 | 3.50 | 3.50 |
| **85** | **CLHP00286** | **83.00** | **83.00** | 16.50 | **1.00** | **1.88** | **0.88** | **1.00** | **11.50** | **3.50** | **82.50** | **5.50** | 3.50 | **3.00** | **3.50** |
| 86 | **CML451** | **79.00** | **79.00** | 16.50 | **1.00** | **2.30** | **0.96** | **1.00** | **12.00** | **1.50** | **83.00** | **4.50** | 10.50 | **2.50** | **3.50** |
| 87 | **CLHP0302** | **81.00** | **80.50** | 16.00 | **1.00** | **1.76** | **1.00** | **3.00** | **13.25** | **3.00** | **98.00** | **4.50** | 7.00 | **5.00** | **3.50** |
| 88 | **CLHP0364** | **80.00** | **80.50** | 17.00 | **1.00** | **1.30** | **0.85** | **1.00** | **12.00** | **3.00** | **79.00** | **4.50** | 4.00 | **2.00** | **5.00** |
| 89 | **CLHP0350** | **75.00** | **76.00** | 17.50 | **1.00** | **2.35** | **0.81** | **3.00** | **14.00** | **3.50** | **102.75** | **5.00** | 3.50 | **2.00** | **3.50** |
| 90 | CLHP00294 | 88.00 | 87.50 | 16.00 | 1.00 | 2.00 | 0.75 | 1.00 | 9.50 | 5.50 | 40.00 | 5.00 | 1.50 | 5.00 | 5.00 |
| 91 | CLHP0005 | 76.00 | 77.50 | 18.00 | 1.00 | 2.03 | 0.76 | 1.00 | 10.50 | 3.50 | 58.75 | 4.50 | 19.00 | 2.00 | 4.50 |
| 92 | CLHP0022 | 81.00 | 81.50 | 17.00 | 1.00 | 1.50 | 0.55 | 1.00 | 7.00 | 4.00 | 12.50 | 5.50 | 3.00 | 3.00 | 5.50 |
| 93 | **CML304** | 82.50 | 82.50 | 16.50 | 1.00 | 1.75 | 0.95 | 2.00 | 11.00 | 4.00 | 59.25 | 6.50 | 33.50 | 4.50 | 6.50 |
| 94 | **TZISTR1174** | **84.00** | **82.50** | 15.00 | **1.00** | **1.80** | **1.00** | **1.50** | **12.25** | **2.00** | **93.25** | **3.00** | 47.00 | **3.00** | **3.50** |
| 95 | **TZISTR1205** | **81.50** | **75.50** | 10.50 | **1.00** | **1.85** | **0.91** | **1.00** | **11.00** | **1.00** | **114.25** | **3.50** | 13.00 | **3.00** | **4.50** |
| 96 | **TZSTRI113** | **78.00** | **77.00** | 15.50 | **1.00** | **1.75** | **0.95** | **1.00** | **10.50** | **1.50** | **87.00** | **3.00** | 7.50 | **1.50** | **6.00** |
| 97 | **TZISTR1119** | **84.00** | **80.50** | 13.00 | **1.00** | **1.91** | **1.15** | **1.00** | **11.25** | **4.00** | **84.75** | **3.00** | 19.00 | **2.50** | **4.50** |
| 98 | **TZISTR1015** | **81.00** | **77.00** | 12.50 | **1.00** | **1.77** | **0.85** | **1.00** | **10.50** | **1.00** | **73.25** | **2.50** | 5.50 | **3.50** | **4.00** |
| 99 | **ZM1421** | **80.50** | **81.00** | 17.00 | **1.00** | **1.00** | **0.70** | **1.00** | **9.00** | **3.50** | **77.50** | **5.00** | 4.50 | **1.00** | **2.00** |
| 100 | **B.King/1421** | **80.50** | **78.50** | 14.50 | **1.00** | **2.05** | **1.15** | **2.00** | **23.50** | **1.50** | **157.25** | **5.00** | 4.50 | **1.00** | **2.00** |
| 101 | Hickory/1421 | 70.50 | 69.50 | 15.50 | 1.00 | 1.95 | 1.30 | 2.00 | 15.50 | 5.00 | 0.00 | 4.50 | 27.00 | 3.00 | 3.00 |
| 102 | Kep/1421 | 69.50 | 73.00 | 20.00 | 1.00 | 2.30 | 1.75 | 1.00 | 17.50 | 5.00 | 25.75 | 4.50 | 2.00 | 2.50 | 1.50 |
| 103 | **Shesha/1421** | **75.50** | **72.50** | 13.50 | **1.00** | **2.03** | **1.75** | **1.00** | **18.75** | **1.50** | **165.75** | **7.50** | 18.50 | **2.00** | **2.00** |
| 104 | **ZM1423** | **69.00** | **69.50** | 17.00 | **1.00** | **0.85** | **1.39** | **1.00** | **10.50** | **1.50** | **99.25** | **4.50** | 16.50 | **5.00** | **3.00** |
| 105 | **N.Choice/1421** | **82.00** | **76.50** | 11.00 | **1.00** | **1.90** | **1.03** | **1.00** | **13.25** | **1.50** | **214.00** | **5.00** | 4.00 | **3.50** | **3.50** |
| 106 | NC.QPM/Z.DPLO | **79.50** | **74.50** | 11.50 | **1.00** | **2.05** | **0.88** | **1.00** | **15.50** | **1.50** | **169.50** | **4.50** | 3.50 | **2.50** | **3.00** |
| 107 | STR-SYN-Y2 | 81.50 | 82.00 | 17.00 | 1.00 | 2.40 | 0.98 | 1.00 | 11.50 | 6.00 | 44.50 | 6.00 | 2.50 | 3.50 | 5.50 |
| 108 | Z. Diplo.BC4C3-W-DT C1 | 88.00 | 88.50 | 17.00 | 1.00 | 2.45 | 0.95 | 1.50 | 9.00 | 5.50 | 42.50 | 6.00 | 2.00 | 4.00 | 5.00 |
| 109 | **TZBSTR (Susceptible)** | **83.00** | **83.50** | 17.00 | **1.00** | **2.65** | **1.30** | **1.50** | **14.50** | **1.00** | **103.00** | **6.50** | 3.00 | **3.00** | **2.50** |
| 110 | STR-SYN-W1 | 80.00 | 79.50 | 16.00 | 1.00 | 2.55 | 1.18 | 1.50 | 13.50 | 4.00 | 53.50 | 6.50 | 14.50 | 3.50 | 4.00 |
| 111 | **DTSTR-W SYN13** | **85.50** | **85.50** | 16.50 | **1.00** | **1.25** | **0.85** | **1.50** | **13.00** | **3.50** | **107.50** | **4.50** | 3.50 | **1.50** | **5.00** |
| 112 | DTSTR-Y SYN15 | 77.00 | 72.50 | 12.00 | 1.00 | 2.80 | 1.30 | 1.00 | 12.75 | 4.00 | 57.50 | 4.50 | 2.50 | 2.00 | 3.00 |
| 113 | ((IWD C3 SYN*2/(White DT STR Syn))-DT C1 | 89.00 | 91.50 | 19.00 | 1.00 | 2.45 | 0.75 | 1.50 | 11.50 | 4.00 | 35.00 | 5.50 | 3.50 | 3.50 | 6.00 |
| 114 | **DTSTR-W SYN11** | **78.50** | **77.50** | 15.50 | **1.00** | **2.30** | **1.23** | **1.50** | **13.00** | **2.50** | **71.25** | **5.00** | 52.50 | **3.50** | **5.50** |
| 115 | SAMMMZ16 | 86.50 | 87.00 | 17.00 | 1.00 | 2.50 | 1.10 | 1.00 | 11.75 | 3.00 | 54.25 | 5.00 | 11.00 | 4.50 | 4.50 |
| 116 | **(TZEOMP5C7/TZECOMP3DTC2) C2** | **75.00** | **75.50** | 17.00 | **1.00** | **2.38** | **1.10** | **1.50** | **14.00** | **1.00** | **89.50** | **4.00** | 32.00 | **4.00** | **5.00** |
| 117 | ((TZL COMP1-W C6*2/(White DT STR Syn))-DT C1 | 85.00 | 86.50 | 18.00 | 1.00 | 1.40 | 0.85 | 1.00 | 11.00 | 3.50 | 62.50 | 3.00 | 2.50 | 2.50 | 3.00 |
| 118 | TZCOM1/ZDPSYN | 74.00 | 69.50 | 12.00 | 1.00 | 2.15 | 1.02 | 1.00 | 15.00 | 3.50 | 61.25 | 3.00 | 10.00 | 3.00 | 4.00 |
| 119 | **Colorado/1421** | **87.50** | **89.50** | 18.50 | **1.00** | **2.03** | **0.70** | **1.00** | **13.75** | **2.00** | **75.25** | **2.50** | 3.00 | **3.50** | **5.00** |
| 120 | M.Pearl/DT-STR | 73.50 | 74.00 | 17.00 | 1.00 | 2.38 | 1.04 | 0.50 | 13.50 | 3.50 | 61.50 | 2.00 | 17.00 | 2.50 | 3.00 |
| 121 | **Z.diplo-BC4-C3-W/DOGONA-1/Z.diplo-BC4-C3-W** | **81.00** | **83.00** | 18.50 | **1.00** | **2.36** | **1.05** | **1.00** | **11.50** | **1.50** | **112.00** | **6.00** | 8.50 | **3.00** | **3.50** |
| 122 | NC.QPM/DT-STR | 71.50 | 71.50 | 16.50 | 1.00 | 2.25 | 1.03 | 0.00 | 12.50 | 4.50 | 54.25 | 3.00 | 5.00 | 3.50 | 5.00 |
| 123 | **ZM1421/DT-STR** | **77.00** | **76.50** | 16.00 | **1.00** | **2.38** | **1.10** | **1.50** | **10.75** | **3.00** | **93.50** | **2.50** | 19.00 | **2.50** | **6.00** |
| 124 | **DTSTR-Y SYN14** | **78.50** | **74.50** | 12.50 | **1.00** | **2.23** | **1.10** | **0.50** | **13.75** | **5.00** | **78.50** | **2.50** | 8.50 | **3.50** | **3.50** |
| 125 | **(2*TZECOMP3DT/WhiteDTSTRSYN) C2** | **69.00** | **78.00** | 25.50 | **1.00** | **1.75** | **0.85** | **1.50** | **12.25** | **2.50** | **89.00** | **6.00** | 0.50 | **2.00** | **5.50** |
| 126 | ZM1423/Z.DLO | 84.50 | 85.00 | 17.00 | 1.00 | 2.50 | 1.05 | 1.50 | 13.25 | 2.00 | 64.25 | 3.50 | 6.00 | 2.50 | 4.00 |
