# Supplementary material for: Genetic diversity analysis of tropical and sub-tropical maize germplasm for Striga resistance and agronomic traits with SNP markers
Source: PLoS One. 2024 Aug 6;19(8):e0306263. doi: 10.1371/journal.pone.0306263 (PMC11302897; doi:10.1371/journal.pone.0306263)
Supplement: S5 Table — (DOCX) [file pone.0306263.s005.docx]

**Supporting information:** Mean responses for 14 maize and *Striga* parameters assessed from 126 maize genotypes evaluated under *Striga hermonthica* infestation with anthesis silking interval normalized.

| **N°** | **Accessions** | **DA** | **DS** | **ASI** | **EPP** | **PLHT** | **EHT** | **HUSK** | **CL** | **EASP** | **GY** | **SEC8** | **SEC10** | **SDR8** | **SDR10** |
| --- | --- | --- | --- | --- | --- | --- | --- | --- | --- | --- | --- | --- | --- | --- | --- |
|  |  |  |  |  |  | **(m)** | **(m)** | **(1 to 5)** | **(cm)** | **(1 to 9)** | **(g/plant)** |  |  | **(1 to 9)** | **(1 to 9)** |
| 1 | TZISTR1154 | 78.50 | 79.50 | 8.00 | 1.50 | 2.25 | 1.25 | 1.00 | 11.00 | 3.00 | 67.25 | 2.50 | 1.50 | 3.00 | 2.25 |
| 2 | **TZISTR1261** | **78.63** | **80.63** | 9.00 | **2.00** | **2.20** | **0.97** | **2.00** | **10.96** | **3.25** | **70.25** | **3.75** | 4.50 | **2.00** | **1.75** |
| 3 | **TZISTR1248** | **75.88** | **77.25** | 8.37 | **1.50** | **1.75** | **0.77** | **2.00** | **11.96** | **2.25** | **81.25** | **4.75** | 1.50 | **2.25** | **2.00** |
| 4 | **TZISTR1263** | **76.25** | **78.25** | 9.00 | **1.00** | **1.35** | **0.59** | **1.00** | **10.96** | **3.75** | **76.00** | **3.25** | 9.00 | **3.75** | **2.75** |
| 5 | TZISTR1275 | 82.00 | 83.00 | 8.00 | 1.00 | 1.50 | 0.49 | 1.00 | 9.96 | 5.25 | 65.25 | 3.25 | 6.00 | 4.50 | 3.50 |
| 6 | TZISTR1157 | 81.13 | 81.25 | 7.12 | 1.00 | 1.55 | 0.54 | 2.00 | 9.96 | 5.25 | 65.25 | 3.75 | 3.50 | 2.25 | 3.50 |
| 7 | TZISTR1160 | 79.88 | 80.50 | 7.62 | 1.00 | 1.47 | 0.54 | 2.00 | 10.46 | 3.75 | 57.25 | 2.75 | 6.50 | 3.75 | 2.75 |
| 8 | TZISTR1162 | 81.70 | 84.13 | 9.43 | 1.00 | 1.70 | 0.71 | 1.00 | 12.21 | 2.25 | 63.75 | 2.25 | 3.00 | 3.00 | 3.00 |
| 9 | TZISTR1165 | 82.38 | 81.75 | 6.37 | 1.00 | 2.10 | 0.99 | 1.00 | 13.46 | 2.75 | 66.25 | 3.25 | 3.50 | 2.75 | 3.00 |
| 10 | TZISTR1175 | 79.00 | 78.25 | 6.25 | 1.00 | 2.10 | 0.92 | 1.00 | 11.71 | 5.75 | 59.75 | 3.75 | 5.50 | 2.75 | 1.75 |
| 11 | TZISTR1178 | 74.50 | 74.00 | 6.50 | 1.00 | 2.35 | 0.94 | 1.00 | 9.96 | 6.75 | 65.25 | 3.25 | 3.50 | 3.75 | 1.75 |
| 12 | TZISTR1163 | 76.75 | 77.50 | 7.75 | 1.00 | 2.25 | 1.07 | 1.00 | 9.46 | 3.25 | 65.00 | 3.25 | 1.00 | 3.50 | 3.00 |
| 13 | TZISTR1166 | 90.00 | 87.50 | 4.50 | 1.00 | 1.60 | 0.77 | 1.00 | 9.46 | 3.25 | 65.00 | 3.25 | 6.50 | 2.75 | 2.00 |
| 14 | TZISTR1190 | 93.50 | 92.00 | 5.50 | 1.00 | 1.45 | 0.69 | 1.50 | 8.46 | 3.25 | 67.00 | 3.75 | 5.00 | 4.25 | 2.25 |
| 15 | TZISTR1199 | 82.75 | 81.00 | 5.25 | 1.00 | 1.85 | 0.97 | 1.50 | 8.46 | 3.25 | 64.25 | 3.25 | 2.00 | 3.25 | 2.75 |
| 16 | TZISTR1231 | 73.25 | 72.25 | 6.00 | 1.00 | 1.85 | 0.94 | 1.00 | 9.21 | 7.25 | 62.50 | 2.75 | 6.50 | 3.75 | 2.25 |
| 17 | TZISTR1232 | 77.50 | 76.75 | 6.25 | 1.00 | 1.83 | 0.94 | 1.00 | 10.71 | 8.75 | 46.75 | 3.25 | 2.00 | 2.00 | 2.00 |
| 18 | TZISTR1259 | 77.75 | 78.75 | 8.00 | 1.00 | 1.78 | 0.89 | 2.00 | 13.46 | 8.25 | 25.75 | 3.25 | 3.50 | 3.00 | 2.25 |
| 19 | TZISTR1262 | 76.75 | 74.50 | 4.75 | 1.00 | 1.65 | 0.73 | 2.50 | 10.71 | 5.75 | 39.25 | 2.63 | 5.00 | 3.75 | 3.00 |
| 20 | TZISTR1159 | 76.50 | 72.75 | 3.25 | 1.00 | 1.85 | 0.86 | 1.50 | 8.46 | 5.25 | 46.25 | 3.13 | 2.00 | 1.75 | 2.75 |
| 21 | TZISTR1223 | 80.88 | 78.00 | 4.12 | 1.00 | 2.10 | 0.97 | 1.00 | 9.46 | 5.25 | 55.00 | 3.13 | 2.50 | 3.25 | 2.75 |
| 22 | TZISTR1225 | 84.75 | 83.25 | 5.50 | 1.00 | 2.00 | 0.88 | 1.00 | 8.71 | 3.25 | 65.25 | 2.63 | 4.50 | 3.25 | 3.75 |
| 23 | CML550 | **73.25** | **73.25** | 7.00 | **1.00** | **2.13** | **1.00** | **1.00** | **13.21** | **1.25** | **86.00** | **2.63** | 5.00 | **1.50** | **2.00** |
| 24 | TZISTR1244 | 79.25 | 75.25 | 3.00 | 1.00 | 1.55 | 0.82 | 1.50 | 8.46 | 1.75 | 48.25 | 2.63 | 7.00 | 2.25 | 2.00 |
| 25 | TZSTRI101 | **90.00** | **87.50** | 4.50 | **1.00** | **1.45** | **0.75** | **1.00** | **12.46** | **3.25** | **144.00** | **3.63** | 6.50 | **3.75** | **3.00** |
| 26 | TZSTRI102 | 87.75 | 84.75 | 4.00 | 1.00 | 1.45 | 0.75 | 1.00 | 12.96 | 5.25 | 51.75 | 3.13 | 4.50 | 3.00 | 2.00 |
| 27 | TZSTRI104 | 89.50 | 90.75 | 8.25 | 1.00 | 1.45 | 0.75 | 1.00 | 12.96 | 5.25 | 51.75 | 3.13 | 7.50 | 3.50 | 1.75 |
| **28** | **TZSTRI107** | **88.00** | **86.75** | 5.75 | **1.00** | **1.25** | **0.66** | **1.00** | **8.75** | **3.25** | **70.25** | **3.63** | 8.00 | **2.25** | **2.50** |
| 29 | TZSTRI108 | **77.25** | **75.00** | 4.75 | **1.00** | **1.55** | **0.88** | **1.00** | **8.75** | **1.25** | **77.00** | **3.13** | 1.50 | **3.50** | **2.50** |
| 30 | TZSTRI109 | **80.75** | **78.00** | 4.25 | **1.00** | **1.75** | **0.97** | **1.00** | **11.00** | **2.25** | **78.00** | **3.13** | 4.50 | **3.75** | **2.50** |
| 31 | TZSTRI110 | **80.75** | **79.75** | 6.00 | **1.00** | **1.95** | **1.10** | **1.00** | **10.25** | **3.25** | **71.00** | **3.13** | 4.00 | **2.25** | **2.75** |
| 32 | TZSTRI112 | 80.38 | 82.50 | 9.12 | 1.00 | 1.55 | 0.82 | 1.00 | 9.25 | 4.25 | 62.00 | 3.13 | 5.50 | 3.75 | 2.75 |
| 33 | TZSTRI114 | 87.75 | 91.00 | 10.25 | 1.00 | 1.10 | 0.47 | 1.00 | 10.00 | 5.25 | 62.25 | 3.13 | 2.50 | 3.00 | 2.00 |
| 34 | **TZSTRI115** | **84.75** | **87.75** | 10.00 | **1.00** | **1.65** | **0.77** | **1.00** | **10.50** | **3.25** | **70.00** | **3.20** | 1.50 | **1.00** | **1.25** |
| 35 | **TZISTR25** | **75.75** | **75.50** | 6.75 | **1.00** | **2.25** | **1.05** | **1.00** | **12.00** | **1.25** | **131.00** | **3.20** | 2.00 | **3.75** | **2.00** |
| 36 | **TZISTR1001** | **79.63** | **78.25** | 5.62 | **1.00** | **2.10** | **1.03** | **1.00** | **11.50** | **1.75** | **120.00** | **2.70** | 1.50 | **3.50** | **2.50** |
| 37 | TZISTR1003 | 81.38 | 79.50 | 5.12 | 1.00 | 1.53 | 0.91 | 1.00 | 10.00 | 3.75 | 59.00 | 3.20 | 1.50 | 3.50 | 2.25 |
| 38 | TZISTR1004 | 80.25 | 78.75 | 5.50 | 1.00 | 1.45 | 0.84 | 1.00 | 9.00 | 3.25 | 62.25 | 4.20 | 3.50 | 3.75 | 2.50 |
| 39 | TZISTR1008 | 81.25 | 79.25 | 5.00 | 1.00 | 1.70 | 0.89 | 1.00 | 9.00 | 3.25 | 62.25 | 4.70 | 1.50 | 2.75 | 2.75 |
| 40 | TZISTR1011 | 86.50 | 84.75 | 5.25 | 1.00 | 2.05 | 0.96 | 1.00 | 10.00 | 7.25 | 39.75 | 4.20 | 1.50 | 3.00 | 2.00 |
| 41 | TZISTR1018 | 81.00 | 81.50 | 7.50 | 1.00 | 2.05 | 1.03 | 1.00 | 9.50 | 8.25 | 22.75 | 3.20 | 6.00 | 3.75 | 4.00 |
| 42 | TZEEI21 | 77.00 | 77.88 | 7.88 | 1.00 | 1.37 | 0.75 | 1.00 | 9.50 | 6.25 | 45.25 | 3.20 | 5.00 | 3.75 | 4.75 |
| 43 | TZEEI13 | 80.75 | 81.25 | 7.50 | 1.00 | 1.27 | 0.65 | 1.00 | 8.50 | 3.25 | 55.00 | 3.70 | 2.00 | 3.25 | 2.25 |
| 44 | TZEEI14 | 77.25 | 79.00 | 8.75 | 1.00 | 1.97 | 1.10 | 1.00 | 10.75 | 2.75 | 51.75 | 3.20 | 3.50 | 2.00 | 2.00 |
| 45 | TZEEI49 | 76.25 | 74.75 | 5.50 | 1.00 | 2.05 | 1.06 | 1.00 | 11.75 | 4.25 | 48.25 | 3.20 | 2.00 | 3.75 | 2.50 |
| 46 | TZDEEI55 | 78.38 | 76.75 | 5.37 | 1.00 | 1.38 | 0.61 | 1.00 | 9.50 | 4.75 | 51.50 | 2.70 | 2.00 | 3.75 | 2.75 |
| **47** | **TZDEEI50** | **74.50** | **74.50** | 7.00 | **1.00** | **1.29** | **0.57** | **1.00** | **10.50** | **3.75** | **70.00** | **1.70** | 5.50 | **5.50** | **5.00** |
| 48 | TZDEEI64 | 70.75 | 74.25 | 10.50 | 1.00 | 1.72 | 0.85 | 1.00 | 10.50 | 4.25 | 52.00 | 2.20 | 2.00 | 2.00 | 4.25 |
| 49 | CML312 | 74.50 | 72.63 | 5.13 | 1.00 | 2.02 | 0.88 | 1.00 | 8.50 | 6.25 | 27.25 | 3.20 | 2.00 | 3.75 | 1.00 |
| 50 | CML444 | 77.75 | 77.50 | 6.75 | 1.00 | 1.57 | 0.68 | 1.00 | 7.00 | 4.25 | 42.50 | 2.20 | 1.50 | 3.75 | 1.75 |
| 51 | CML442 | 79.25 | 80.25 | 8.00 | 1.00 | 1.32 | 0.63 | 1.00 | 6.75 | 4.75 | 45.00 | 1.70 | 2.00 | 3.25 | 2.25 |
| **52** | **TZDEEI54** | **83.75** | **79.25** | 2.50 | **1.00** | **1.52** | **0.80** | **1.50** | **13.00** | **3.25** | **82.50** | **3.20** | 1.50 | **2.75** | **0.75** |
| 53 | TZEEI10 | 82.25 | 81.25 | 6.00 | 1.00 | 1.32 | 0.71 | 1.00 | 11.25 | 3.25 | 40.00 | 2.70 | 2.00 | 3.50 | 1.50 |
| 54 | CML547 | 86.75 | 86.25 | 6.50 | 1.00 | 1.32 | 0.71 | 1.50 | 11.25 | 3.25 | 40.00 | 2.70 | 2.00 | 3.50 | 2.25 |
| **55** | **CML539** | **75.50** | **73.00** | 4.50 | **1.00** | **1.59** | **0.77** | **1.50** | **8.00** | **3.25** | **70.25** | **3.18** | 5.50 | **0.75** | **1.50** |
| **56** | **CML440** | **80.50** | **79.25** | 5.75 | **1.00** | **1.62** | **1.17** | **1.00** | **9.50** | **1.25** | **84.50** | **2.70** | 6.50 | **2.75** | **1.75** |
| **57** | **CML566** | **78.00** | **76.00** | 5.00 | **1.00** | **2.07** | **1.20** | **1.00** | **12.00** | **1.25** | **127.00** | **2.25** | 4.50 | **1.75** | **2.75** |
| **58** | **CML540** | **77.25** | **76.75** | 6.50 | **1.00** | **2.17** | **0.89** | **1.00** | **11.00** | **3.25** | **108.00** | **2.60** | 4.00 | **1.50** | **2.00** |
| **59** | **CML545** | **77.50** | **76.00** | 5.50 | **1.00** | **2.15** | **0.87** | **1.00** | **10.00** | **3.25** | **83.00** | **2.68** | 5.00 | **3.75** | **3.50** |
| **60** | **CML571** | **76.25** | **75.75** | 6.50 | **1.00** | **12.45** | **0.78** | **3.00** | **10.00** | **3.25** | **87.75** | **2.63** | 2.00 | **3.25** | **3.75** |
| 61 | CML390 | 74.00 | 73.75 | 6.75 | 1.00 | 2.05 | 0.80 | 3.00 | 10.75 | 5.75 | 37.30 | 2.68 | 7.50 | 4.25 | 3.25 |
| 62 | CLHP0352 | 78.50 | 74.75 | 3.25 | 1.00 | 1.10 | 0.98 | 1.00 | 11.25 | 6.75 | 17.05 | 2.68 | 8.00 | 3.75 | 3.75 |
| 63 | HA04A-2107-36 | 86.25 | 88.25 | 9.00 | 1.00 | 2.00 | 0.96 | 1.50 | 8.50 | 8.25 | 10.05 | 2.18 | 0.00 | 3.00 | 2.00 |
| 64 | CLHP0303 | 77.25 | 73.75 | 3.50 | 1.50 | 1.52 | 1.06 | 1.50 | 8.00 | 6.25 | 54.55 | 2.68 | 4.00 | 3.75 | 2.00 |
| 65 | CLHP0221 | 83.75 | 81.25 | 4.50 | 1.50 | 1.75 | 0.89 | 1.50 | 10.00 | 6.25 | 54.55 | 3.18 | 5.00 | 1.50 | 1.50 |
| 66 | CLHP0020 | 74.25 | 71.75 | 4.50 | 1.00 | 1.64 | 0.71 | 1.50 | 10.50 | 8.75 | 22.30 | 3.18 | 2.50 | 1.75 | 1.50 |
| 67 | CLHP0058 | 76.75 | 76.25 | 6.50 | 1.00 | 1.13 | 0.58 | 1.50 | 10.50 | 7.75 | 22.30 | 5.18 | 2.00 | 5.75 | 4.75 |
| 68 | CKDHL0378 | 80.00 | 79.75 | 6.75 | 1.00 | 1.55 | 0.78 | 1.50 | 10.00 | 4.25 | 28.05 | 5.18 | 2.00 | 4.75 | 5.75 |
| 69 | CLHP0312 | 76.25 | 78.00 | 8.75 | 1.00 | 1.35 | 0.95 | 1.50 | 8.50 | 2.75 | 35.55 | 3.18 | 1.50 | 3.75 | 3.75 |
| 70 | CLHP0310 | 72.75 | 72.50 | 6.75 | 1.00 | 1.20 | 0.77 | 1.50 | 8.00 | 5.75 | 23.55 | 2.68 | 2.50 | 3.75 | 2.75 |
| **71** | **CLHP0003** | **72.75** | **74.25** | 8.50 | **1.00** | **1.85** | **0.88** | **1.00** | **12.75** | **5.25** | **90.80** | **2.68** | 7.00 | **2.00** | **2.50** |
| **72** | **CKDHL0467** | **82.00** | **82.75** | 7.75 | **1.00** | **1.40** | **1.04** | **1.00** | **13.50** | **5.25** | **96.80** | **2.68** | 3.00 | **4.25** | **3.50** |
| 73 | CLHP00378 | 75.25 | 74.25 | 6.00 | 1.00 | 1.10 | 0.72 | 1.00 | 8.25 | 7.25 | 25.05 | 2.68 | 4.00 | 4.50 | 4.50 |
| 74 | CLHP0156 | 73.25 | 72.50 | 6.25 | 1.50 | 2.28 | 0.86 | 1.00 | 11.75 | 7.75 | 26.30 | 2.68 | 3.00 | 2.50 | 4.25 |
| 75 | CLHP0113 | 77.25 | 76.00 | 5.75 | 1.50 | 1.25 | 0.92 | 1.00 | 12.00 | 8.25 | 39.05 | 2.68 | 2.00 | 2.75 | 2.50 |
| 76 | CLHP03302 | 74.00 | 71.75 | 4.75 | 1.00 | 0.90 | 0.47 | 1.00 | 8.25 | 8.75 | 46.05 | 2.68 | 2.50 | 4.25 | 2.25 |
| **77** | **CLHP0404** | **74.75** | **74.25** | 6.50 | **1.00** | **2.07** | **0.75** | **1.00** | **10.00** | **6.25** | **137.35** | **3.18** | 5.00 | **6.00** | **4.00** |
| **78** | **CLHP0343** | **79.13** | **81.00** | 8.87 | **1.00** | **1.37** | **0.92** | **1.00** | **11.50** | **2.25** | **101.10** | **3.68** | 2.50 | **5.00** | **5.25** |
| 79 | CZL1380 | 80.25 | 79.25 | 6.00 | 1.00 | 1.23 | 0.79 | 1.00 | 9.50 | 5.25 | 54.85 | 3.68 | 5.00 | 5.25 | 5.50 |
| 80 | CLHP0326 | **72.75** | **72.25** | 6.50 | **1.00** | **1.60** | **0.72** | **1.50** | **8.50** | **5.25** | **85.35** | **3.68** | 2.00 | **3.25** | **4.75** |
| **81** | **CZL99017** | **81.75** | **82.25** | 7.50 | **1.00** | **1.70** | **0.68** | **1.50** | **10.75** | **2.25** | **71.10** | **3.18** | 5.50 | **3.50** | **3.00** |
| 82 | TZEEI34 | 79.25 | 80.25 | 8.00 | 1.00 | 1.26 | 0.58 | 1.50 | 10.25 | 4.25 | 60.10 | 3.18 | 6.50 | 4.00 | 2.25 |
| 83 | CLHP0049 | 78.25 | 79.25 | 8.00 | 1.00 | 1.25 | 0.47 | 1.50 | 5.50 | 5.25 | 60.35 | 3.18 | 2.50 | 3.25 | 2.50 |
| 84 | CLHP00478 | 77.75 | 75.25 | 4.50 | 1.00 | 1.60 | 0.55 | 1.00 | 5.25 | 5.75 | 57.35 | 2.68 | 2.00 | 2.50 | 2.00 |
| 85 | CLHP00286 | 77.88 | 79.50 | 8.62 | 1.00 | 1.25 | 0.70 | 1.00 | 9.75 | 5.75 | 57.60 | 3.68 | 3.00 | 3.25 | 2.00 |
| 86 | CML451 | 85.75 | 86.25 | 7.50 | 1.00 | 1.65 | 0.84 | 1.00 | 11.25 | 4.25 | 62.85 | 3.68 | 4.50 | 4.50 | 2.50 |
| 87 | CLHP0302 | 82.75 | 79.75 | 4.00 | 1.00 | 1.40 | 0.79 | 2.00 | 13.75 | 4.25 | 32.10 | 3.18 | 3.50 | 4.25 | 3.00 |
| 88 | CLHP0364 | 80.25 | 80.25 | 7.00 | 1.00 | 1.45 | 0.57 | 2.00 | 12.50 | 5.25 | 39.35 | 3.68 | 4.50 | 3.75 | 2.75 |
| 89 | CLHP0350 | 77.25 | 78.25 | 8.00 | 1.00 | 1.85 | 0.62 | 1.00 | 10.50 | 6.25 | 40.85 | 3.18 | 2.00 | 3.25 | 3.25 |
| 90 | CLHP00294 | 88.50 | 85.75 | 4.25 | 1.00 | 1.07 | 0.47 | 1.50 | 9.25 | 7.25 | 25.10 | 3.18 | 2.50 | 3.50 | 3.00 |
| 91 | CLHP0005 | 84.25 | 85.75 | 8.50 | 1.00 | 1.10 | 0.32 | 1.50 | 8.75 | 6.25 | 67.10 | 6.68 | 5.50 | 5.75 | 2.50 |
| 92 | CLHP0022 | 71.75 | 73.88 | 9.13 | 1.00 | 1.45 | 0.45 | 1.00 | 9.00 | 5.25 | 44.60 | 9.68 | 2.00 | 4.00 | 2.00 |
| **93** | **CML304** | **79.25** | **79.63** | 7.38 | **1.00** | **1.44** | **0.75** | **1.00** | **12.00** | **4.75** | **151.00** | **3.18** | 2.00 | **4.75** | **2.75** |
| **94** | **TZISTR1174** | **83.75** | **82.00** | 5.25 | **1.00** | **1.68** | **0.91** | **1.00** | **11.00** | **1.75** | **112.75** | **4.18** | 4.50 | **5.00** | **3.50** |
| **95** | **TZISTR1205** | **81.00** | **83.00** | 9.00 | **1.00** | **2.21** | **1.00** | **1.00** | **9.50** | **1.75** | **129.00** | **4.18** | 1.00 | **3.75** | **2.50** |
| **96** | **TZSTRI113** | **74.50** | **73.00** | 5.50 | **1.00** | **1.41** | **0.90** | **1.00** | **9.00** | **1.75** | **111.75** | **2.68** | 3.50 | **3.75** | **2.75** |
| **97** | **TZISTR1119** | **78.75** | **77.00** | 5.25 | **1.00** | **1.81** | **0.95** | **1.00** | **10.50** | **3.75** | **135.75** | **3.68** | 4.50 | **5.50** | **3.50** |
| **98** | **TZISTR1015** | **80.00** | **80.50** | 7.50 | **1.00** | **1.51** | **0.92** | **1.00** | **9.50** | **3.75** | **74.50** | **3.68** | 4.00 | **1.25** | **3.50** |
| **99** | **ZM1421** | **82.38** | **80.75** | 5.37 | **1.00** | **2.10** | **0.95** | **1.50** | **10.71** | **2.25** | **88.00** | **2.63** | 2.00 | **1.75** | **3.25** |
| **100** | **B.King/1421** | **81.00** | **77.75** | 3.75 | **1.00** | **2.35** | **1.05** | **1.50** | **11.71** | **3.25** | **91.75** | **2.63** | 4.50 | **2.25** | **2.25** |
| 101 | Hickory/1421 | 79.25 | 77.75 | 5.50 | 1.00 | 2.40 | 1.18 | 1.50 | 7.96 | 7.25 | 39.75 | 2.63 | 5.00 | 2.00 | 2.50 |
| 102 | Kep/1421 | 72.50 | 73.50 | 8.00 | 1.00 | 2.10 | 1.12 | 2.00 | 8.21 | 5.75 | 34.75 | 2.63 | 4.00 | 2.50 | 2.75 |
| **103** | **Shesha/1421** | **71.50** | **70.75** | 6.25 | **1.00** | **1.82** | **0.88** | **1.50** | **10.71** | **1.75** | **112.25** | **4.63** | 2.50 | **4.00** | **2.75** |
| **104** | **ZM1423** | **70.25** | **71.88** | 8.63 | **1.00** | **2.17** | **0.94** | **1.00** | **13.71** | **1.25** | **144.25** | **4.63** | 2.50 | **1.75** | **2.50** |
| **105** | **N.Choice/1421** | **81.25** | **75.25** | 1.00 | **1.00** | **1.62** | **0.85** | **1.50** | **10.96** | **1.75** | **133.25** | **3.13** | 3.50 | **4.00** | **3.00** |
| 106 | NC.QPM/Z.DPLO | 82.50 | 81.25 | 5.75 | 1.00 | 1.77 | 0.86 | 1.50 | 11.25 | 4.25 | 68.00 | 3.20 | 5.00 | 2.75 | 1.25 |
| **107** | **STR-SYN-Y2** | **85.25** | **85.25** | 7.00 | **1.00** | **1.60** | **0.80** | **1.00** | **11.25** | **3.25** | **126.85** | **8.18** | 3.50 | **3.25** | **2.50** |
| 108 | Z. Diplo.BC4C3-W-DT C1 | 87.75 | 87.75 | 7.00 | 1.00 | 0.98 | 0.82 | 1.00 | 12.25 | 3.25 | 60.35 | 6.68 | 2.50 | 4.75 | 4.25 |
| 109 | TZBSTR (Susceptible)(RE) | 84.25 | 87.75 | 10.50 | 1.00 | 1.65 | 0.66 | 1.00 | 10.50 | 4.25 | 33.60 | 8.18 | 1.50 | 4.75 | 4.25 |
| 110 | STR-SYN-W1 | 79.00 | 79.00 | 7.00 | 1.00 | 2.12 | 0.94 | 1.50 | 10.50 | 2.75 | 51.10 | 8.18 | 5.00 | 3.25 | 3.25 |
| **111** | **DTSTR-W SYN13** | **89.25** | **88.50** | 6.25 | **1.00** | **0.98** | **0.75** | **1.50** | **10.00** | **3.75** | **115.35** | **4.68** | 5.50 | **3.50** | **2.50** |
| **112** | **DTSTR-Y SYN15** | **83.75** | **84.25** | 7.50 | **1.00** | **1.78** | **0.65** | **1.00** | **9.00** | **6.25** | **87.35** | **3.18** | 4.50 | **4.00** | **2.75** |
| 113 | ((IWD C3 SYN*2/(White DT STR Syn))-DT C1 | 84.25 | 84.50 | 7.25 | 1.00 | 1.48 | 0.65 | 1.00 | 8.50 | 6.25 | 57.10 | 3.18 | 3.50 | 3.75 | 3.00 |
| 114 | DTSTR-W SYN11 | 87.25 | 89.75 | 9.50 | 1.00 | 1.73 | 0.73 | 1.50 | 8.00 | 3.75 | 45.10 | 2.68 | 3.00 | 2.75 | 1.75 |
| 115 | SAMMMZ16 | 85.75 | 84.75 | 6.00 | 1.00 | 1.93 | 1.03 | 2.00 | 8.50 | 3.75 | 56.50 | 3.18 | 4.50 | 2.75 | 0.75 |
| 116 | (TZEOMP5C7/TZECOMP3DTC2) C2 | 84.50 | 82.75 | 5.25 | 1.00 | 1.33 | 0.96 | 2.00 | 9.25 | 5.75 | 59.50 | 3.68 | 3.00 | 3.75 | 2.25 |
| 117 | ((TZL COMP1-W C6*2/(White DT STR Syn))-DT C1 | 87.75 | 88.75 | 8.00 | 1.00 | 1.73 | 0.66 | 2.00 | 9.25 | 5.75 | 59.50 | 3.68 | 4.50 | 3.75 | 3.25 |
| 118 | TZCOM1/ZDPSYN | 79.25 | 75.75 | 3.50 | 1.00 | 1.66 | 0.31 | 2.00 | 11.50 | 3.25 | 62.50 | 3.18 | 5.50 | 4.75 | 3.75 |
| 119 | Colorado/1421 | 77.75 | 79.63 | 8.88 | 1.00 | 1.63 | 0.49 | 2.00 | 10.50 | 1.25 | 58.75 | 4.68 | 7.50 | 5.25 | 5.25 |
| 120 | M.Pearl/DT-STR | 87.25 | 87.75 | 7.50 | 1.00 | 0.73 | 0.65 | 1.00 | 9.00 | 3.25 | 57.25 | 5.68 | 2.00 | 6.50 | 5.00 |
| 121 | Z.diplo-BC4-C3-W/DOGONA-1/Z.diplo-BC4-C3-W | 79.13 | 78.75 | 6.62 | 1.00 | 1.34 | 0.75 | 0.50 | 9.75 | 6.25 | 47.00 | 4.18 | 2.00 | 2.25 | 2.50 |
| 122 | NC.QPM/DT-STR | 79.65 | 80.75 | 8.10 | 1.00 | 1.31 | 0.50 | 0.50 | 9.75 | 6.25 | 47.00 | 3.68 | 5.00 | 5.50 | 2.00 |
| 123 | ZM1421/DT-STR | 82.50 | 80.75 | 5.25 | 1.00 | 1.32 | 0.50 | 1.00 | 10.00 | 3.75 | 58.25 | 3.68 | 4.00 | 3.75 | 4.00 |
| **124** | **DTSTR-Y SYN14** | **80.13** | **79.75** | 6.62 | **1.00** | **1.36** | **0.75** | **1.50** | **11.50** | **1.75** | **93.25** | **3.68** | 1.00 | **3.75** | **3.75** |
| **125** | **ZM1423/Z.DLO** | **81.25** | **83.25** | 9.00 | **1.00** | **12.41** | **1.03** | **1.00** | **10.75** | **4.75** | **96.75** | **2.68** | 5.00 | **3.75** | **2.75** |
| **126** | **(2*TZECOMP3DT/WhiteDTSTRSYN) C2** | **77.25** | **79.25** | 9.00 | **1.00** | **1.21** | **0.91** | **1.00** | **11.25** | **1.75** | **72.50** | **2.68** | 2.50 | **4.75** | **3.75** |
