# Supplementary material for: Genetic diversity analysis of tropical and sub-tropical maize germplasm for Striga resistance and agronomic traits with SNP markers
Source: PLoS One. 2024 Aug 6;19(8):e0306263. doi: 10.1371/journal.pone.0306263 (PMC11302897; doi:10.1371/journal.pone.0306263)
Supplement: S1 File — (ZIP) [file pone.0306263.s006.zip › Supplemental Tables 1-3.docx]

**Supplemental Table 1**: List and source of maize genotypes evaluated in the present study.

| N° | Name  /designation | Source/origin | *Striga* resistance /genotype description | N° | Name  /designation | Source/origin | *Striga* resistance  /genotype description |
| --- | --- | --- | --- | --- | --- | --- | --- |
| 1 | TZISTR1154 | IITA/Nigeria | Resistant/inbred line | 29 | TZSTRI109 | IITA/Nigeria | Resistant/inbred line |
| 2 | TZISTR1261 | IITA/Nigeria | Resistant/inbred line | 30 | TZSTRI110 | IITA/Nigeria | Resistant/inbred line |
| 3 | TZISTR1248 | IITA/Nigeria | Resistant/inbred line | 31 | TZSTRI112 | IITA/Nigeria | Resistant/inbred line |
| 4 | TZISTR1263 | IITA/Nigeria | Resistant/inbred line | 32 | TZSTRI114 | IITA/Nigeria | Resistant/inbred line |
| 5 | TZISTR1275 | IITA/Nigeria | Resistant/inbred line | 33 | TZSTRI115 | IITA/Nigeria | Resistant/inbred line |
| 6 | TZISTR1157 | IITA/Nigeria | Resistant/inbred line | 34 | TZISTR25 | IITA/Nigeria | Resistant/inbred line |
| 7 | TZISTR1160 | IITA/Nigeria | Resistant/inbred line | 35 | TZISTR1001 | IITA/Nigeria | Resistant/inbred line |
| 8 | TZISTR1162 | IITA/Nigeria | Resistant/inbred line | 36 | TZISTR1003 | IITA/Nigeria | Resistant/inbred line |
| 9 | TZISTR1165 | IITA/Nigeria | Resistant/inbred line | 37 | TZISTR1004 | IITA/Nigeria | Resistant/inbred line |
| 10 | TZISTR1175 | IITA/Nigeria | Resistant/inbred line | 38 | TZISTR1008 | IITA/Nigeria | Resistant/inbred line |
| 11 | TZISTR1178 | IITA/Nigeria | Resistant/inbred line | 39 | TZISTR1011 | IITA/Nigeria | Resistant/inbred line |
| 12 | TZISTR1163 | IITA/Nigeria | Resistant/inbred line | 40 | TZISTR1018 | IITA/Nigeria | Resistant/inbred line |
| 13 | TZISTR1166 | IITA/Nigeria | Resistant/inbred line | 41 | TZEEI21 | IITA/Nigeria | Resistant/inbred line |
| 14 | TZISTR1190 | IITA/Nigeria | Resistant/inbred line | 42 | TZEEI13 | IITA/Nigeria | Resistant/inbred line |
| 15 | TZISTR1199 | IITA/Nigeria | Resistant/inbred line | 43 | TZEEI14 | IITA/Nigeria | Resistant/inbred line |
| 16 | TZISTR1231 | IITA/Nigeria | Resistant/inbred line | 44 | TZEEI49 | IITA/Nigeria | Resistant/inbred line |
| 17 | TZISTR1232 | IITA/Nigeria | Resistant/inbred line | 45 | TZDEEI55 | IITA/Nigeria | Resistant/inbred line |
| 18 | TZISTR1259 | IITA/Nigeria | Resistant/inbred line | 46 | TZDEEI50 | IITA/Nigeria | Resistant/inbred line |
| 19 | TZISTR1262 | IITA/Nigeria | Resistant/inbred line | 47 | TZEEI34 | IITA/Nigeria | Resistant/inbred line |
| 20 | TZISTR1159 | IITA/Nigeria | Resistant/inbred line | 48 | TZISTR1174 | IITA/Nigeria | Resistant/inbred line |
| 21 | TZISTR1223 | IITA/Nigeria | Resistant/inbred line | 49 | TZISTR1205 | IITA/Nigeria | Resistant/inbred line |
| 22 | TZISTR1225 | IITA/Nigeria | Resistant/inbred line | 50 | TZSTRI113 | IITA/Nigeria | Resistant/inbred line |
| 23 | TZISTR1244 | IITA/Nigeria | Resistant/inbred line | 51 | TZISTR1119 | IITA/Nigeria | Resistant/inbred line |
| 24 | TZSTRI101 | IITA/Nigeria | Resistant/inbred line | 52 | TZISTR1015 | IITA/Nigeria | Resistant/inbred line |
| 25 | TZSTRI102 | IITA/Nigeria | Resistant/inbred line | 53 | TZDEEI64 | IITA/Nigeria | Resistant/inbred line |
| 26 | TZSTRI104 | IITA/Nigeria | Resistant/inbred line | 54 | TZDEEI54 | IITA/Nigeria | Resistant/inbred line |
| 27 | TZSTRI107 | IITA/Nigeria | Resistant/inbred line | 55 | TZEEI10 | IITA/Nigeria | Resistant/inbred line |
| 28 | TZSTRI108 | IITA/Nigeria | Resistant/inbred line | 56 | CML312 | CIMMYT/Zimbabwe | Unknown/Inbred line |

**Supplemental Table 1 (**Continued)

| N° | Name  /designation | Source/origin | *Striga* resistance /breed description | N° | Name  /designation | Source/origin | *Striga* resistance /genotype description |
| --- | --- | --- | --- | --- | --- | --- | --- |
| 57 | CML444 | CIMMYT/Zimbabwe | Unknown/Inbred line | 85 | CZL1380 | CIMMYT/Zimbabwe | Unknown/Inbred line |
| 58 | CML442 | CIMMYT/Zimbabwe | Unknown/Inbred line | 86 | CLHP0326 | CIMMYT/Zimbabwe | Unknown/Inbred line |
| 59 | CML550 | CIMMYT/Zimbabwe | Unknown/Inbred line | 87 | CZL99017 | CIMMYT/Zimbabwe | Unknown/Inbred line |
| 60 | CML547 | CIMMYT/Zimbabwe | Unknown/Inbred line | 88 | CLHP0049 | CIMMYT/Zimbabwe | Unknown/Inbred line |
| 61 | CML539 | CIMMYT/Zimbabwe | Unknown/Inbred line | 89 | CLHP00478 | CIMMYT/Zimbabwe | Unknown/Inbred line |
| 62 | CML440 | CIMMYT/Zimbabwe | Unknown/Inbred line | 90 | CLHP00286 | CIMMYT/Zimbabwe | Unknown/Inbred line |
| 63 | CML566 | CIMMYT/Zimbabwe | Unknown/Inbred line | 91 | CML451 | CIMMYT/Zimbabwe | Unknown/Inbred line |
| 64 | CML540 | CIMMYT/Zimbabwe | Unknown/Inbred line | 92 | CLHP0302 | CIMMYT/Zimbabwe | Unknown/Inbred line |
| 65 | CML545 | CIMMYT/Zimbabwe | Unknown/Inbred line | 93 | CLHP0364 | CIMMYT/Zimbabwe | Unknown/Inbred line |
| 66 | CML571 | CIMMYT/Zimbabwe | Unknown/Inbred line | 94 | CLHP0350 | CIMMYT/Zimbabwe | Unknown/Inbred line |
| 67 | CML390 | CIMMYT/Zimbabwe | Unknown/Inbred line | 95 | CLHP00294 | CIMMYT/Zimbabwe | Unknown/Inbred line |
| 68 | CLHP0352 | CIMMYT/Zimbabwe | Unknown/Inbred line | 96 | CLHP0005 | CIMMYT/Zimbabwe | Unknown/Inbred line |
| 69 | HA04A-2107-36 | CIMMYT/Zimbabwe | Unknown/Inbred line | 97 | CLHP0022 | CIMMYT/Zimbabwe | Unknown/Inbred line |
| 70 | CLHP0303 | CIMMYT/Zimbabwe | Unknown/Inbred line | 98 | CML304 | CIMMYT/Zimbabwe | Unknown/Inbred line |
| 71 | CLHP0221 | CIMMYT/Zimbabwe | Unknown/Inbred line | 99 | ZM1423/Z.DLO | NPGRC/South Africa | Unknown/OPV check |
| 72 | CLHP0020 | CIMMYT/Zimbabwe | Unknown/Inbred line | 100 | NC.QPM/Z.DPLO | NPGRC/South Africa | Unknown/OPV check |
| 73 | CLHP0058 | CIMMYT/Zimbabwe | Unknown/Inbred line | 101 | M.Pearl/DT-STR | NPGRC/South Africa | Unknown/OPV check |
| 74 | CKDHL0378 | CIMMYT/Zimbabwe | Unknown/Inbred line | 102 | NC.QPM/DT-STR | NPGRC/South Africa | Unknown/OPV check |
| 75 | CLHP0312 | CIMMYT/Zimbabwe | Unknown/Inbred line | 103 | ZM1421/DT-STR | NPGRC/South Africa | Unknown/OPV check |
| 76 | CLHP0310 | CIMMYT/Zimbabwe | Unknown/Inbred line | 104 | N.Choice/1421 | NPGRC/South Africa | Unknown/hybrids check |
| 77 | CLHP0003 | CIMMYT/Zimbabwe | Unknown/Inbred line | 105 | B.King/1421 | NPGRC/South Africa | Unknown/hybrids check |
| 78 | CKDHL0467 | CIMMYT/Zimbabwe | Unknown/Inbred line | 106 | Colorado/1421 | NPGRC/South Africa | Unknown/hybrids check |
| 79 | CLHP00378 | CIMMYT/Zimbabwe | Unknown/Inbred line | 107 | Hickory/1421 | NPGRC/South Africa | Unknown/hybrids check |
| 80 | CLHP0156 | CIMMYT/Zimbabwe | Unknown/Inbred line | 108 | Kep/1421 | NPGRC/South Africa | Unknown/hybrids check |
| 81 | CLHP0113 | CIMMYT/Zimbabwe | Unknown/Inbred line | 109 | Shesha/1421 | NPGRC/South Africa | Unknown/hybrids check |
| 82 | CLHP03302 | CIMMYT/Zimbabwe | Unknown/Inbred line | 110 | ZM1423 | CIMMYT/Zimbabwe | Unknown/OPV check |
| 83 | CLHP0404 | CIMMYT/Zimbabwe | Unknown/Inbred line | 111 | ZM1421 | CIMMYT/Zimbabwe | Unknown/hybrids check |
| 84 | CLHP0343 | CIMMYT/Zimbabwe | Unknown/Inbred line | 112 | STR-SYN-Y2 | IITA/Nigeria | Resistant/OPV check |

**Supplemental Table 1** (Continued)

| N° | Name  /designation | Source/origin | *Striga* resistance /genotype description |
| --- | --- | --- | --- |
| 113 | Z.diplo-BC4-C3-W/DOGONA-1/Z.diplo-BC4-C3-W | IITA/Nigeria | Resistant/OPV check |
| 114 | Z. Diplo.BC4C3-W-DT C1 | IITA/Nigeria | Resistant/OPV check |
| 115 | TZBSTR (Susceptible)(RE) | IITA/Nigeria | Resistant/OPV check |
| 116 | STR-SYN-W1 | IITA/Nigeria | Resistant/OPV check |
| 117 | DTSTR-W SYN13 | IITA/Nigeria | Resistant/OPV check |
| 118 | DTSTR-Y SYN15 | IITA/Nigeria | Resistant/OPV check |
| 119 | ((IWD C3 SYN*2/(White DT STR Syn))-DT C1 | IITA/Nigeria | Resistant/OPV check |
| 120 | DTSTR-W SYN11 | IITA/Nigeria | Resistant/OPV check |
| 121 | SAMMMZ16 | IITA/Nigeria | Resistant/OPV check |
| 122 | (TZEOMP5C7/TZECOMP3DTC2) C2 | IITA/Nigeria | Resistant/OPV check |
| 123 | ((TZL COMP1-W C6*2/(White DT STR Syn))-DT C1 | IITA/Nigeria | Resistant/OPV check |
| 124 | TZCOM1/ZDPSYN | IITA/Nigeria | Resistant/OPV check |
| 125 | DTSTR-Y SYN14 | IITA/Nigeria | Resistant/OPV check |
| 126 | (2*TZECOMP3DT/WhiteDTSTRSYN) C2 | IITA/Nigeria | Resistant/OPV check |
| 127 | TZSTR1137/TZSTR1132 | IITA/Nigeria | Resistant/hybrid check |
| 128 | TZSTR1159/TZSTR1132 | IITA/Nigeria | Resistant/hybrid check |
| 129 | TZSTR1160/TZSTR1132 | IITA/Nigeria | Resistant/hybrid check |
| 130 | TZSTR1166/TZSTR1132 | IITA/Nigeria | Resistant/hybrid check |

**Supplemental Table 2:** Mean responses for 14 maize and *Striga* parameters assessed from 126 maize genotypes evaluated under *Striga asiatica* infestation.

| N° | Accessions | DA | DS | ASI | EPP | PLHT  (m) | EHT  (m) | HUSK  (1 to 5) | CL  (cm) | EASP  1 to 9) | GY  g/plant) | SEC8 | SEC10 | SDR8  (1 to 9) | SDR10  (1 to 9) |
| --- | --- | --- | --- | --- | --- | --- | --- | --- | --- | --- | --- | --- | --- | --- | --- |
| 1 | TZISTR1154 | 85.50 | 83.00 | -2.50 | 2.00 | 1.52 | 1.53 | 1.00 | 10.75 | 6.50 | 47.50 | 2.00 | 5.00 | 1.00 | 1.50 |
| 2 | TZISTR1261 | 87.50 | 82.00 | -5.50 | 2.00 | 1.73 | 0.85 | 1.00 | 13.75 | 4.50 | 51.25 | 2.50 | 2.00 | 2.00 | 2.00 |
| 3 | **TZISTR1248** | **78.00** | **73.50** | -4.50 | **1.00** | **1.30** | **0.59** | **1.00** | **13.25** | **3.00** | **98.25** | **3.50** | 17.50 | **4.50** | **2.00** |
| 4 | TZISTR1263 | 87.50 | 82.00 | -5.50 | 1.00 | 1.40 | 0.53 | 3.00 | 10.50 | 3.50 | 52.50 | 2.00 | 5.50 | 5.00 | 1.00 |
| 5 | TZISTR1275 | 89.50 | 76.00 | -13.50 | 1.00 | 1.60 | 0.75 | 2.00 | 14.75 | 6.00 | 54.50 | 5.00 | 5.00 | 3.00 | 2.50 |
| 6 | TZISTR1157 | 82.00 | 77.50 | -4.50 | 1.00 | 1.59 | 0.62 | 2.00 | 10.75 | 3.00 | 44.50 | 4.50 | 11.50 | 2.50 | 2.50 |
| 7 | TZISTR1160 | 84.50 | 83.50 | -1.00 | 1.00 | 1.00 | 0.60 | 1.50 | 12.50 | 7.00 | 35.00 | 5.00 | 14.50 | 3.50 | 2.00 |
| 8 | TZISTR1162 | 80.50 | 78.00 | -2.50 | 1.00 | 1.54 | 1.13 | 1.00 | 13.50 | 2.00 | 57.50 | 4.50 | 2.50 | 1.50 | 2.00 |
| 9 | TZISTR1165 | 79.50 | 75.50 | -4.00 | 2.00 | 1.85 | 1.15 | 1.00 | 11.50 | 5.00 | 32.50 | 5.00 | 16.50 | 1.50 | 1.50 |
| 10 | TZISTR1175 | 76.00 | 72.50 | -3.50 | 1.00 | 1.58 | 0.65 | 1.00 | 14.50 | 6.50 | 55.00 | 5.50 | 28.00 | 1.50 | 1.50 |
| 11 | TZISTR1178 | 76.00 | 74.50 | -1.50 | 1.00 | 2.00 | 1.55 | 1.00 | 10.15 | 1.00 | 64.55 | 5.00 | 3.50 | 3.00 | 1.50 |
| 12 | TZISTR1163 | 82.00 | 78.00 | -4.00 | 1.00 | 1.70 | 1.01 | 1.00 | 9.25 | 1.00 | 57.25 | 5.00 | 5.00 | 3.50 | 2.00 |
| 13 | TZISTR1166 | 99.00 | 101.00 | 2.00 | 1.00 | 1.00 | 0.78 | 1.00 | 10.50 | 4.00 | 62.50 | 5.50 | 7.50 | 3.00 | 2.00 |
| 14 | TZISTR1190 | 86.00 | 84.00 | -2.00 | 1.00 | 1.75 | 0.88 | 1.00 | 8.25 | 7.00 | 31.00 | 4.50 | 12.00 | 3.50 | 1.75 |
| 15 | TZISTR1199 | 81.00 | 78.50 | -2.50 | 1.00 | 1.85 | 0.90 | 1.00 | 7.75 | 4.50 | 36.00 | 5.50 | 5.00 | 3.00 | 3.00 |
| 16 | TZISTR1231 | 75.00 | 73.00 | -2.00 | 1.00 | 1.55 | 0.90 | 1.00 | 10.05 | 4.50 | 62.50 | 5.00 | 8.00 | 3.00 | 2.50 |
| 17 | TZISTR1232 | 85.50 | 86.00 | 0.50 | 1.00 | 1.75 | 1.04 | 1.00 | 11.50 | 5.00 | 35.00 | 5.50 | 15.50 | 2.50 | 2.00 |
| 18 | TZISTR1259 | 77.00 | 74.00 | -3.00 | 1.00 | 1.40 | 0.76 | 1.00 | 15.50 | 5.50 | 43.25 | 5.00 | 10.00 | 4.50 | 1.50 |
| 19 | TZISTR1262 | 83.50 | 74.50 | -9.00 | 1.00 | 1.65 | 0.82 | 2.00 | 11.50 | 6.50 | 0.00 | 4.50 | 16.50 | 3.50 | 2.00 |
| 20 | TZISTR1159 | 86.50 | 71.00 | -15.50 | 1.00 | 1.90 | 0.97 | 1.00 | 9.75 | 4.50 | 29.25 | 5.50 | 8.00 | 1.50 | 1.50 |
| 21 | TZISTR1223 | 87.00 | 83.50 | -3.50 | 1.00 | 1.95 | 1.30 | 1.00 | 9.50 | 3.50 | 60.00 | 4.50 | 8.00 | 2.00 | 1.50 |
| 22 | TZISTR1225 | 85.50 | 84.50 | -1.00 | 1.00 | 1.90 | 1.45 | 1.00 | 10.50 | 3.00 | 62.50 | 4.50 | 4.50 | 1.00 | 2.00 |
| 23 | CML550 | 85.50 | 85.00 | -0.50 | 1.00 | 2.03 | 1.15 | 1.00 | 12.50 | 6.50 | 28.75 | 4.50 | 11.50 | 3.50 | 3.50 |
| 24 | TZISTR1244 | 76.00 | 76.50 | 0.50 | 1.00 | 1.00 | 0.45 | 2.00 | 9.00 | 3.50 | 47.50 | 5.00 | 7.50 | 3.00 | 3.50 |
| 25 | TZSTRI101 | 99.00 | 98.50 | -0.50 | 1.00 | 1.00 | 0.55 | 1.00 | 9.00 | 3.50 | 52.50 | 5.00 | 14.00 | 3.00 | 3.50 |
| 26 | TZSTRI102 | 87.50 | 78.00 | -9.50 | 1.00 | 1.85 | 0.85 | 1.00 | 10.50 | 1.50 | 52.25 | 5.00 | 15.50 | 4.00 | 2.50 |
| 27 | TZSTRI104 | 100.00 | 99.00 | -1.00 | 1.00 | 1.00 | 0.45 | 1.50 | 10.50 | 3.50 | 57.50 | 5.00 | 23.00 | 3.00 | 4.00 |
| 28 | TZSTRI107 | 77.50 | 73.50 | -4.00 | 1.00 | 1.40 | 0.84 | 1.00 | 8.50 | 2.00 | 57.00 | 5.00 | 1.50 | 1.50 | 2.50 |
| 29 | TZSTRI108 | 81.00 | 80.00 | -1.00 | 1.00 | 1.40 | 0.77 | 2.00 | 9.00 | 2.00 | 28.00 | 5.00 | 4.50 | 5.50 | 3.00 |
| 30 | TZSTRI109 | 80.00 | 73.00 | -7.00 | 2.00 | 2.03 | 0.97 | 1.00 | 11.00 | 5.00 | 57.25 | 4.50 | 2.50 | 2.50 | 2.00 |
| 31 | TZSTRI110 | 82.00 | 74.50 | -7.50 | 1.00 | 2.03 | 1.45 | 1.00 | 7.75 | 5.00 | 37.00 | 4.50 | 29.50 | 2.00 | 2.50 |
| 32 | TZSTRI112 | 81.00 | 76.50 | -4.50 | 1.00 | 1.08 | 0.80 | 1.00 | 12.50 | 5.00 | 68.75 | 4.50 | 4.50 | 3.00 | 3.50 |
| 33 | TZSTRI114 | 93.00 | 93.50 | 0.50 | 1.00 | 1.05 | 0.55 | 1.00 | 10.50 | 4.50 | 65.00 | 5.00 | 19.00 | 3.00 | 3.50 |
| 34 | **TZSTRI115** | **77.50** | **77.00** | -0.50 | **1.00** | **2.10** | **1.20** | **1.00** | **11.50** | **1.50** | **112.50** | **5.00** | 2.00 | **3.50** | **2.50** |
| 35 | **TZISTR25** | **76.50** | **72.50** | -4.00 | **1.00** | **2.25** | **1.13** | **1.00** | **13.50** | **2.00** | **97.25** | **5.00** | 4.50 | **3.00** | **3.50** |
| 36 | **TZISTR1001** | **82.00** | **82.00** | 0.00 | **1.00** | **2.10** | **1.28** | **1.00** | **11.00** | **1.50** | **140.00** | **4.50** | 4.50 | **3.00** | **2.50** |
| 37 | TZISTR1003 | 84.50 | 80.00 | -4.50 | 1.00 | 2.05 | 1.42 | 1.00 | 8.75 | 2.00 | 44.50 | 4.50 | 32.00 | 3.00 | 3.50 |
| 38 | TZISTR1004 | 70.50 | 72.50 | 2.00 | 1.00 | 1.58 | 1.13 | 1.00 | 9.25 | 1.50 | 54.50 | 4.50 | 4.50 | 2.00 | 4.00 |
| 39 | TZISTR1008 | 85.50 | 83.50 | -2.00 | 1.00 | 2.00 | 1.13 | 1.00 | 11.00 | 5.50 | 38.75 | 5.00 | 9.50 | 4.50 | 3.00 |
| 40 | TZISTR1011 | 91.00 | 91.50 | 0.50 | 1.00 | 1.80 | 1.15 | 1.00 | 10.75 | 2.50 | 58.00 | 4.50 | 12.50 | 4.50 | 3.50 |
| 41 | TZISTR1018 | 75.50 | 74.50 | -1.00 | 1.00 | 1.70 | 1.15 | 1.00 | 12.00 | 3.50 | 47.75 | 5.00 | 8.00 | 1.00 | 1.00 |
| 42 | TZEEI21 | 81.50 | 82.00 | 0.50 | 1.00 | 1.18 | 0.80 | 1.00 | 12.50 | 3.00 | 67.50 | 4.50 | 2.50 | 4.50 | 3.50 |
| 43 | TZEEI13 | 77.00 | 76.00 | -1.00 | 1.00 | 1.30 | 0.72 | 2.00 | 8.50 | 3.00 | 58.25 | 5.50 | 5.50 | 3.50 | 5.50 |
| 44 | TZEEI14 | 82.00 | 82.00 | 0.00 | 1.00 | 2.10 | 1.13 | 2.00 | 10.25 | 1.50 | 63.00 | 4.50 | 9.50 | 4.50 | 3.00 |
| 45 | TZEEI49 | 72.50 | 74.50 | 2.00 | 1.00 | 1.97 | 0.77 | 2.00 | 11.25 | 3.00 | 36.00 | 4.50 | 7.00 | 5.00 | 5.00 |
| 46 | TZDEEI55 | 76.50 | 75.50 | -1.00 | 1.00 | 1.59 | 0.61 | 2.00 | 8.50 | 4.00 | 36.25 | 5.00 | 2.00 | 3.50 | 5.00 |
| 47 | TZDEEI50 | 70.00 | 72.50 | 2.50 | 1.00 | 1.42 | 0.75 | 2.00 | 10.50 | 1.50 | 69.25 | 4.00 | 2.00 | 5.50 | 4.50 |
| 48 | TZDEEI64 | 69.50 | 72.50 | 3.00 | 1.00 | 1.74 | 1.25 | 2.00 | 11.00 | 3.50 | 57.50 | 4.50 | 3.50 | 3.50 | 5.00 |
| 49 | **CML312** | **80.50** | **85.00** | 4.50 | **1.00** | **2.05** | **0.91** | **2.00** | **8.75** | **2.00** | **86.25** | **4.50** | 5.00 | **4.50** | **3.00** |
| 50 | CML444 | 78.50 | 81.00 | 2.50 | 1.00 | 1.50 | 0.67 | 2.00 | 12.50 | 3.00 | 31.75 | 4.50 | 9.50 | 5.00 | 4.00 |
| 51 | CML442 | 85.50 | 86.00 | 0.50 | 1.00 | 1.69 | 0.80 | 2.00 | 11.50 | 1.50 | 55.00 | 4.50 | 3.00 | 4.50 | 3.50 |
| 52 | TZDEEI54 | 90.50 | 91.00 | 0.50 | 1.00 | 1.00 | 0.75 | 2.00 | 10.50 | 3.50 | 47.50 | 5.50 | 6.50 | 5.00 | 2.00 |
| 53 | TZEEI10 | 86.50 | 84.50 | -2.00 | 1.00 | 1.65 | 0.92 | 2.00 | 12.75 | 6.50 | 26.00 | 4.50 | 3.00 | 3.50 | 3.50 |
| 54 | CML547 | 83.50 | 90.50 | 7.00 | 1.00 | 1.45 | 0.60 | 1.50 | 9.50 | 3.50 | 62.50 | 4.00 | 3.50 | 6.00 | 2.50 |
| 55 | **CML539** | **80.50** | **74.50** | -6.00 | **1.00** | **1.85** | **0.60** | **1.00** | **10.50** | **3.00** | **74.75** | **5.00** | 5.00 | **4.50** | **3.00** |
| 56 | **CML440** | **82.50** | **76.00** | -6.50 | **1.00** | **2.36** | **1.08** | **1.00** | **11.00** | **1.50** | **96.25** | **4.50** | 13.50 | **3.00** | **1.50** |
| 57 | **CML566** | **82.50** | **79.00** | -3.50 | **1.00** | **2.22** | **1.15** | **1.00** | **12.00** | **1.50** | **155.50** | **4.00** | 5.50 | **1.50** | **1.50** |
| 58 | **CML540** | **77.00** | **81.50** | 4.50 | **1.00** | **2.03** | **0.77** | **1.00** | **11.00** | **3.50** | **277.50** | **4.00** | 1.50 | **3.50** | **3.00** |
| 59 | CML545 | 75.50 | 74.00 | -1.50 | 1.00 | 1.73 | 0.67 | 1.00 | 10.25 | 3.00 | 53.00 | 4.50 | 6.00 | 5.00 | 3.00 |
| 60 | CML571 | 76.00 | 75.50 | -0.50 | 1.00 | 2.05 | 0.68 | 1.00 | 9.80 | 2.00 | 45.50 | 4.00 | 26.50 | 5.00 | 4.00 |
| 61 | CML390 | 80.00 | 81.00 | 1.00 | 1.00 | 2.10 | 0.74 | 1.00 | 11.30 | 2.50 | 56.00 | 4.50 | 7.00 | 5.00 | 3.50 |
| 62 | CLHP0352 | 76.50 | 76.50 | 0.00 | 1.00 | 2.30 | 0.85 | 1.00 | 11.75 | 3.00 | 51.25 | 4.50 | 11.50 | 5.00 | 3.00 |
| 63 | HA04A-2107-36 | 97.50 | 86.00 | -11.50 | 1.00 | 2.14 | 1.35 | 1.00 | 8.50 | 5.50 | 27.50 | 4.50 | 8.00 | 4.50 | 4.50 |
| 64 | **CLHP0303** | **84.50** | **83.50** | -1.00 | **1.50** | **1.87** | **1.15** | **1.00** | **7.25** | **3.00** | **92.50** | **4.50** | 8.50 | **3.00** | **3.00** |
| 65 | **CLHP0221** | **79.50** | **77.50** | -2.00 | **1.50** | **1.65** | **1.10** | **1.00** | **10.25** | **5.50** | **72.50** | **5.00** | 9.50 | **3.50** | **3.00** |
| 66 | **CLHP0020** | **71.00** | **77.00** | 6.00 | **1.00** | **1.80** | **0.57** | **1.00** | **12.00** | **2.50** | **119.00** | **4.50** | 2.00 | **3.50** | **3.50** |
| 67 | CLHP0058 | 78.50 | 76.50 | -2.00 | 1.00 | 1.40 | 0.46 | 1.00 | 8.50 | 4.50 | 26.00 | 5.00 | 3.00 | 5.50 | 2.50 |
| 68 | **CKDHL0378** | **72.00** | **75.00** | 3.00 | **1.00** | **1.90** | **0.75** | **1.00** | **11.75** | **2.00** | **85.00** | **4.50** | 17.50 | **3.00** | **2.50** |
| 69 | CLHP0312 | 80.50 | 79.50 | -1.00 | 1.00 | 1.75 | 0.76 | 1.00 | 9.50 | 2.00 | 45.75 | 4.50 | 18.50 | 4.50 | 3.50 |
| 70 | CLHP0310 | 76.00 | 76.50 | 0.50 | 1.00 | 1.91 | 1.03 | 1.00 | 11.75 | 4.00 | 35.75 | 5.00 | 13.50 | 5.00 | 3.50 |
| 71 | CLHP0003 | 82.00 | 83.00 | 1.00 | 1.00 | 1.40 | 0.82 | 1.00 | 9.00 | 5.50 | 30.00 | 6.00 | 4.50 | 2.50 | 3.00 |
| 72 | CKDHL0467 | 79.50 | 75.50 | -4.00 | 1.00 | 1.65 | 0.86 | 1.00 | 11.25 | 4.00 | 42.75 | 4.50 | 14.50 | 2.50 | 3.50 |
| 73 | CLHP00378 | 77.00 | 79.00 | 2.00 | 1.00 | 1.03 | 0.60 | 2.50 | 9.50 | 4.00 | 57.50 | 6.00 | 3.00 | 3.00 | 3.00 |
| 74 | **CLHP0156** | **80.50** | **78.50** | -2.00 | **1.50** | **2.40** | **0.98** | **1.00** | **12.00** | **1.50** | **74.50** | **5.00** | 8.00 | **1.50** | **2.00** |
| 75 | CLHP0113 | 77.50 | 74.50 | -3.00 | 1.00 | 1.65 | 0.50 | 2.50 | 10.00 | 5.00 | 30.50 | 5.00 | 4.00 | 3.50 | 3.50 |
| 76 | **CLHP03302** | **75.50** | **70.50** | -5.00 | **1.50** | **1.80** | **0.95** | **1.00** | **12.00** | **2.00** | **75.25** | **5.50** | 4.00 | **2.00** | **1.00** |
| 77 | CLHP0404 | 77.00 | 75.50 | -1.50 | 1.00 | 1.68 | 0.70 | 1.00 | 11.00 | 3.50 | 62.50 | 5.50 | 8.00 | 3.00 | 3.00 |
| 78 | **CLHP0343** | **77.50** | **77.00** | -0.50 | **1.00** | **1.60** | **0.77** | **1.00** | **11.50** | **1.00** | **82.00** | **4.50** | 2.00 | **4.00** | **4.50** |
| 79 | CZL1380 | 78.50 | 78.50 | 0.00 | 1.00 | 2.38 | 0.87 | 1.00 | 10.50 | 5.50 | 62.50 | 5.00 | 13.50 | 5.00 | 5.00 |
| 80 | **CLHP0326** | **75.00** | **77.00** | 2.00 | **1.00** | **1.40** | **0.66** | **1.00** | **10.50** | **2.00** | **82.25** | **4.50** | 2.00 | **3.00** | **2.50** |
| 81 | CZL99017 | 79.50 | 79.00 | -0.50 | 1.00 | 2.03 | 0.77 | 1.00 | 8.50 | 2.50 | 62.50 | 5.00 | 8.50 | 3.00 | 2.50 |
| 82 | TZEEI34 | 74.50 | 74.50 | 0.00 | 1.00 | 1.30 | 0.53 | 1.00 | 10.50 | 4.50 | 36.50 | 4.50 | 3.50 | 4.00 | 1.50 |
| 83 | **CLHP0049** | **80.50** | **78.00** | -2.50 | **1.00** | **1.25** | **0.70** | **1.00** | **10.00** | **3.00** | **101.25** | **7.00** | 4.00 | **4.00** | **2.50** |
| 84 | CLHP00478 | 79.50 | 74.50 | -5.00 | 1.00 | 1.73 | 0.70 | 1.00 | 11.50 | 3.00 | 56.00 | 5.00 | 20.50 | 3.50 | 3.50 |
| 85 | **CLHP00286** | **83.00** | **83.00** | 0.00 | **1.00** | **1.88** | **0.88** | **1.00** | **11.50** | **3.50** | **82.50** | **5.50** | 3.50 | **3.00** | **3.50** |
| 86 | **CML451** | **79.00** | **79.00** | 0.00 | **1.00** | **2.30** | **0.96** | **1.00** | **12.00** | **1.50** | **83.00** | **4.50** | 10.50 | **2.50** | **3.50** |
| 87 | **CLHP0302** | **81.00** | **80.50** | -0.50 | **1.00** | **1.76** | **1.00** | **3.00** | **13.25** | **3.00** | **98.00** | **4.50** | 7.00 | **5.00** | **3.50** |
| 88 | **CLHP0364** | **80.00** | **80.50** | 0.50 | **1.00** | **1.30** | **0.85** | **1.00** | **12.00** | **3.00** | **79.00** | **4.50** | 4.00 | **2.00** | **5.00** |
| 89 | **CLHP0350** | **75.00** | **76.00** | 1.00 | **1.00** | **2.35** | **0.81** | **3.00** | **14.00** | **3.50** | **102.75** | **5.00** | 3.50 | **2.00** | **3.50** |
| 90 | CLHP00294 | 88.00 | 87.50 | -0.50 | 1.00 | 2.00 | 0.75 | 1.00 | 9.50 | 5.50 | 40.00 | 5.00 | 1.50 | 5.00 | 5.00 |
| 91 | CLHP0005 | 76.00 | 77.50 | 1.50 | 1.00 | 2.03 | 0.76 | 1.00 | 10.50 | 3.50 | 58.75 | 4.50 | 19.00 | 2.00 | 4.50 |
| 92 | CLHP0022 | 81.00 | 81.50 | 0.50 | 1.00 | 1.50 | 0.55 | 1.00 | 7.00 | 4.00 | 12.50 | 5.50 | 3.00 | 3.00 | 5.50 |
| 93 | **CML304** | 82.50 | 82.50 | 0.00 | 1.00 | 1.75 | 0.95 | 2.00 | 11.00 | 4.00 | 59.25 | 6.50 | 33.50 | 4.50 | 6.50 |
| 94 | **TZISTR1174** | **84.00** | **82.50** | -1.50 | **1.00** | **1.80** | **1.00** | **1.50** | **12.25** | **2.00** | **93.25** | **3.00** | 47.00 | **3.00** | **3.50** |
| 95 | **TZISTR1205** | **81.50** | **75.50** | -6.00 | **1.00** | **1.85** | **0.91** | **1.00** | **11.00** | **1.00** | **114.25** | **3.50** | 13.00 | **3.00** | **4.50** |
| 96 | **TZSTRI113** | **78.00** | **77.00** | -1.00 | **1.00** | **1.75** | **0.95** | **1.00** | **10.50** | **1.50** | **87.00** | **3.00** | 7.50 | **1.50** | **6.00** |
| 97 | **TZISTR1119** | **84.00** | **80.50** | -3.50 | **1.00** | **1.91** | **1.15** | **1.00** | **11.25** | **4.00** | **84.75** | **3.00** | 19.00 | **2.50** | **4.50** |
| 98 | **TZISTR1015** | **81.00** | **77.00** | -4.00 | **1.00** | **1.77** | **0.85** | **1.00** | **10.50** | **1.00** | **73.25** | **2.50** | 5.50 | **3.50** | **4.00** |
| 99 | **ZM1421** | **80.50** | **81.00** | 0.50 | **1.00** | **1.00** | **0.70** | **1.00** | **9.00** | **3.50** | **77.50** | **5.00** | 4.50 | **1.00** | **2.00** |
| 100 | **B.King/1421** | **80.50** | **78.50** | -2.00 | **1.00** | **2.05** | **1.15** | **2.00** | **23.50** | **1.50** | **157.25** | **5.00** | 4.50 | **1.00** | **2.00** |
| 101 | Hickory/1421 | 70.50 | 69.50 | -1.00 | 1.00 | 1.95 | 1.30 | 2.00 | 15.50 | 5.00 | 0.00 | 4.50 | 27.00 | 3.00 | 3.00 |
| 102 | Kep/1421 | 69.50 | 73.00 | 3.50 | 1.00 | 2.30 | 1.75 | 1.00 | 17.50 | 5.00 | 25.75 | 4.50 | 2.00 | 2.50 | 1.50 |
| 103 | **Shesha/1421** | **75.50** | **72.50** | -3.00 | **1.00** | **2.03** | **1.75** | **1.00** | **18.75** | **1.50** | **165.75** | **7.50** | 18.50 | **2.00** | **2.00** |
| 104 | **ZM1423** | **69.00** | **69.50** | 0.50 | **1.00** | **0.85** | **1.39** | **1.00** | **10.50** | **1.50** | **99.25** | **4.50** | 16.50 | **5.00** | **3.00** |
| 105 | **N.Choice/1421** | **82.00** | **76.50** | -5.50 | **1.00** | **1.90** | **1.03** | **1.00** | **13.25** | **1.50** | **214.00** | **5.00** | 4.00 | **3.50** | **3.50** |
| 106 | NC.QPM/Z.DPLO | **79.50** | **74.50** | -5.00 | **1.00** | **2.05** | **0.88** | **1.00** | **15.50** | **1.50** | **169.50** | **4.50** | 3.50 | **2.50** | **3.00** |
| 107 | STR-SYN-Y2 | 81.50 | 82.00 | 0.50 | 1.00 | 2.40 | 0.98 | 1.00 | 11.50 | 6.00 | 44.50 | 6.00 | 2.50 | 3.50 | 5.50 |
| 108 | Z. Diplo.BC4C3-W-DT C1 | 88.00 | 88.50 | 0.50 | 1.00 | 2.45 | 0.95 | 1.50 | 9.00 | 5.50 | 42.50 | 6.00 | 2.00 | 4.00 | 5.00 |
| 109 | **TZBSTR (Susceptible)** | **83.00** | **83.50** | 0.50 | **1.00** | **2.65** | **1.30** | **1.50** | **14.50** | **1.00** | **103.00** | **6.50** | 3.00 | **3.00** | **2.50** |
| 110 | STR-SYN-W1 | 80.00 | 79.50 | -0.50 | 1.00 | 2.55 | 1.18 | 1.50 | 13.50 | 4.00 | 53.50 | 6.50 | 14.50 | 3.50 | 4.00 |
| 111 | **DTSTR-W SYN13** | **85.50** | **85.50** | 0.00 | **1.00** | **1.25** | **0.85** | **1.50** | **13.00** | **3.50** | **107.50** | **4.50** | 3.50 | **1.50** | **5.00** |
| 112 | DTSTR-Y SYN15 | 77.00 | 72.50 | -4.50 | 1.00 | 2.80 | 1.30 | 1.00 | 12.75 | 4.00 | 57.50 | 4.50 | 2.50 | 2.00 | 3.00 |
| 113 | ((IWD C3 SYN*2/(White DT STR Syn))-DT C1 | 89.00 | 91.50 | 2.50 | 1.00 | 2.45 | 0.75 | 1.50 | 11.50 | 4.00 | 35.00 | 5.50 | 3.50 | 3.50 | 6.00 |
| 114 | **DTSTR-W SYN11** | **78.50** | **77.50** | -1.00 | **1.00** | **2.30** | **1.23** | **1.50** | **13.00** | **2.50** | **71.25** | **5.00** | 52.50 | **3.50** | **5.50** |
| 115 | SAMMMZ16 | 86.50 | 87.00 | 0.50 | 1.00 | 2.50 | 1.10 | 1.00 | 11.75 | 3.00 | 54.25 | 5.00 | 11.00 | 4.50 | 4.50 |
| 116 | **(TZEOMP5C7/TZECOMP3DTC2) C2** | **75.00** | **75.50** | 0.50 | **1.00** | **2.38** | **1.10** | **1.50** | **14.00** | **1.00** | **89.50** | **4.00** | 32.00 | **4.00** | **5.00** |
| 117 | ((TZL COMP1-W C6*2/(White DT STR Syn))-DT C1 | 85.00 | 86.50 | 1.50 | 1.00 | 1.40 | 0.85 | 1.00 | 11.00 | 3.50 | 62.50 | 3.00 | 2.50 | 2.50 | 3.00 |
| 118 | TZCOM1/ZDPSYN | 74.00 | 69.50 | -4.50 | 1.00 | 2.15 | 1.02 | 1.00 | 15.00 | 3.50 | 61.25 | 3.00 | 10.00 | 3.00 | 4.00 |
| 119 | **Colorado/1421** | **87.50** | **89.50** | 2.00 | **1.00** | **2.03** | **0.70** | **1.00** | **13.75** | **2.00** | **75.25** | **2.50** | 3.00 | **3.50** | **5.00** |
| 120 | M.Pearl/DT-STR | 73.50 | 74.00 | 0.50 | 1.00 | 2.38 | 1.04 | 0.50 | 13.50 | 3.50 | 61.50 | 2.00 | 17.00 | 2.50 | 3.00 |
| 121 | **Z.diplo-BC4-C3-W/DOGONA-1/Z.diplo-BC4-C3-W** | **81.00** | **83.00** | 2.00 | **1.00** | **2.36** | **1.05** | **1.00** | **11.50** | **1.50** | **112.00** | **6.00** | 8.50 | **3.00** | **3.50** |
| 122 | NC.QPM/DT-STR | 71.50 | 71.50 | 0.00 | 1.00 | 2.25 | 1.03 | 0.00 | 12.50 | 4.50 | 54.25 | 3.00 | 5.00 | 3.50 | 5.00 |
| 123 | **ZM1421/DT-STR** | **77.00** | **76.50** | -0.50 | **1.00** | **2.38** | **1.10** | **1.50** | **10.75** | **3.00** | **93.50** | **2.50** | 19.00 | **2.50** | **6.00** |
| 124 | **DTSTR-Y SYN14** | **78.50** | **74.50** | -4.00 | **1.00** | **2.23** | **1.10** | **0.50** | **13.75** | **5.00** | **78.50** | **2.50** | 8.50 | **3.50** | **3.50** |
| 125 | **(2*TZECOMP3DT/WhiteDTSTRSYN) C2** | **69.00** | **78.00** | 9.00 | **1.00** | **1.75** | **0.85** | **1.50** | **12.25** | **2.50** | **89.00** | **6.00** | 0.50 | **2.00** | **5.50** |
| 126 | ZM1423/Z.DLO | 84.50 | 85.00 | 0.50 | 1.00 | 2.50 | 1.05 | 1.50 | 13.25 | 2.00 | 64.25 | 3.50 | 6.00 | 2.50 | 4.00 |
|  |  | **DA** | **DS** | **ASI** | **EPP** | **PLHT** | **EHT** | **HUSK** | **CL** | **EASP** | **GY** | **SEC8** | **SEC10** | **SDR8** | **SDR10** |
|  | **Trial Statistic** | **Inbred lines** | | | | | | | | | | | | | |
|  | Minimum | 69.50 | 70.50 | 0.00 | 1.00 | 1.00 | 0.45 | 1.00 | 7.00 | 1.00 | 0.00 | 2.00 | 1.50 | 1.00 | 1.00 |
|  | Maximum | 100.00 | 101.00 | 15.50 | 2.00 | 2.40 | 1.55 | 3.00 | 15.50 | 7.00 | 277.50 | 7.00 | 47.00 | 6.00 | 6.50 |
|  | Mean | 81.14 | 79.44 | 2.77 | 1.06 | 1.71 | 0.88 | 1.28 | 10.79 | 3.42 | 62.77 | 5.00 | 45.50 | 3.35 | 3.07 |
| Open-pollinated varieties checks | | | | | | | | | | | | | | | |
|  | Minimum | 69.00 | 69.50 | 0.00 | 1.00 | 0.85 | 0.70 | 0.50 | 9.00 | 1.00 | 35.00 | 2.00 | 0.50 | 1.50 | 2.50 |
|  | Maximum | 89.00 | 91.50 | 9.00 | **1.00** | 2.80 | 1.39 | 1.50 | 15.50 | 6.00 | 169.50 | 6.50 | 52.50 | 5.00 | 6.00 |
|  | Mean | 79.73 | 79.77 | 1.86 | **1.00** | 2.18 | 1.04 | 1.18 | 12.63 | 3.11 | 76.33 | 4.39 | 10.32 | 3.14 | 4.30 |
|  | **Hybrids checks** | | | | | | | | | | | | | | |
|  | Minimum | 69.00 | 69.50 | 0.5 | 1.00 | 0.85 | 0.70 | 0.50 | 9.00 | 1.00 | 00.00 | 2.00 | 0.50 | 1.50 | 2.50 |
|  | Maximum | 87.50 | 89.50 | 5.50 | 1.00 | 2.30 | 0.70 | 2.00 | 23.50 | 5.00 | 214.00 | 7.50 | 27.00 | 3.50 | 5.00 |
|  | Mean | 78.00 | 77.21 | 1.77 | 1.00 | 1.89 | 1.20 | 1.29 | 15.89 | 2.86 | 102.21 | 4.86 | 9.07 | 2.36 | 2.71 |

DA= days to 50% anthesis, DS= days to 50% silking, ASI= anthesis-silking interval, EPP = ear per plant, PLHT= Plant height, EHT= ear height, HUSK= husk cover, CL= cob length, EASP= ear aspect, GY= grain yield, SEC8= *Striga* emergence counts eight weeks after sowing, SEC10= *Striga* emergence counts ten weeks after sowing, SDR8= *Striga* damage rating eight weeks after sowing, and SDR10= *Striga* damage rating 10 weeks after sowing, m= meter, cm= centimetre, g= gramme. Boldface entries denote best-performing genotypes.

**Supplemental Table 3:** Mean responses for 14 maize and *Striga* parameters assessed from 126 maize genotypes evaluated under *Striga hermonthica* infestation

| N° | Accessions | DA | DS | ASI | EPP | PLHT  (m) | EHT  (m) | HUSK  (1 to 5) | CL  (cm) | EASP  (1 to 9) | GY  (g/plant) | SEC8 | SEC10 | SDR8  (1 to 9) | SDR10  (1 to 9) | |
| --- | --- | --- | --- | --- | --- | --- | --- | --- | --- | --- | --- | --- | --- | --- | --- | --- |
| 1 | TZISTR1154 | 78.50 | 79.50 | 1.00 | 1.50 | 2.25 | 1.25 | 1.00 | 11.00 | 3.00 | 67.25 | 2.50 | 1.50 | 3.00 | 2.25 | |
| 2 | **TZISTR1261** | **78.63** | **80.63** | 2.00 | **2.00** | **2.20** | **0.97** | **2.00** | **10.96** | **3.25** | **70.25** | **3.75** | 4.50 | **2.00** | **1.75** | |
| 3 | **TZISTR1248** | **75.88** | **77.25** | 1.37 | **1.50** | **1.75** | **0.77** | **2.00** | **11.96** | **2.25** | **81.25** | **4.75** | 1.50 | **2.25** | **2.00** | |
| 4 | **TZISTR1263** | **76.25** | **78.25** | 2.00 | **1.00** | **1.35** | **0.59** | **1.00** | **10.96** | **3.75** | **76.00** | **3.25** | 9.00 | **3.75** | **2.75** | |
| 5 | TZISTR1275 | 82.00 | 83.00 | 1.00 | 1.00 | 1.50 | 0.49 | 1.00 | 9.96 | 5.25 | 65.25 | 3.25 | 6.00 | 4.50 | 3.50 | |
| 6 | TZISTR1157 | 81.13 | 81.25 | 0.12 | 1.00 | 1.55 | 0.54 | 2.00 | 9.96 | 5.25 | 65.25 | 3.75 | 3.50 | 2.25 | 3.50 | |
| 7 | TZISTR1160 | 79.88 | 80.50 | 0.62 | 1.00 | 1.47 | 0.54 | 2.00 | 10.46 | 3.75 | 57.25 | 2.75 | 6.50 | 3.75 | 2.75 | |
| 8 | TZISTR1162 | 81.70 | 84.13 | 2.43 | 1.00 | 1.70 | 0.71 | 1.00 | 12.21 | 2.25 | 63.75 | 2.25 | 3.00 | 3.00 | 3.00 | |
| 9 | TZISTR1165 | 82.38 | 81.75 | -0.63 | 1.00 | 2.10 | 0.99 | 1.00 | 13.46 | 2.75 | 66.25 | 3.25 | 3.50 | 2.75 | 3.00 | |
| 10 | TZISTR1175 | 79.00 | 78.25 | -0.75 | 1.00 | 2.10 | 0.92 | 1.00 | 11.71 | 5.75 | 59.75 | 3.75 | 5.50 | 2.75 | 1.75 | |
| 11 | TZISTR1178 | 74.50 | 74.00 | -0.50 | 1.00 | 2.35 | 0.94 | 1.00 | 9.96 | 6.75 | 65.25 | 3.25 | 3.50 | 3.75 | 1.75 | |
| 12 | TZISTR1163 | 76.75 | 77.50 | 0.75 | 1.00 | 2.25 | 1.07 | 1.00 | 9.46 | 3.25 | 65.00 | 3.25 | 1.00 | 3.50 | 3.00 | |
| 13 | TZISTR1166 | 90.00 | 87.50 | -2.50 | 1.00 | 1.60 | 0.77 | 1.00 | 9.46 | 3.25 | 65.00 | 3.25 | 6.50 | 2.75 | 2.00 | |
| 14 | TZISTR1190 | 93.50 | 92.00 | -1.50 | 1.00 | 1.45 | 0.69 | 1.50 | 8.46 | 3.25 | 67.00 | 3.75 | 5.00 | 4.25 | 2.25 | |
| 15 | TZISTR1199 | 82.75 | 81.00 | -1.75 | 1.00 | 1.85 | 0.97 | 1.50 | 8.46 | 3.25 | 64.25 | 3.25 | 2.00 | 3.25 | 2.75 | |
| 16 | TZISTR1231 | 73.25 | 72.25 | -1.00 | 1.00 | 1.85 | 0.94 | 1.00 | 9.21 | 7.25 | 62.50 | 2.75 | 6.50 | 3.75 | 2.25 | |
| 17 | TZISTR1232 | 77.50 | 76.75 | -0.75 | 1.00 | 1.83 | 0.94 | 1.00 | 10.71 | 8.75 | 46.75 | 3.25 | 2.00 | 2.00 | 2.00 | |
| 18 | TZISTR1259 | 77.75 | 78.75 | 1.00 | 1.00 | 1.78 | 0.89 | 2.00 | 13.46 | 8.25 | 25.75 | 3.25 | 3.50 | 3.00 | 2.25 | |
| 19 | TZISTR1262 | 76.75 | 74.50 | -2.25 | 1.00 | 1.65 | 0.73 | 2.50 | 10.71 | 5.75 | 39.25 | 2.63 | 5.00 | 3.75 | 3.00 | |
| 20 | TZISTR1159 | 76.50 | 72.75 | -3.75 | 1.00 | 1.85 | 0.86 | 1.50 | 8.46 | 5.25 | 46.25 | 3.13 | 2.00 | 1.75 | 2.75 | |
| 21 | TZISTR1223 | 80.88 | 78.00 | -2.88 | 1.00 | 2.10 | 0.97 | 1.00 | 9.46 | 5.25 | 55.00 | 3.13 | 2.50 | 3.25 | 2.75 | |
| 22 | TZISTR1225 | 84.75 | 83.25 | -1.50 | 1.00 | 2.00 | 0.88 | 1.00 | 8.71 | 3.25 | 65.25 | 2.63 | 4.50 | 3.25 | 3.75 | |
| 23 | CML550 | **73.25** | **73.25** | 0.00 | **1.00** | **2.13** | **1.00** | **1.00** | **13.21** | **1.25** | **86.00** | **2.63** | 5.00 | **1.50** | **2.00** | |
| 24 | TZISTR1244 | 79.25 | 75.25 | -4.00 | 1.00 | 1.55 | 0.82 | 1.50 | 8.46 | 1.75 | 48.25 | 2.63 | 7.00 | 2.25 | 2.00 | |
| 25 | TZSTRI101 | **90.00** | **87.50** | -2.50 | **1.00** | **1.45** | **0.75** | **1.00** | **12.46** | **3.25** | **144.00** | **3.63** | 6.50 | **3.75** | **3.00** | |
| 26 | TZSTRI102 | 87.75 | 84.75 | -3.00 | 1.00 | 1.45 | 0.75 | 1.00 | 12.96 | 5.25 | 51.75 | 3.13 | 4.50 | 3.00 | 2.00 | |
| 27 | TZSTRI104 | 89.50 | 90.75 | 1.25 | 1.00 | 1.45 | 0.75 | 1.00 | 12.96 | 5.25 | 51.75 | 3.13 | 7.50 | 3.50 | 1.75 | |
| 28 | **TZSTRI107** | **88.00** | **86.75** | -1.25 | **1.00** | **1.25** | **0.66** | **1.00** | **8.75** | **3.25** | **70.25** | **3.63** | 8.00 | **2.25** | **2.50** | |
| 29 | TZSTRI108 | **77.25** | **75.00** | -2.25 | **1.00** | **1.55** | **0.88** | **1.00** | **8.75** | **1.25** | **77.00** | **3.13** | 1.50 | **3.50** | **2.50** | |
| 30 | TZSTRI109 | **80.75** | **78.00** | -2.75 | **1.00** | **1.75** | **0.97** | **1.00** | **11.00** | **2.25** | **78.00** | **3.13** | 4.50 | **3.75** | **2.50** | |
| 31 | TZSTRI110 | **80.75** | **79.75** | -1.00 | **1.00** | **1.95** | **1.10** | **1.00** | **10.25** | **3.25** | **71.00** | **3.13** | 4.00 | **2.25** | **2.75** | |
| 32 | TZSTRI112 | 80.38 | 82.50 | 2.12 | 1.00 | 1.55 | 0.82 | 1.00 | 9.25 | 4.25 | 62.00 | 3.13 | 5.50 | 3.75 | 2.75 | |
| 33 | TZSTRI114 | 87.75 | 91.00 | 3.25 | 1.00 | 1.10 | 0.47 | 1.00 | 10.00 | 5.25 | 62.25 | 3.13 | 2.50 | 3.00 | 2.00 | |
| 34 | **TZSTRI115** | **84.75** | **87.75** | 3.00 | **1.00** | **1.65** | **0.77** | **1.00** | **10.50** | **3.25** | **70.00** | **3.20** | 1.50 | **1.00** | **1.25** | |
| 35 | **TZISTR25** | **75.75** | **75.50** | -0.25 | **1.00** | **2.25** | **1.05** | **1.00** | **12.00** | **1.25** | **131.00** | **3.20** | 2.00 | **3.75** | **2.00** | |
| 36 | **TZISTR1001** | **79.63** | **78.25** | -1.38 | **1.00** | **2.10** | **1.03** | **1.00** | **11.50** | **1.75** | **120.00** | **2.70** | 1.50 | **3.50** | **2.50** | |
| 37 | TZISTR1003 | 81.38 | 79.50 | -1.88 | 1.00 | 1.53 | 0.91 | 1.00 | 10.00 | 3.75 | 59.00 | 3.20 | 1.50 | 3.50 | 2.25 | |
| 38 | TZISTR1004 | 80.25 | 78.75 | -1.50 | 1.00 | 1.45 | 0.84 | 1.00 | 9.00 | 3.25 | 62.25 | 4.20 | 3.50 | 3.75 | 2.50 | |
| 39 | TZISTR1008 | 81.25 | 79.25 | -2.00 | 1.00 | 1.70 | 0.89 | 1.00 | 9.00 | 3.25 | 62.25 | 4.70 | 1.50 | 2.75 | 2.75 | |
| 40 | TZISTR1011 | 86.50 | 84.75 | -1.75 | 1.00 | 2.05 | 0.96 | 1.00 | 10.00 | 7.25 | 39.75 | 4.20 | 1.50 | 3.00 | 2.00 | |
| 41 | TZISTR1018 | 81.00 | 81.50 | 0.50 | 1.00 | 2.05 | 1.03 | 1.00 | 9.50 | 8.25 | 22.75 | 3.20 | 6.00 | 3.75 | 4.00 | |
| 42 | TZEEI21 | 77.00 | 77.88 | 0.88 | 1.00 | 1.37 | 0.75 | 1.00 | 9.50 | 6.25 | 45.25 | 3.20 | 5.00 | 3.75 | 4.75 | |
| 43 | TZEEI13 | 80.75 | 81.25 | 0.50 | 1.00 | 1.27 | 0.65 | 1.00 | 8.50 | 3.25 | 55.00 | 3.70 | 2.00 | 3.25 | 2.25 | |
| 44 | TZEEI14 | 77.25 | 79.00 | 1.75 | 1.00 | 1.97 | 1.10 | 1.00 | 10.75 | 2.75 | 51.75 | 3.20 | 3.50 | 2.00 | 2.00 | |
| 45 | TZEEI49 | 76.25 | 74.75 | -1.50 | 1.00 | 2.05 | 1.06 | 1.00 | 11.75 | 4.25 | 48.25 | 3.20 | 2.00 | 3.75 | 2.50 | |
| 46 | TZDEEI55 | 78.38 | 76.75 | -1.63 | 1.00 | 1.38 | 0.61 | 1.00 | 9.50 | 4.75 | 51.50 | 2.70 | 2.00 | 3.75 | 2.75 | |
| 47 | **TZDEEI50** | **74.50** | **74.50** | 0.00 | **1.00** | **1.29** | **0.57** | **1.00** | **10.50** | **3.75** | **70.00** | **1.70** | 5.50 | **5.50** | **5.00** | |
| 48 | TZDEEI64 | 70.75 | 74.25 | 3.50 | 1.00 | 1.72 | 0.85 | 1.00 | 10.50 | 4.25 | 52.00 | 2.20 | 2.00 | 2.00 | 4.25 | |
| 49 | CML312 | 74.50 | 72.63 | -1.87 | 1.00 | 2.02 | 0.88 | 1.00 | 8.50 | 6.25 | 27.25 | 3.20 | 2.00 | 3.75 | 1.00 | |
| 50 | CML444 | 77.75 | 77.50 | -0.25 | 1.00 | 1.57 | 0.68 | 1.00 | 7.00 | 4.25 | 42.50 | 2.20 | 1.50 | 3.75 | 1.75 | |
| 51 | CML442 | 79.25 | 80.25 | 1.00 | 1.00 | 1.32 | 0.63 | 1.00 | 6.75 | 4.75 | 45.00 | 1.70 | 2.00 | 3.25 | 2.25 | |
| 52 | **TZDEEI54** | **83.75** | **79.25** | -4.50 | **1.00** | **1.52** | **0.80** | **1.50** | **13.00** | **3.25** | **82.50** | **3.20** | 1.50 | **2.75** | **0.75** | |
| 53 | TZEEI10 | 82.25 | 81.25 | -1.00 | 1.00 | 1.32 | 0.71 | 1.00 | 11.25 | 3.25 | 40.00 | 2.70 | 2.00 | 3.50 | 1.50 | |
| 54 | CML547 | 86.75 | 86.25 | -0.50 | 1.00 | 1.32 | 0.71 | 1.50 | 11.25 | 3.25 | 40.00 | 2.70 | 2.00 | 3.50 | 2.25 | |
| 55 | **CML539** | **75.50** | **73.00** | -2.50 | **1.00** | **1.59** | **0.77** | **1.50** | **8.00** | **3.25** | **70.25** | **3.18** | 5.50 | **0.75** | **1.50** | |
| 56 | **CML440** | **80.50** | **79.25** | -1.25 | **1.00** | **1.62** | **1.17** | **1.00** | **9.50** | **1.25** | **84.50** | **2.70** | 6.50 | **2.75** | **1.75** | |
| 57 | **CML566** | **78.00** | **76.00** | -2.00 | **1.00** | **2.07** | **1.20** | **1.00** | **12.00** | **1.25** | **127.00** | **2.25** | 4.50 | **1.75** | **2.75** | |
| 58 | **CML540** | **77.25** | **76.75** | -0.50 | **1.00** | **2.17** | **0.89** | **1.00** | **11.00** | **3.25** | **108.00** | **2.60** | 4.00 | **1.50** | **2.00** | |
| 59 | **CML545** | **77.50** | **76.00** | -1.50 | **1.00** | **2.15** | **0.87** | **1.00** | **10.00** | **3.25** | **83.00** | **2.68** | 5.00 | **3.75** | **3.50** | |
| 60 | **CML571** | **76.25** | **75.75** | -0.50 | **1.00** | **12.45** | **0.78** | **3.00** | **10.00** | **3.25** | **87.75** | **2.63** | 2.00 | **3.25** | **3.75** | |
| 61 | CML390 | 74.00 | 73.75 | -0.25 | 1.00 | 2.05 | 0.80 | 3.00 | 10.75 | 5.75 | 37.30 | 2.68 | 7.50 | 4.25 | 3.25 | |
| 62 | CLHP0352 | 78.50 | 74.75 | -3.75 | 1.00 | 1.10 | 0.98 | 1.00 | 11.25 | 6.75 | 17.05 | 2.68 | 8.00 | 3.75 | 3.75 | |
| 63 | HA04A-2107-36 | 86.25 | 88.25 | 2.00 | 1.00 | 2.00 | 0.96 | 1.50 | 8.50 | 8.25 | 10.05 | 2.18 | 0.00 | 3.00 | 2.00 | |
| 64 | CLHP0303 | 77.25 | 73.75 | -3.50 | 1.50 | 1.52 | 1.06 | 1.50 | 8.00 | 6.25 | 54.55 | 2.68 | 4.00 | 3.75 | 2.00 | |
| 65 | CLHP0221 | 83.75 | 81.25 | -2.50 | 1.50 | 1.75 | 0.89 | 1.50 | 10.00 | 6.25 | 54.55 | 3.18 | 5.00 | 1.50 | 1.50 | |
| 66 | CLHP0020 | 74.25 | 71.75 | -2.50 | 1.00 | 1.64 | 0.71 | 1.50 | 10.50 | 8.75 | 22.30 | 3.18 | 2.50 | 1.75 | 1.50 | |
| 67 | CLHP0058 | 76.75 | 76.25 | -0.50 | 1.00 | 1.13 | 0.58 | 1.50 | 10.50 | 7.75 | 22.30 | 5.18 | 2.00 | 5.75 | 4.75 | |
| 68 | CKDHL0378 | 80.00 | 79.75 | -0.25 | 1.00 | 1.55 | 0.78 | 1.50 | 10.00 | 4.25 | 28.05 | 5.18 | 2.00 | 4.75 | 5.75 | |
| 69 | CLHP0312 | 76.25 | 78.00 | 1.75 | 1.00 | 1.35 | 0.95 | 1.50 | 8.50 | 2.75 | 35.55 | 3.18 | 1.50 | 3.75 | 3.75 | |
| 70 | CLHP0310 | 72.75 | 72.50 | -0.25 | 1.00 | 1.20 | 0.77 | 1.50 | 8.00 | 5.75 | 23.55 | 2.68 | 2.50 | 3.75 | 2.75 | |
| 71 | **CLHP0003** | **72.75** | **74.25** | 1.50 | **1.00** | **1.85** | **0.88** | **1.00** | **12.75** | **5.25** | **90.80** | **2.68** | 7.00 | **2.00** | **2.50** | |
| 72 | **CKDHL0467** | **82.00** | **82.75** | 0.75 | **1.00** | **1.40** | **1.04** | **1.00** | **13.50** | **5.25** | **96.80** | **2.68** | 3.00 | **4.25** | **3.50** | |
| 73 | CLHP00378 | 75.25 | 74.25 | -1.00 | 1.00 | 1.10 | 0.72 | 1.00 | 8.25 | 7.25 | 25.05 | 2.68 | 4.00 | 4.50 | 4.50 | |
| 74 | CLHP0156 | 73.25 | 72.50 | -0.75 | 1.50 | 2.28 | 0.86 | 1.00 | 11.75 | 7.75 | 26.30 | 2.68 | 3.00 | 2.50 | 4.25 | |
| 75 | CLHP0113 | 77.25 | 76.00 | -1.25 | 1.50 | 1.25 | 0.92 | 1.00 | 12.00 | 8.25 | 39.05 | 2.68 | 2.00 | 2.75 | 2.50 | |
| 76 | CLHP03302 | 74.00 | 71.75 | -2.25 | 1.00 | 0.90 | 0.47 | 1.00 | 8.25 | 8.75 | 46.05 | 2.68 | 2.50 | 4.25 | 2.25 | |
| 77 | **CLHP0404** | **74.75** | **74.25** | -0.50 | **1.00** | **2.07** | **0.75** | **1.00** | **10.00** | **6.25** | **137.35** | **3.18** | 5.00 | **6.00** | **4.00** | |
| 78 | **CLHP0343** | **79.13** | **81.00** | 1.87 | **1.00** | **1.37** | **0.92** | **1.00** | **11.50** | **2.25** | **101.10** | **3.68** | 2.50 | **5.00** | **5.25** | |
| 79 | CZL1380 | 80.25 | 79.25 | -1.00 | 1.00 | 1.23 | 0.79 | 1.00 | 9.50 | 5.25 | 54.85 | 3.68 | 5.00 | 5.25 | 5.50 | |
| 80 | CLHP0326 | **72.75** | **72.25** | -0.50 | **1.00** | **1.60** | **0.72** | **1.50** | **8.50** | **5.25** | **85.35** | **3.68** | 2.00 | **3.25** | **4.75** | |
| 81 | **CZL99017** | **81.75** | **82.25** | 0.50 | **1.00** | **1.70** | **0.68** | **1.50** | **10.75** | **2.25** | **71.10** | **3.18** | 5.50 | **3.50** | **3.00** | |
| 82 | TZEEI34 | 79.25 | 80.25 | 1.00 | 1.00 | 1.26 | 0.58 | 1.50 | 10.25 | 4.25 | 60.10 | 3.18 | 6.50 | 4.00 | 2.25 | |
| 83 | CLHP0049 | 78.25 | 79.25 | 1.00 | 1.00 | 1.25 | 0.47 | 1.50 | 5.50 | 5.25 | 60.35 | 3.18 | 2.50 | 3.25 | 2.50 | |
| 84 | CLHP00478 | 77.75 | 75.25 | -2.50 | 1.00 | 1.60 | 0.55 | 1.00 | 5.25 | 5.75 | 57.35 | 2.68 | 2.00 | 2.50 | 2.00 | |
| 85 | CLHP00286 | 77.88 | 79.50 | 1.62 | 1.00 | 1.25 | 0.70 | 1.00 | 9.75 | 5.75 | 57.60 | 3.68 | 3.00 | 3.25 | 2.00 | |
| 86 | CML451 | 85.75 | 86.25 | 0.50 | 1.00 | 1.65 | 0.84 | 1.00 | 11.25 | 4.25 | 62.85 | 3.68 | 4.50 | 4.50 | 2.50 | |
| 87 | CLHP0302 | 82.75 | 79.75 | -3.00 | 1.00 | 1.40 | 0.79 | 2.00 | 13.75 | 4.25 | 32.10 | 3.18 | 3.50 | 4.25 | 3.00 | |
| 88 | CLHP0364 | 80.25 | 80.25 | 0.00 | 1.00 | 1.45 | 0.57 | 2.00 | 12.50 | 5.25 | 39.35 | 3.68 | 4.50 | 3.75 | 2.75 | |
| 89 | CLHP0350 | 77.25 | 78.25 | 1.00 | 1.00 | 1.85 | 0.62 | 1.00 | 10.50 | 6.25 | 40.85 | 3.18 | 2.00 | 3.25 | 3.25 | |
| 90 | CLHP00294 | 88.50 | 85.75 | -2.75 | 1.00 | 1.07 | 0.47 | 1.50 | 9.25 | 7.25 | 25.10 | 3.18 | 2.50 | 3.50 | 3.00 | |
| 91 | CLHP0005 | 84.25 | 85.75 | 1.50 | 1.00 | 1.10 | 0.32 | 1.50 | 8.75 | 6.25 | 67.10 | 6.68 | 5.50 | 5.75 | 2.50 | |
| 92 | CLHP0022 | 71.75 | 73.88 | 2.13 | 1.00 | 1.45 | 0.45 | 1.00 | 9.00 | 5.25 | 44.60 | 9.68 | 2.00 | 4.00 | 2.00 | |
| 93 | **CML304** | **79.25** | **79.63** | 0.38 | **1.00** | **1.44** | **0.75** | **1.00** | **12.00** | **4.75** | **151.00** | **3.18** | 2.00 | **4.75** | **2.75** | |
| 94 | **TZISTR1174** | **83.75** | **82.00** | -1.75 | **1.00** | **1.68** | **0.91** | **1.00** | **11.00** | **1.75** | **112.75** | **4.18** | 4.50 | **5.00** | **3.50** | |
| 95 | **TZISTR1205** | **81.00** | **83.00** | 2.00 | **1.00** | **2.21** | **1.00** | **1.00** | **9.50** | **1.75** | **129.00** | **4.18** | 1.00 | **3.75** | **2.50** | |
| 96 | **TZSTRI113** | **74.50** | **73.00** | -1.50 | **1.00** | **1.41** | **0.90** | **1.00** | **9.00** | **1.75** | **111.75** | **2.68** | 3.50 | **3.75** | **2.75** | |
| 97 | **TZISTR1119** | **78.75** | **77.00** | -1.75 | **1.00** | **1.81** | **0.95** | **1.00** | **10.50** | **3.75** | **135.75** | **3.68** | 4.50 | **5.50** | **3.50** | |
| 98 | **TZISTR1015** | **80.00** | **80.50** | 0.50 | **1.00** | **1.51** | **0.92** | **1.00** | **9.50** | **3.75** | **74.50** | **3.68** | 4.00 | **1.25** | **3.50** | |
| 99 | **ZM1421** | **82.38** | **80.75** | -1.63 | **1.00** | **2.10** | **0.95** | **1.50** | **10.71** | **2.25** | **88.00** | **2.63** | 2.00 | **1.75** | **3.25** | |
| 100 | **B.King/1421** | **81.00** | **77.75** | -3.25 | **1.00** | **2.35** | **1.05** | **1.50** | **11.71** | **3.25** | **91.75** | **2.63** | 4.50 | **2.25** | **2.25** | |
| 101 | Hickory/1421 | 79.25 | 77.75 | -1.50 | 1.00 | 2.40 | 1.18 | 1.50 | 7.96 | 7.25 | 39.75 | 2.63 | 5.00 | 2.00 | 2.50 | |
| 102 | Kep/1421 | 72.50 | 73.50 | 1.00 | 1.00 | 2.10 | 1.12 | 2.00 | 8.21 | 5.75 | 34.75 | 2.63 | 4.00 | 2.50 | 2.75 | |
| 103 | **Shesha/1421** | **71.50** | **70.75** | -0.75 | **1.00** | **1.82** | **0.88** | **1.50** | **10.71** | **1.75** | **112.25** | **4.63** | 2.50 | **4.00** | **2.75** | |
| 104 | **ZM1423** | **70.25** | **71.88** | 1.63 | **1.00** | **2.17** | **0.94** | **1.00** | **13.71** | **1.25** | **144.25** | **4.63** | 2.50 | **1.75** | **2.50** | |
| 105 | **N.Choice/1421** | **81.25** | **75.25** | -6.00 | **1.00** | **1.62** | **0.85** | **1.50** | **10.96** | **1.75** | **133.25** | **3.13** | 3.50 | **4.00** | **3.00** | |
| 106 | NC.QPM/Z.DPLO | 82.50 | 81.25 | -1.25 | 1.00 | 1.77 | 0.86 | 1.50 | 11.25 | 4.25 | 68.00 | 3.20 | 5.00 | 2.75 | 1.25 | |
| 107 | **STR-SYN-Y2** | **85.25** | **85.25** | 0.00 | **1.00** | **1.60** | **0.80** | **1.00** | **11.25** | **3.25** | **126.85** | **8.18** | 3.50 | **3.25** | **2.50** | |
| 108 | Z. Diplo.BC4C3-W-DT C1 | 87.75 | 87.75 | 0.00 | 1.00 | 0.98 | 0.82 | 1.00 | 12.25 | 3.25 | 60.35 | 6.68 | 2.50 | 4.75 | 4.25 | |
| 109 | TZBSTR (Susceptible)(RE) | 84.25 | 87.75 | 3.50 | 1.00 | 1.65 | 0.66 | 1.00 | 10.50 | 4.25 | 33.60 | 8.18 | 1.50 | 4.75 | 4.25 | |
| 110 | STR-SYN-W1 | 79.00 | 79.00 | 0.00 | 1.00 | 2.12 | 0.94 | 1.50 | 10.50 | 2.75 | 51.10 | 8.18 | 5.00 | 3.25 | 3.25 | |
| 111 | **DTSTR-W SYN13** | **89.25** | **88.50** | -0.75 | **1.00** | **0.98** | **0.75** | **1.50** | **10.00** | **3.75** | **115.35** | **4.68** | 5.50 | **3.50** | **2.50** | |
| 112 | **DTSTR-Y SYN15** | **83.75** | **84.25** | 0.50 | **1.00** | **1.78** | **0.65** | **1.00** | **9.00** | **6.25** | **87.35** | **3.18** | 4.50 | **4.00** | **2.75** | |
| 113 | ((IWD C3 SYN*2/(White DT STR Syn))-DT C1 | 84.25 | 84.50 | 0.25 | 1.00 | 1.48 | 0.65 | 1.00 | 8.50 | 6.25 | 57.10 | 3.18 | 3.50 | 3.75 | 3.00 | |
| 114 | DTSTR-W SYN11 | 87.25 | 89.75 | 2.50 | 1.00 | 1.73 | 0.73 | 1.50 | 8.00 | 3.75 | 45.10 | 2.68 | 3.00 | 2.75 | 1.75 | |
| 115 | SAMMMZ16 | 85.75 | 84.75 | -1.00 | 1.00 | 1.93 | 1.03 | 2.00 | 8.50 | 3.75 | 56.50 | 3.18 | 4.50 | 2.75 | 0.75 | |
| 116 | (TZEOMP5C7/TZECOMP3DTC2) C2 | 84.50 | 82.75 | -1.75 | 1.00 | 1.33 | 0.96 | 2.00 | 9.25 | 5.75 | 59.50 | 3.68 | 3.00 | 3.75 | 2.25 | |
| 117 | ((TZL COMP1-W C6*2/(White DT STR Syn))-DT C1 | 87.75 | 88.75 | 1.00 | 1.00 | 1.73 | 0.66 | 2.00 | 9.25 | 5.75 | 59.50 | 3.68 | 4.50 | 3.75 | 3.25 | |
| 118 | TZCOM1/ZDPSYN | 79.25 | 75.75 | -3.50 | 1.00 | 1.66 | 0.31 | 2.00 | 11.50 | 3.25 | 62.50 | 3.18 | 5.50 | 4.75 | 3.75 | |
| 119 | Colorado/1421 | 77.75 | 79.63 | 1.88 | 1.00 | 1.63 | 0.49 | 2.00 | 10.50 | 1.25 | 58.75 | 4.68 | 7.50 | 5.25 | 5.25 | |
| 120 | M.Pearl/DT-STR | 87.25 | 87.75 | 0.50 | 1.00 | 0.73 | 0.65 | 1.00 | 9.00 | 3.25 | 57.25 | 5.68 | 2.00 | 6.50 | 5.00 | |
| 121 | Z.diplo-BC4-C3-W/DOGONA-1/Z.diplo-BC4-C3-W | 79.13 | 78.75 | -0.38 | 1.00 | 1.34 | 0.75 | 0.50 | 9.75 | 6.25 | 47.00 | 4.18 | 2.00 | 2.25 | 2.50 | |
| 122 | NC.QPM/DT-STR | 79.65 | 80.75 | 1.10 | 1.00 | 1.31 | 0.50 | 0.50 | 9.75 | 6.25 | 47.00 | 3.68 | 5.00 | 5.50 | 2.00 | |
| 123 | ZM1421/DT-STR | 82.50 | 80.75 | -1.75 | 1.00 | 1.32 | 0.50 | 1.00 | 10.00 | 3.75 | 58.25 | 3.68 | 4.00 | 3.75 | 4.00 | |
| 124 | **DTSTR-Y SYN14** | **80.13** | **79.75** | -0.38 | **1.00** | **1.36** | **0.75** | **1.50** | **11.50** | **1.75** | **93.25** | **3.68** | 1.00 | **3.75** | **3.75** | |
| 125 | **ZM1423/Z.DLO** | **81.25** | **83.25** | 2.00 | **1.00** | **12.41** | **1.03** | **1.00** | **10.75** | **4.75** | **96.75** | **2.68** | 5.00 | **3.75** | **2.75** | |
| 126 | **(2*TZECOMP3DT/WhiteDTSTRSYN) C2** | **77.25** | **79.25** | 2.00 | **1.00** | **1.21** | **0.91** | **1.00** | **11.25** | **1.75** | **72.50** | **2.68** | 2.50 | **4.75** | **3.75** | |
|  | **Trial statistics** | **DA** | **DS** | **ASI** | **EPP** | **PLHT** | **EHT** | **HUSK** | **CL** | **EASP** | **GY** | **SEC8** | **SEC10** | **SDR8** | **SDR10** | |
| Inbred lines | | | | | | | | | | | | | | | |  |
|  | Minimum | 70.75 | 71.75 | 0.00 | 1.00 | 0.9 | 0.32 | 1.00 | 5.25 | 1.25 | 10.05 | 1.70 | 0.00 | 0.75 | 0.75 | |
|  | Maximum | 93.5 | 92.00 | 4.50 | 2.00 | 12.45 | 1.25 | 3.00 | 13.75 | 8.75 | 151 | 9.68 | 9.00 | 6.00 | 5.75 | |
|  | Mean | 79.52 | 79.03 | 1.52 | 1.04 | 1.75 | 0.81 | 1.23 | 10.18 | 4.51 | 63.89 | 3.25 | 3.66 | 5.25 | 2.75 | |
|  | **OPVs checks** | | | | | | | | | | | | | | |  |
|  | Minimum | 70.25 | 71.88 | 0.00 | 1.00 | 0.73 | 0.31 | 0.50 | 8.00 | 1.25 | 33.60 | 2.68 | 1.00 | 1.75 | 0.75 | |
|  | Maximum | 89.25 | 71.88 | 3.50 | 1.00 | 12.41 | 1.03 | 2.00 | 13.71 | 6.25 | 144.25 | 8.18 | 7.50 | 6.50 | 5.25 | |
|  | Mean | 82.53 | 82.81 | 1.26 | 1.00 | 2.01 | 0.74 | 1.30 | 13.71 | 3.93 | 70.81 | 4.43 | 3.77 | 3.86 | 3.05 | |
|  | **Hybrids checks** | | | | | | | | | | | | | | |  |
|  | Minimum | 71.50 | 70.75 | 0.50 | 1.00 | 1.62 | 0.49 | 1.50 | 7.96 | 1.25 | 34.75 | 2.63 | 2.00 | 1.75 | 2.25 | |
|  | Maximum | 82.38 | 80.75 | 6.00 | 1.00 | 2.40 | 1.18 | 2.00 | 11.71 | 7.25 | 133.25 | 4.68 | 7.50 | 5.25 | 5.25 | |
|  | Mean | 77.95 | 76.48 | 2.03 | 1.00 | 2.00 | 0.93 | 1.64 | 10.11 | 3.32 | 79.79 | 3.28 | 4.14 | 3.11 | 3.11 | |

DA= days to 50% anthesis, DS= days to 50% silking, ASI= anthesis-silking interval, EPP = ear per plant, PLHT= plant height, EHT= ear height, HUSK= husk cover, CL= cob length, EASP= ear aspect, GY= grain yield, SEC8= *Striga* emergence counts eight weeks after sowing, SEC10= *Striga* emergence counts ten weeks after sowing, SDR8= *Striga* damage rating eight weeks after sowing, and SDR10= *Striga* damage rating 10 weeks after sowing, m= meter, cm= centimetre, g= gramme. Boldface entries denote best-performing genotypes.

Supplemental Table 4: Genetic distance found between 126 maize genotypes based on 16000 SNP markers.
